# Origin of minicircular mitochondrial genomes in red algae

**DOI:** 10.1038/s41467-023-39084-2

**Published:** 2023-06-08

**Authors:** Yongsung Lee, Chung Hyun Cho, Chanyoung Noh, Ji Hyun Yang, Seung In Park, Yu Min Lee, John A. West, Debashish Bhattacharya, Kyubong Jo, Hwan Su Yoon

**Affiliations:** 1 Department of Biological Sciences, Sungkyunkwan University, Suwon, 16419 Korea; 2 Department of Chemistry, Sogang University, Seoul, 04107 Korea; 3 School of Biosciences 2, University of Melbourne, Parkville, Victoria, 3010 Australia; 4 Department of Biochemistry and Microbiology, Rutgers University, New Brunswick, 08901 USA

**Keywords:** Comparative genomics, Genome evolution, Mitochondrial genome, Phylogenomics

## Abstract

Eukaryotic organelle genomes are generally of conserved size and gene content within phylogenetic groups. However, significant variation in genome structure may occur. Here, we report that the Stylonematophyceae red algae contain multipartite circular mitochondrial genomes (i.e., minicircles) which encode one or two genes bounded by a specific cassette and a conserved constant region. These minicircles are visualized using fluorescence microscope and scanning electron microscope, proving the circularity. Mitochondrial gene sets are reduced in these highly divergent mitogenomes. Newly generated chromosome-level nuclear genome assembly of *Rhodosorus marinus* reveals that most mitochondrial ribosomal subunit genes are transferred to the nuclear genome. Hetero-concatemers that resulted from recombination between minicircles and unique gene inventory that is responsible for mitochondrial genome stability may explain how the transition from typical mitochondrial genome to minicircles occurs. Our results offer inspiration on minicircular organelle genome formation and highlight an extreme case of mitochondrial gene inventory reduction.

## Abstract (web summary)

While the organelle genome is commonly considered to be a single circular DNA molecule, extensive variation exists. Here, the authors report multipartite minicircular genomes in red algae and indicate an origin driven by recombination due to loss of DNA replication, recombination, and repair genes.

## Introduction

Organelle genomes (e.g., mitogenomes and plastomes) that are derived from bacteria typically consist of single, circular DNA molecules. However, significant variation in genome size, structure and gene content exists among different eukaryote lineages^1–4^, including algae in the Archaeplastida^5^. Among these departures from the norm are multipartite or fragmented genomes whereby either several different circular (i.e., minicircles) or linear DNAs encode the organelle gene inventory. Examples are the Euglenozoa, where three distinctive variants exist: maxi- and minicircles in Kinetoplastea^6,7^, minicircles in Diplonemea^8^, and linearly fragmented mitogenomes in Euglenida^9^. Minicircular mitogenomes have been reported in many other lineages^10–15^. In the case of plastids, peridinin-containing dinoflagellates contain minicircular genomes^16^. In contrast, multiple linear ptDNA molecules are found in the green algal order, Cladophorales^17^.

Several extraordinary organelle genomes exist in the Archaeplastida with genome expansion having occurred both in green and red algae, primarily through the proliferation of repeats and transposable elements, including group II introns^18–24^. Multipartite plastomes occur in Cladophorales (Ulvophyceae) species^17^ and in *Koshicola spirodelophila* (Chaetopeltidales, Chlorophyceae)^12^. The plastome of *K. spirodelophila* is tripartite with >10 genes encoded on each partition, whereas the Cladophorales contain linear hairpin chromosomes that encode 1–3 genes on each fragment, but 1–5 fragmented coding regions within the same or different chromosomes. These plastid genes are highly diverged when compared to the expected sequence divergence rate and gene content is greatly reduced relative to other green algal lineages. Unlike the diverse organelle genome types present in the green lineage, fragmented organelle genomes have not yet been reported from the red algae (Rhodophyta).

Here, we provide the evidence of minicircular mitogenomes in the red algal class Stylonematophyceae, comprising 50 species that are either unicellular or pseudofilamentous with thick mucilaginous cell walls (https://www.algaebase.org/). Mitochondria in the Stylonematophyceae have tubular cristae^25^ and along with the Compsopogonophyceae and Rhodellophyceae, an endoplasmic reticulum-mitochondria-Golgi association doest not occur^25–27^. Using long-read sequencing data (i.e., Oxford Nanopore and PacBio HiFi), we assembled complete minicircular mitogenomes in *Rhodosorus marinus* CCMP1338 and *Chroodactylon ornatum* JAW4256, in addition to a pseudo-chromosomal nuclear genome assembly from *R. marinus*. Using short-read sequencing data from five additional Stylonematophyceae species (*Tsunamia transpacifica* JAW4874, *Rufusia pilicola* O7031, *Stylonema alsidii* JAW4424, *Chroothece mobilis* SAG104.79, and *Bangiopsis subsimplex* UTEX LB2854), we identified that Stylonematophyceae contain minicircular mitogenomes. Each mitogenome consists of a gene- and species-specific cassette and a species-specific constant region. These minicircles were directly visualized using fluorescence microscope (FM) and scanning electron microscope (SEM), providing conclusive optical evidence of minicircle and its circularity. Minicircle GC content has dramatically increased, not only in coding sequences (CDSs) but also in non-coding regions (NCRs). We demonstrate the Stylonematophyceae has unique gene inventory controlling organelle genome stability that is different from other red algae. The formation of hetero-concatemers through recombination may explain the origin and evolution of minicircles, not only in the Stylonematophyceae, but generally in other eukaryotes.

## Results and discussion

### Minicircular mitogenomes in the Stylonematophyceae

**Fig. 1:**
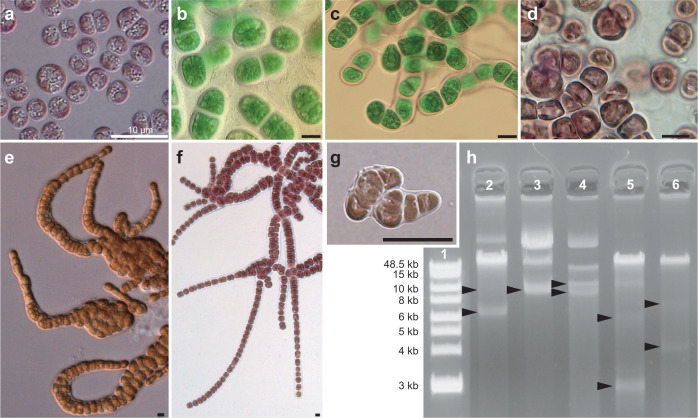
Light microscopy images and genomic DNA from Stylonematophyceae species visualized using agarose gel electrophoresis. **a** *Rhodosorus marinus* CCMP1338. **b** *Chroothece mobilis* SAG104.79. **c** *Chroodactylon ornatum* JAW4256. **d** *Tsunamia transpacifica* JAW4874. **e** *Bangiopsis subsimplex* UTEX LB2854. **f** *Stylonema alsidii* JAW4424. **g** *Rufusia pilicola* O7031. Each panel images **a**–**g** is representative of *n* = 10 biological replicates. All scale bars indicate 10 µm. **h** Genomic DNAs visualized on an agarose gel. LMW DNA bands are indicated by arrows. Gel image was cropped to exclude regions not containing DNA bands and unnecessary ladders. Lane 1: ladder, lane 2: *B. subsimplex*, lane 3: *T. transpacifica*, lane 4: *C. ornatum*, lane 5: *R. marinus*, and lane 6: *S. alsidii*.

**Fig. 2:**
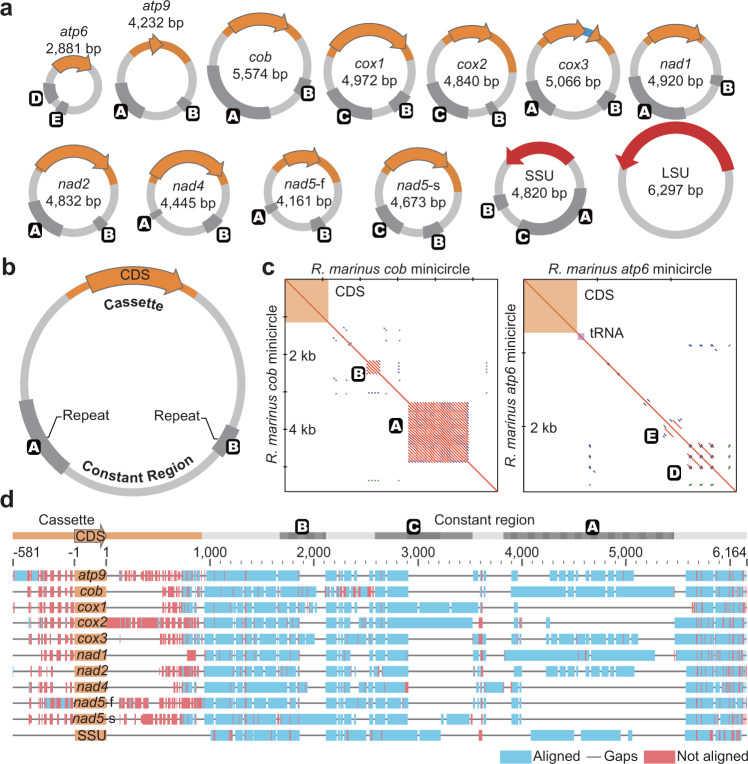
Structure of mitogenomes in the Stylonematophyceae. **a** Thirteen minicircles from the *R. marinus* mitochondrion are drawn to scale. Repeat regions are denoted by letter A, B, C, D, and E. Whether minicircles share a constant region or not can be inferred from the type of repeat region. In *R. marinus*, three different constant regions reside in *atp6*, *atp9*-SSU, and LSU minicircles, respectively. **b** Schematic view of the structure of minicircles. All minicircles comprise a constant region and a gene-specific cassette. A cassette consists of one CDS and a short flanking NCR. Repeat regions are denoted by letter A and B. **c** Dot plots of self-alignments of *cob* and *atp6* minicircles in *R. marinus*. Letters indicate repeat regions. As indicated by the plots, there is no homology between different types of repeat regions. Short matches are colored blue or green whereas matches over 100 bp are colored red or yellow, depending on a direction. **d** Alignments of minicircles of *R. marinus*. The *atp6* and LSU minicircles are not included, because each of them has different constant region. The blue and red boxes indicate aligned and unaligned regions, respectively. The grey line indicates gaps. The cassettes are not aligned, whereas the constant regions are aligned well. Dark gray regions indicate repeat regions. Length and number of repeat units are indicated by alternating dark gray blocks. For example, three repeat units are present in repeat region C in the *cox1* and *cox2* minicircles, whereas one repeat unit is present in all but the *nad5*-s minicircle. With the exception of repeat region C, repeat exist in tandem. The majority of gaps are found in the repeat region. Note that the red tick does not correctly correspond to scale, because it can arise from only single base mismatch.

Results of agarose gel electrophoresis using total genomic DNA (gDNA) from seven Stylonematophyceae species (Fig. 1; for species information, see Supplementary Fig. 1 and Supplementary Data 1) demonstrates the presence of multiple low-molecular-weight (LMW) DNA fragments. In addition, complete mitogenomes were not assembled using short-read sequencing data, which is unusual for red algae^28–31^. Instead, partial linear contigs were identified in the genome assemblies (Supplementary Fig. 2a). Each of these contigs encodes one or two (mostly one) mitochondrial gene(s) and has large shared regions. To avoid assembly artifacts that can be caused by repetitive sequences, long-read sequencing data were also generated for *R. marinus* and *C. ornatum*. These new mitogenome assemblies comprised ~2–6 kb and ~6–17 kb circular sequences, respectively (Fig. 2a and Supplementary Fig. 2b). Most of the raw reads from Nanopore or HiFi sequencing associated with mitochondrial gene peaked roughly at the same length of the assembled minicircles that were distinct from the other nuclear and plastid gene-containing reads (Supplementary Fig. 3). This result excludes the possibility of assembly artifacts and linear concatemers (Supplementary Note 1).

Minicircles of the Stylonematophyceae share a conserved structure that includes a CDS with a short and gene-specific NCR; and a NCR whose sequence is shared among many minicircles within a species but not between species (Fig. 2b) (hereafter, we will refer to the CDS-flanking region as cassette and conserved NCR as constant region^32^). Minicircles without genes were not detected. Generally, constant regions of minicircles that encode genes ubiquitous in the Stylonematophyceae show high sequence similarity, with a small number of single nucleotide polymorphisms (SNPs) and repeat variation in the constant region (Fig. 2c, d, and Supplementary Fig. 4). There are however some exceptions. For example, for species such as *R. pilicola, T. transpacifica*, and *S. alsidii*, sequences between *atp6* minicircles and other gene minicircles could be partially aligned, whereas in the other species, the sequences of *atp6* minicircles were highly diverged from other minicircles. Although nearly all constant regions contain tandem repeats, we found species-specific structural variation in the constant regions (Fig. 2c, d, Supplementary Figs. 4, and 5).

**Fig. 3:**
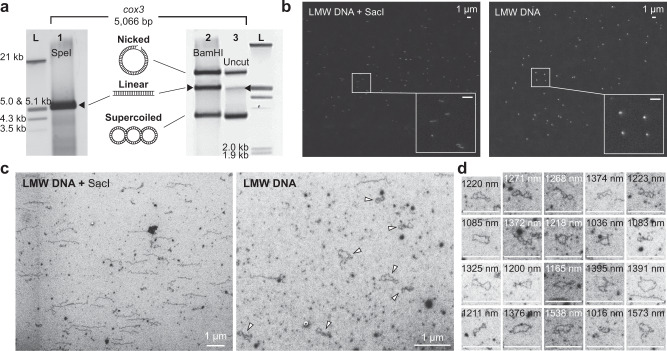
Experimental evidence of minicircles. **a** Southern hybridization (*n* = 1) results using the *cox3* minicircle as probe against *R. marinus* gDNA. Lane 1: Use of the SpeI restriction enzyme showed a band that corresponds to the size of the assembled *cox3* minicircle (linearized DNA; indicated by arrow). Lane 2: Use of BamHI did not result in a single band. However, there was a shift in intensity from supercoiled DNA (lane 2) to linear DNA (lane 3). Lane 3: When minicircles are not treated with a restriction enzyme, three types of DNA conformation (nicked, linear, and supercoiled) are found. Also see Supplementary Fig. 6 and Supplementary Note 2. Lane L: ladder. **b** FM images (*n* = 1) of stained LMW DNA with or without restriction enzyme treatment. Undigested LMW DNA molecules appear as dots (right), whereas digested LMW DNA molecules appear as lines (left). **c** Representative SEM images of LMW DNA with (*n* = 12) or without (*n* = 15) restriction enzyme treatment. A large number of supercoiled and open circular DNA molecules (indicated by arrows) are present when LMW DNA is not digested with a restriction enzyme (right), whereas linear DNA molecules are mostly found when LMW DNA is treated with a restriction enzyme (left). **d** Additional images of supercoiled or open circular LMW DNA molecules from SEM images (*n* = 12). Measured molecule lengths generally agree with the calculated minicircle length (see Supplementary Note 3). All scale bars indicate 1000 nm.

Furthermore, we confirmed the presence of minicircles using Southern hybridization (Fig. 3a, Supplementary Figs. 2b, and 6; see primer information in Supplementary Data 2). Comparison of banding patterns resolved in undigested and restriction enzyme-digested gDNA showed the former to comprise three types of conformation (supercoiled, linear, and open circular), whereas only a linear conformation of expected size is found in the lane with SpeI-digested gDNA. Some restriction enzymes (e.g., BamHI) did not produce the expected banding DNA pattern, however, in the comparison of digested vs. undigested DNA in these cases, only the middle band is variable, which represent the increase in amount of linearized DNA of the predicted size via digestion of supercoiled DNA. Therefore, multiple bands do not necessarily weaken the evidence for a circular topology of mtDNA in Stylonematophyceae (Supplementary Fig. 6 and Supplementary Note 2). This result supports the idea that these red algal mitogenomes are composed of minicircles.

### Evidence of minicircular mitogenomes by DNA molecule visualization with microscopy images

A more direct line of evidence comes from microscopy (Fig. 3). Here, extracted LMW DNA was stained with fluorescent protein-DNA-binding peptides or proteins (FP-DBP) and imaged using FM (Fig. 3b). LMW DNA appears as dots in the image of undigested LMW DNA whereas short lines rather than dots are observed when LMW DNA is treated with SacI restriction enzyme. Similarly, LMW DNA images generated by SEM frequently exhibit open circular and supercoiled DNA molecules when SacI is not treated, whereas linear DNA molecules are apparent after SacI treatment of LMW DNA (Fig. 3c, d and Supplementary Note 3). Consequently, the results provide a conclusive and direct optical evidence of minicircles. Moreover, the images of minicircles demonstrates a powerful advantage of the high resolution of SEM that FM cannot achieve. Although there have been many DNA images obtained using TEM, taking DNA images using TEM is not a simple process. Our approach of visualizing DNA under SEM is simple and straightforward, and thus, it can be a promising tool to confirm DNA structures with nanometer resolutions, as shown in Fig. 3c, d.

### Mitogenomes with divergent gene sequences and reduced gene contents

**Fig. 4:**
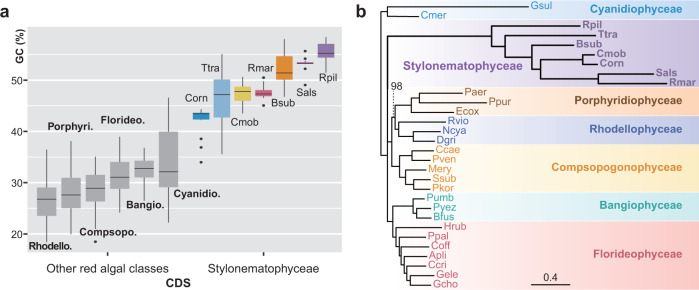
Unique sequence features of Stylonematophyceae mitogenomes. **a** Box plot indicating GC content of mitochondrial CDSs. Box includes the 25th (Q1) to 75th (Q3) percentiles of the data with the median value in a thick line. Upper and lower whiskers indicate values within 1.5 times interquartile range (Q3 – Q1) above Q3 and below Q1, respectively. The Stylonematophyceae (indicated by colors) shows significantly elevated GC content compared to the other red algae (indicated by grey color). Among the Stylonematophyceae, *C. ornatum* has the lowest and *R. pilicola* has the highest GC content. Bangio., Bangiophyceae, *n* = 39 CDSs over 3 species; Bsub, *Bangiopsis subsimplex*, *n* = 13 CDSs; Cmob, *Chroothece mobilis*, *n* = 13 CDSs; Compsopo., Compsopogonophyceae, *n* = 60 CDSs over 5 species; Corn, *Chroodactylon ornatum*, *n* = 13 CDSs; Cyanidio., Cyanidiophyceae, *n* = 25 CDSs over 2 species; Florideo., Florideophyceae, *n* = 91 CDSs over 7 species; Porphyri., Porphyridiophyceae, *n* = 36 CDSs over 3 species; Rhodello., Rhodellophyceae, *n* = 36 CDSs over 3 species; Rmar, *Rhodosorus marinus*, *n* = 11 CDSs; Rpil, *Rufusia pilicola*, *n* = 11 CDSs; Sals, *Stylonema alsidii*, *n* = 11 CDSs; Ttra, *Tsunamia transpacifica*, *n* = 12 CDSs. **b** Topology constraint maximum likelihood phylogenetic tree based on 10 concatenated mitochondrial genes. Provided tree topology is shown in Fig. 5a. Only bootstrap value under 100 is shown. Note the highly diverged Stylonematophyceae sequences. Apli, *Ahnfelta plicata*; Bfus, *Bangia fuscopurpurea*; Ccae, *Compsopogon caeruleus*; Ccri, *Chondrus crispus*; Cmer, *Cyanidioschyzon merolae*; Coff, *Corallina officinalis*; Gele, *Gelidium elegans*; Ggra, *Gracilaria gracilis*; Hrub, *Hildenbrandia rubra*; Paer, *Porphyridium aerugineum*; Ppal, *Palmaria palmata*; Ppur, *Porphyridium purpureum*; Pumb, *Porphyra umbilicalis*; Pyez, *Pyropia yezoensis*. Source data are provided as a Source Data file.

A distinctive feature of Stylonematophyceae mitogenomes is their unexpectedly high GC content and increased rate of non-synonymous substitutions (Fig. 4a, Supplementary Fig. 7, and Supplementary Note 4). Consequently, mitochondrial genes in this lineage are greatly diverged when compared to other red algae that have typical (i.e., ancestral) mitogenome structure and gene content (Fig. 4b and Supplementary Fig. 8). In addition, most organelle genomes have an elevated adenine plus thymine (AT) content due to AT-biased mutations, which is accelerated by stressors such as reactive oxygen species (ROS)^33–36^. Various selection-based hypotheses such as resource availability^37,38^, environmental condition^39^, DNA stability^40^, regulation of gene expression^41,42^, and GC-biased gene conversion (gBGC)^43^ have been proposed, but none of these convincingly explain high GC content. Thus, the driver of high GC content in these genomes is also unknown.

**Fig. 5:**
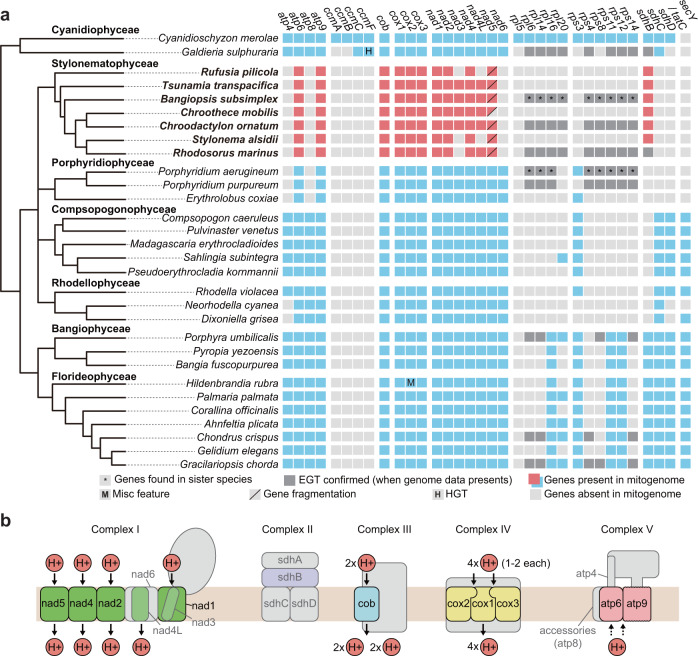
Mitochondrial gene content in 30 red algal species. **a** Red algal phylogeny^18^ showing the gene distribution. Several ATP synthase subunits, NADH dehydrogenase, succinate dehydrogenase, transporters, and all ribosomal protein genes are absent in mitogenomes of the Stylonematophyceae. The diagonal lines in the box for *nad5* indicates gene fragmentation. All species except *C. mobilis* contain a C-terminal region of *nad5*. Note that the gray box indicating EGT is applied if: (1) the species has available genome data, or (2) the species does not have available genome data but a sister species does. In the latter case, boxes were marked with asterisks. Specifically, EGT-derived genes of *Bangiopsis subsimplex and Porphyridium aerugineum* were inferred from the nuclear genomes of *Bangiopsis* sp. CCMP1999 and *Porphyridium purpureum*, respectively. *Bangiopsis* sp. CCMP1999 is not present in the table. Horizontal gene transfer (HGT) and misc feature were referred from^31^ and^28^, respectively. Note that not all red algal mitochondrial genes are shown in the table. EGT, Endosymbiotic gene transfer; HGT, Horizontal gene transfer. **b** Schematic image of mitochondrial complex I-V. Only genes encoded in all Stylonematophyceae are colored, whereas color of seldomly present genes are transparent. In each complex, proton input and output are shown. Complex I-V are based on figures from ref. ^46,47,49,50,52^, respectively.

A total of 13 mitochondrial-encoded proteins were identified in seven Stylonematophyceae species (Fig. 5a and Supplementary Fig. 2). These species have lost all the genes involved in cytochrome c biogenesis and those encoding ribosomal proteins and transporters. Gene sets for mitochondrial complex I, II, and V are partially present, because *nad6, sdhC, sdhD, atp4*, and *atp8* were lost. In some species, *nad3, nad4L*, and *sdhB* are also absent (Fig. 5a). An interesting feature is that all of the remaining minicircle genes have functions related to proton influx (Fig. 5b), although these translocation mechanisms are not thoroughly understood^44,45^. Among seven subunits (nad1–6 and nad4L) in mitochondrial complex I, four subunits (nad1, nad2, nad4, and nad5*)* are involved in the proton input pathway^45,46^ and only these four genes remain in all studied Stylonematophyceae. In complex V, atp4, atp6, and atp9 comprise the F_0_ system with the accessory subunit atp8^47^. Here, protons pass through subunit-a (atp6)^48^ or between subunit-a and the subunit-c ring (atp6 and atp9)^44^. Subunits in complex III and IV (cob and cox1–3) also are involved in proton pumping^49–51^. In this sense, loss of *sdhC* and *sdhD*, or even *sdhB* in mitogenomes may not be lethal because complex II does not play a role in the proton gradient^52,53^. The TCA cycle would not be affected, because sdhA is the key player in this process^54^ and this gene has been lost in all red algal mitogenomes (Supplementary Note 5). The extant mitogenome gene inventories fit well with the hypothesis termed Co-location for Redox Regulation of gene expression (CoRR)^55,56^, whereby genes required for redox regulation are maintained by selection in organelle (mitochondrial or plastid) genomes. Instead of outright loss, the other genes are likely to be transferred and retain their function in the nuclear genome without notable effects on fitness.

Other red algal classes such as the Rhodellophyceae^22^, Compsopogonophyceae^18,21^, and Porphyridiophyceae^23^ contain organelle DNAs that are interrupted by introns that encode ORFs. As an example, Kim*,* et al.^23^ classified intron-encoded proteins (IEPs) in group II introns based on IEP amino acid sequences. However, in the Stylonematophyceae, only a handful of introns are present in mitogenomes and no ORFs were found using BLASTx (Supplementary Fig. 2). We also found evidence of trans-splicing, as previously described in Diplonemea^32^. Notably, the gene *nad5* is fragmented into two pieces (Supplementary Fig. 2), which are encoded on different minicircles (hereafter, *nad5*-f for the N-terminal half and *nad5*-s for the C-terminal half). Analysis of a transcriptome assembly uncovered two RNA transcripts that support the presence of trans-splicing in *R. marinus *(mean coverage = 8.5; minimum coverage = 2). However, we were unable to obtain PCR validation using cDNA. How *nad5* is transcribed and translated requires further inspection. In the case of *C. mobilis*, we were not able to find *nad5*-s. Given that the read coverage of organelle genomes is extremely high, absence of *nad5*-s in *C. mobilis* is unlikely to be a result of sequencing or assembly artifacts. In some species, one of the two gene fragment is found in nuclear genome after the split^57–60^. Additional long-read sequencing is needed to determine if *nad5*-s has been transferred to the nuclear genome or lost.

We also identified the small and large subunit ribosomal RNAs (SSU and LSU rRNA) in *R. marinus* and *C. ornatum* (Fig. 2a and Supplementary Fig. 2b). In the case of tRNA, only a few were encoded on existing minicircles (Supplementary Fig. 2).

For species containing a multipartite mitochondrial genome, segregation of individual minicircles to preserve the gene repertoire in progeny should be under strong selection^61,62^. A simulation study suggested that if copy numbers are not sufficiently high, then selection favors gene linkage^63^ under stochastic DNA segregation. Even though minicircle segregation is well controlled in Kinetoplastea^61^, minicircle loss in laboratory culture were observed^64,65^. Thus, a prerequisite for minicircular genome maintenance is high copy number to prevent loss of function^15^. In this regard, a massive number of minicircles mitigates segregation issues through stochastic assortment in Diplonemea^66^. Similar to Diplonemea, our qPCR analysis demonstrates that mtDNA molecules present in high copy numbers. Specifically, the Stylonematophyceae has a 357.1 ± 152.2-fold copy number difference between mitogenome and nuclear genome, whereas the differences are 3.9-fold and 25.1-fold in the typical (single and circular) mitogenome-containing red algae: *P. purpureum* (Porphyridiophyceae) and *C. merolae* (Cyanidiophyceae), respectively (Supplementary Data 3 and Supplementary Fig. 9). The high mitogenome copy number may allow minicircle gene repertoire maintenance, even under a random segregation model.

### Nuclear genome assembly of *R. marinus* and endosymbiotic gene transfer

**Fig. 6:**
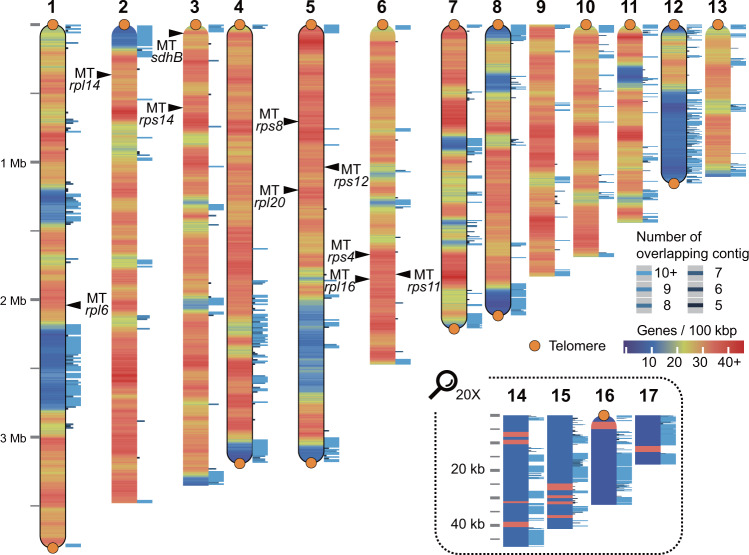
Visualization of the pseudo-chromosomal assembly of *R. marinus* genome. The assembled 17 contigs are arranged according to size. Four small contigs are 20x enlarged. EGT-derived genes and their location on contigs are indicated by arrowheads. The orange dot and rounded end of the contig represent telomere sequences. The black border line indicates the telomere-to-telomere assembly. Heat map shows the density of genes per 100 kb window. The stack bar next to the contig indicates number of overlapping contigs or coverage when contigs are aligned altogether. Source data are provided as a Source Data file.

We assembled the nuclear genome of *Rhodosorus marinus* using long-read sequencing data and investigated genes derived via endosymbiotic gene transfer (EGT) and genes that regulate mitochondrial recombination. The assembled nuclear genome comprises 13 large (>1 Mb) and four small contigs (<40 kb), totaling 31,189,410 bp (Fig. 6). Twelve contigs have at least one telomere and six are assembled telomere-to-telomere. Repeats comprise 15% of genome content (Supplementary Fig. 10). The number of genes and introns are 8392 and 24,821, respectively. The BUSCO^67^ value for core eukaryote gene content is 94.0% (S:91.7%, D:2.3%, F:2.6%, M:3.4%, n:303; eukaryote_odb9) and the RNA-seq mapping rate is 98.6%, supporting high completeness of the newly generated genome.

In the extremophilic red alga, *Galdieria sulphuraria*, nuclear-encoded mitochondrial *sdhB, rpl20*, and *rps12* are EGT-derived^31^. We searched for EGT-derived mitochondrial genes in the *R. marinus* genome assembly and in eight publicly available red algal datasets (see Supplementary Data 1) that include two Stylonematophyceae species (*Bangiopsis* sp. CCMP1999 and *C. ornatum*). Nine out of eleven ribosomal protein genes were found to be nuclear-encoded, however *sdhB* was the only non-ribosomal protein gene that was identified (Supplementary Fig. 11 and Supplementary Data 4). These EGT-derived genes are scattered throughout the genome (arrowheads in Fig. 6). Because some of these ribosomal subunit genes are clustered (Supplementary Data 5), whereas others are not, there may have been independent gene transfers or translocations of genes, post-EGT, of the operon. In the nine species that were investigated, we identified *rpl6, rpl14, rpl16, rps11, rps12*, and *rps14*; whereas *rpl20, rps4*, and *rps8* were absent only in a few species. Absence of these ribosomal subunit genes could arise from the high number of scaffolds (i.e., low assembly quality of reference genome). For example, assembly of the *C. crispus* genome that appears to lack *rps8* comprises 1266 scaffolds, suggesting that many fragmented genes may remain to be identified. Mitochondrial transit peptides were rarely found in proteins encoded by EGT-derived genes (Supplementary Data 6) and the identified N-terminal extensions were not specific to certain proteins. This is not surprising because a cleavable presequence is not necessarily required for mitochondria targeted proteins^68^.

To understand the impact of EGT, we compared the GC content of transferred genes with that of mitochondrial- and other nuclear-encoded genes (Supplementary Fig. 12). Overall gene GC content and 3rd codon GC content (GC3) of EGT-derived genes were statistically different from mitochondrial genes, whereas 1st codon GC content (GC1) showed a significant difference between EGT-derived and nuclear genes, but not between nuclear genes and early EGT-derived genes (i.e., nuclear-encoded mitochondrial ribosomal subunit genes that are shared among all red algal classes; *rpl1-4, rpl6-7, rpl10-11, rpl13-17, rpl21-22, rpl24, rpl46-47, rps1-2, rps4-5, rps7-9, rps11*, and *rps15*). Thus, once a gene is transferred to the nuclear genome, its overall GC content is adjusted to that of nuclear genes via an elevated GC3. However, GC1 change appears to be a relatively slow process.

We could not identify missing genes other than ribosomal protein genes (and one *sdhB*) in the mitogenomes. It is possible that some of the targeted sequences may have diverged too rapidly to be identified using an in silico approach. If so, mutations would have accumulated only on a specific set of genes, because the identified EGT-derived genes do not exhibit high sequence divergence. In contrast, gene function may have been replaced by a duplicated copy of another gene. In plant mitogenomes, several ribosomal gene losses have occurred, followed by functional replacement by other genes. For instance, in plants, either transferred mitochondrial rps19 or chloroplast rps13 substitute the function of lost mitochondrial rps13^69^, a diverged copy of cytosolic rps15A takes the place of mitochondrial rps8^70^, or nuclear-encoded chloroplast rps10 is dual targeted to mitochondria and chloroplasts^71^. However, reports have primarily concerned ribosomal protein genes. Although considered unlikely, in the most extreme case, missing genes could be outright lost, as in the case of the parasitic plant lineage *Viscum* that has lost all mitochondrial complex I genes^72^. This leads to decreased mitochondrial ATP production and increased glycolysis^73^. Alternative oxidases and NAD(P)H dehydrogenases are also assumed to be crucial for electron transfer in *Viscum* (note that these genes are not specifically present in *Viscum*). Using three *Arabidopsis* alternative NAD(P)H dehydrogenase subfamilies (*NDA*, *NDB*, and *NDC*)^74^ as queries, Stylonematophyceae was confirmed to contain more putative alternative NAD(P)H dehydrogenase genes which may contribute to respiration (Supplementary Fig. 13). Nonetheless, because none of the mitochondrial complex was completely lost in the Stylonematophyceae, generating a proton gradient through mitochondrial complexes is likely to remain a major pathway for respiration.

Coding sequences are not the only DNA regions that are transferred to the nuclear genome. Nuclear mitochondrial DNAs (NUMTs)^75^ that primarily comprise non-coding sequences are widespread in the nuclear genomes of diverse eukaryotes^76,77^. Because NUMT insertion progresses via nonhomologous DNA end-joining (NHEJ)^77^, the high copy number and small size of minicircular mitogenomes may favor their incorporation into nuclear DNA. Consistent with this idea, we find that NUMTs are abundant in *R. marinus* (Stylonematophyceae). *R. marinus* has the third largest NUMTs and the proportion of NUMT size to nuclear genome size is the highest among 14 red algae from five classes (Supplementary Fig. 14 and Supplementary Data 7). However, additional long-read based assemblies are needed to allow robust comparisons to be made. In contrast, NUMTs are apparently absent in the *P. purpureum* (Porphyridiophyceae) genome, a species that has a significantly expanded but group II intron-rich mitogenome^23^. Group II introns do not interrupt the nuclear genome of eukaryotes^78^ (Supplementary Note 6).

### Origin of minicircles

**Fig. 7:**
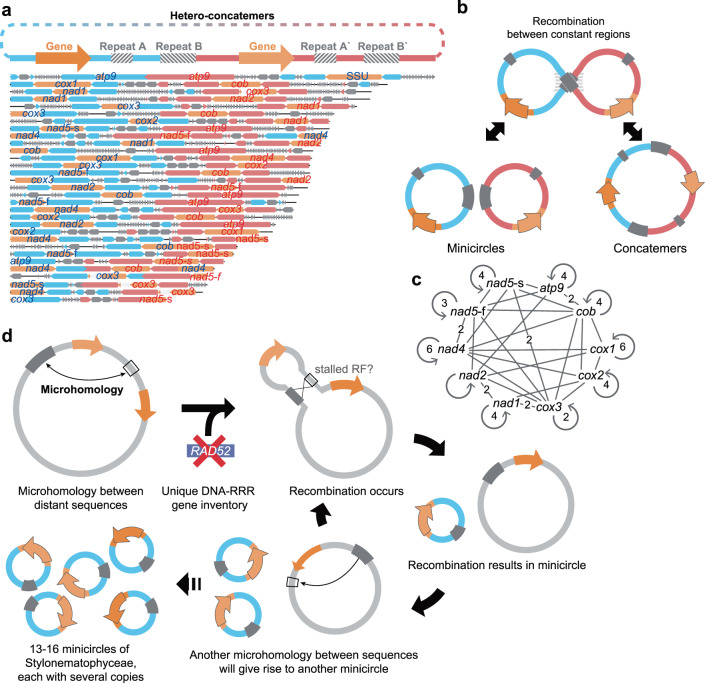
Hetero-concatemers and schematic model of minicircle evolution. **a** Structure and composition of 31 hetero-concatemers out of 67 concatemers in *R. marinus*. Hetero-concatemers with variable lengths consistently have an identical structure, shown at the top. Blue and red boxes represent the NCR of the former and the latter genes. **b** Model for origin of concatemers. Recombination between minicircles might yield concatemers and conversely, recombination within concatemers could yield two independent minicircles. **c** Network representation of gene combinations found in concatemers. Single counted combinations are indicated as edges without numbers. **d** Evolutionary model of minicircle formation. A unique DNA-RRR gene inventory allows frequent MHMR that could drive repeated minicircle formation. A stalled replication fork might have facilitated recombination. Consequently, the large single mitogenomes would be replaced by the minicircles.

We identified concatemers, in which more than two minicircles merged, in long-read data from *R. marinus*. Among 6982 collected (pairwise identity of ≥80% to mitochondrial genes) raw minicircle reads from *R. marinus*, 67 concatemers were present (Fig. 7a and Supplementary Fig. 15). Because the mode of replication is unknown (Supplementary Note 7), there is a possibility that concatemer of the same minicircles (homo-concatemers) may originate via rolling circle replication. To exclude this possibility, we differentiated homo- and hetero-concatemers (which more than two different minicircles merged into) and found 31 high confidence hetero-concatemers. Concatemers have also been reported in lineages containing minicircular organelle genomes, providing evidence of recombination-based merging of two minicircles (Fig. 7b)^79,80^. We assumed that concatemers are not the result of sequencing artifacts for three reasons. First, no concatemers harboring *atp6* and LSU were found (Fig. 7a, c). *atp6* and LSU minicircles do not share a constant region with other gene minicircles. Therefore, concatemers would not be produced from artifacts during ligation. Second, nanopore sequencing allows ssDNA to pass through the pore. Neither template switch during DNA synthesis nor multiple passes of the same ssDNA is permitted. Last, we further verified the presence of hetero-concatemers in vivo using PCR with different primer combinations (Supplementary Fig. 16). Thus, hetero-concatemers provide strong evidence of recombination, likely through homologous recombination.

Why do most minicircles contain nearly identical conserved regions? Although not always^81–83^, this conservation could be essential for minicircle persistence and is therefore found in many independent cases of minicircle formation^10–12,14–16,32,84–87^. Concerted evolution is believed to be primarily responsible for the near-identity of dinoflagellate minicircles^88^. For a minicircle to survive, a functional NCR such as the origin of replication is needed. Because minicircles originated from a single typical mitogenome, the replication origin sequence they retained would have been generally similar with each other. Resulting homologous recombination would ensure that minicircles become more similar over time.

In plants, knock-out of DNA replication, repair, and recombination (DNA-RRR) genes that control organelle genome stability leads to recurrent recombination^89^. The origin of minicircular genomes could be linked to changes in these regulatory genes that disturb mitogenome stability and consequently, allow tandem repeats in the mitogenomes to act as recombination hotspots. Among a list of DNA-RRR genes that were proven to target organelle genome, two candidate genes were identified (Supplementary Data 8 and Supplementary Note 8): *RAD52* and *MSH1*. RAD52 has diverse roles in nuclear genome and mitogenome maintenance through homologous recombination (HR)^90–92^, thereby preventing microhomology-mediated recombination (MHMR). In plants, the RAD52 homolog RAD52-like protein or organellar DNA-binding protein 1 (ODB1) is localized in both the nucleus and mitochondria depending on its C-terminal region^93^ (primarily in mitochondria^94^). Mitochondria in *Arabidopsis odb1* mutants experience frequent MHMR under stressful conditions such as oxidative stress^94^. Surprisingly, RAD52 is absent only in the Stylonematophyceae (Supplementary Fig. 17 and Supplementary Data 8), which is consistent with the idea that recombination drives minicircle formation. MSH1 is dual-targeted to both the mitochondrion and the plastid in plants and suppresses recombination between repeats^95,96^. The impact of MSH1 on minicircle formation is speculative because it is present in all red algae. However, there is a clear discrepancy between the Stylonematophyceae and other red algae (Supplementary Fig. 18). Stylonematophyceae *MSH1* genes are grouped with other eukaryotes in the *MSH1* clade that is hypothesized as HGT-derived gene from giant viruses^97,98^ and distinctively carries GIY-YIG domain. Although currently lacking evidence, it would be interesting if the timing of minicircle formation and *MSH1* replacement in the Stylonematophyceae were to be coincident. For these two genes, we also inspected data in seven red algal genomes from Florideophyceae and Bangiophyceae (those that do not have annotation information; Supplementary Data 7 and Supplementary Note 9). Generally, the result was consistent with the expectation of the presence of *RAD52* and differentially grouped *MSH1* in these taxa. The only exception was the absence of *RAD52* in *Agarophyton vermiculophyllum* (*Gracilaria vermiculophylla*). A unique DNA-RRR gene inventory could therefore stimulate the formation of minicircular mitogenomes by allowing frequent MHMR (Fig. 7d).

Given that concatemers can originate from recombination involving two non-homologous minicircles and a specific DNA-RRR gene inventory, the transition from a typical single circular DNA to multiple minicircles could be mediated by recombination, as previously proposed^9,15,79,99^. Microhomology between repeats and other sequences might stimulate recombination and could have led to minicircle formation (Fig. 7d). Among novel minicircles, those that contain a functional NCR would predominate over those lacking it, as well as the ancestral mitogenome. These minicircles may have proliferated because replication time was reduced for individual mtDNA molecule^15^. This would lead to a lowered reliance on the ancestral mitogenome, that would eventually be lost, as we find in the Stylonematophyceae.

In *Rhopalocnemis*, *WHY2* and *OSB2/3/4* were listed as the genes responsible for minicircular mitogenomes^10^, whereas in Selaginellaceae, which has a multipartite plastome, plastid-targeted RECA1 was lost^100^. In all these cases including the Stylonematophyceae, genes related to homologous recombination are absent, suggesting that illegitimate recombination may drive minicircle formation. In contrast, some louse species lack *mtSSB*^101^ that primarily plays a role in replication^102^. It has been postulated that in the absence of mtSSB, minicircle formation might be favored to deal with a stalled replication fork (RF), because mtSSB prevents replication slippage (copy-choice recombination) in a dose-dependent manner^101,103^. This reasoning is interesting because in the cases described above, *RAD52*, *MSH1*, and *RECA* genes as well as homologous recombination are involved with a stalled RF^96,104–106^. MSH1, in particular the clade that has GIY-YIG domain was postulated to suppress recombination between short repeats during the repair of stalled or collapsed RF^96^. Whether WHY2 and OSB2/3/4 act on stalled RF is unknown but given that these genes are ssDNA-binding proteins^107^ like mtSSB^102^ and RAD52^108^, the likelihood is high. Thus, a stalled RF could be another important component of minicircle formation. Taken together, the formation of minicircles appears to be explained by the loss of genes involved in both homologous recombination and repairing stalled RF, thus trigerring recombination to resolve stalled RF. However, because very few studies dealing with minicircles have discussed DNA-RRR genes, it may be premature to reach a final conclusion and more comprehensive studies are needed.

Red algae are at least a billion years old and have undergone significant remodeling, both with respect to their nuclear and organelle genomes^109^. The Rhodellophyceae contain the largest plastomes known^22^, whereas the Porphyridiophyceae contain expanded mitogenomes^23^. The Compsopogonophyceae have both large mitogenome and plastomes^18,21^. Adding to this list of eccentric features, we demonstrate that the Stylonematophyceae contain minicircular mitogenomes with reduced gene contents and highly diverged coding regions. These minicircular mitogenomes occur in high numbers (at least 100-fold more than nuclear genome; Supplementary Fig. 9) and may locate within mitochondria containing normal tubular cristae^25^. The Stylonematophyceae are as far as we know the first case in Rhodophyta in which every mitochondrial gene is encoded on a different DNA molecule. Our results expand the knowledge base of organelle genome evolution by providing evidence from long-read data and the assembly of nuclear genomes that can be used to infer evolutionary processes. We highlight the extent to which this gene inventory can be reduced and its content fragmented in a lineage that is nonetheless ecologically highly successful. Analysis of gene regulation and functional complementation of missing genes are important next steps in the analysis of the Stylonematophyceae minicircular mitogenomes.

## Methods

### Sample preparation

Culture strains of *Tsunamia transpacifica* JAW4874, *Rufusia pilicola* O7031, *Stylonema alsidii* JAW4424, *Chroodactylon ornatum* JAW4256, *Chroothece mobilis* SAG104.79, *Rhodosorus marinus* CCMP1338, and *Bangiopsis subsimplex* UTEX LB2854 were obtained from J.A. West (School of Biosciences 2, University of Melbourne, Parkville, Victoria 3010, Australia), F.D. Ott (905 NE Hilltop Drive, Topeka, Kansas 66617, USA), The Culture Collection of Algae at Göttingen University, Germany (SAG), The National Center for Marine Algae and Microbiota (NCMA), and the Culture Collection of Algae at The University of Texas at Austin, USA (UTEX), respectively. DY-V medium (added sea salt to 5 ppt) was used for culturing *Rufusia pilicola*. *Chroothece mobilis* and *Chroodactylon ornatum* were cultured in L1 + DY-V (1:1 ratio) medium. The other samples were cultured in L1 medium. Culture flasks were kept under a white LED lamp (7.76 µmol photon m^−2^ s^−1^) at 20 °C in a 12:12 light-dark cycle.

### DNA and RNA extraction

Samples were either collected from 10 µm membrane filters or by centrifugation (30 min, 7197 rcf). Harvested cells were frozen in liquid nitrogen before grinding. Genomic DNAs for Illumina short-read sequencing were extracted using the Exgene Plant SV Kit (General Biosystems, Seoul, Korea) and cleaned up using DNeasy® PowerClean® Pro Cleanup Kit (QIAGEN, Hilden, Germany). For long-read sequencing, genomic DNA was extracted using the manual CTAB protocol with a customized lysis buffer^110^. Harvested samples were placed in a 2 ml tube with bullet and frozen in liquid nitrogen. Then samples were machine ground. After grinding, samples were resuspended by adding 600 µl of CTAB isolation buffer (1% 2-mercaptoethanol added right before usage) and incubated at 65 °C for 20 min. When samples were completely thawed, bullets were removed from the tubes and 6 µl of RNase A was added. After incubation, we centrifuged tubes at 20,817 rcf at 4 °C for 20 min. While not disturbing the pellets, samples were placed into another 2 ml tube and mixed with one volume of phenol:chloroform:isoamyl alcohol (25:24:1, v/v) before centrifugation at 20,817 rcf for 20 min. The aqueous phase was then mixed with one volume of chloroform in a new 2 ml tube and centrifuged at 20,817 rcf at 4 °C for 15 min. After centrifugation, one volume of 100% isopropanol was added and incubated at −20 °C for 30 min. Samples were then centrifuged at 20,817 rcf at 4 °C for 20 min. Precipitated DNA was washed with 70% ethanol and centrifuged again at 20,817 rcf at 4 °C to remove ethanol. Finally, DNA was air-dried and dissolved in 50 µl AE buffer from Exgene Plant SV Kit. Total RNA of *R. marinus* was extracted using RNeasy® Plant Mini Kit (QIAGEN, Hilden, Germany).

### Whole genome sequencing and genome assembly

Library preparation and whole genome sequencing for both short-read and long-read sequencing were carried out by DNA Link Inc. (Seoul, Korea). For short-read sequencing, libraries were prepared using the Truseq Nano DNA Prep Kit (550 bp Protocol) and sequencing was done with the Illumina HiSeq2500 platform according to the protocol using 100 bp paired-end reagents. Long-read sequencing was carried out with Oxford Nanopore platform (ONT GridION) for *R. marinus* (6 kb size selection) and the Pacific Biosciences (PacBio) High-Fidelity (HiFi) sequencing platform for *C. ornatum* (no size selection). RNA-seq for *R. marinus* was done with the Illumina NovaSeq600 platform. The raw data from short-read sequencing were assembled using SPAdes 3.14.1^111^ with —careful pipeline option and those from long-read sequencing were assembled using NextDenovo 2.5.0 (https://github.com/Nextomics/NextDenovo) for nuclear genome of *R*. *marinus*. Assembled NextDenovo contigs were polished 3 times with Pilon 1.22^112^ using short-read mapping data generated by bowtie2 2.3.5.1^113^. For mitogenome assemblies using long-read data, reads that have BLAST hits to mitochondrial CDS were used. The program miniasm 0.3 (r179)^114^ was used to identify the *R. marinus* mitogenome and IPA 1.3.1 (https://github.com/PacificBiosciences/pbipa) was used for *C. ornatum*. In addition, reads that had BLAST hits to the NCR were used to search for empty minicircle reads that do not contain a CDS, however, no contigs were assembled, meaning the collected reads are just fragments of CDS-containing reads. Because minicircles share long conserved region that short-reads cannot discriminate, we used long-read data and NextPolish 1.4.0 (https://github.com/Nextomics/NextPolish) to polish the miniasm-derived contigs. We did not perform polishing on IPA contigs, because HiFi sequencing generates extremely accurate reads. Remaining SNPs and ambiguities were manually corrected using mapping data of long-reads containing CDS. For *C. ornatum*, each sequence from step 10 (10-assemble/p_ctg.fasta) was considered as a minicircle sequence, because the following step of the IPA assembler (polish and purge dups) did not function correctly.

For the short-read data, sorted and verified mitochondrial genes (see below) were used as seeds for NOVOplasty 4.2^115^. Using Geneious (Biomatters, Auckland, New Zealand), generated NOVOplasty contigs were then de novo assembled. Assembled contig that codes any of mitochondrial genes was considered as part of mitochondrial genome. Those contigs were polished (SNP & Indel) with Pilon 1.22^112^, using short-read mapping data generated by bowtie2 2.3.5.1^113^. Trinity 2.11.0^116^ was used to assemble RNA-seq data.

### Sorting and verifying mitochondrial contigs

BLAST 2.2.31+^117^ was used to search for mitochondrial genes. Because mitochondrial gene sequences of the Stylonematophyceae were absent in the National Center for Biotechnology Information (NCBI) database, mitochondrial protein sequences from several red algal species were searched against assembled SPAdes contigs with *e*-value 1e-05 using tBLASTn. All the matched sequences were translated (Genetic code 4^118^) and aligned against NCBI protein database (nr). Sequences that have eukaryotic taxa in top 100 matches were considered as candidate genes. Those that only had prokaryotic taxa in top 100 matches with significantly low identity or query coverage were also selected as possible candidates.

To exclude bacterial contigs from possible mitochondrial contigs, genomic features such as GC content, read coverage and tBLASTn result (top match and identity) of the contig were used as criteria for selection. CDSs of each contig were compared against nr database using default parameters. These candidate contigs were verified manually using phylogenetic analysis. Using translated CDS in candidate contigs as queries, protein sequences from nr database were searched by MMSeqs2^119^ (Version: 330ea3684fd3f985d0127ffe8ca5b3f13053c619) with maximum sensitivity and *e*-value 1e-05.

### Nuclear gene prediction

RNA-seq reads were mapped against the assembled nuclear genome of *R. marinus* using hisat2 (2.2.1)^120^ and STAR 2.7.7a^121^ (--outFilterScoreMinOverLread 0.45 --outFilterMatchNminOverLread 0.45). Mapping information was used as training set of ab initio gene models, performed using BRAKER 2.1.5^122^. Completeness was measured using BUSCO 3.0.2 with the eukaryote_odb9 database^67^, following Cho*,* et al.^123^. *RAD52* was not found in the gene model of *C. crispus* and contaminant assemblies were found in the transcriptome assembly of *C. ornatum*. Therefore, we chose to generate a transcriptome assembly and perform gene modeling using the available RNA-seq data (see Supplementary Data 1). We used Trinity 2.11.0^116^ to obtain the transcriptome assembly. Cd-hit 4.8.1^124,125^ was used to cluster sequences with similarity over 95% and proteins were predicted using Transdecoder 5.5.0 (https://github.com/TransDecoder/TransDecoder). BUSCO^67^ values were: *C. crispus*, C:97.4% [S:27.4%, D:70.0%], F:2.6%, M:0.0%, n:303; and *C. ornatum*, C:96.7% [S:20.5%, D:76.2%], F:1.3%, M:2.0%, n:303. Consequently, we predicted several novel genes that are not present in existing red algal data.

### Comparative analysis of CDSs

The mitochondrial genomes of 23 red algae representing Cyanidiophyceae, Compsopogonophyceae, Porphyridiophyceae, Rhodellophyceae, Bangiophyceae, and Florideophyceae were downloaded from NCBI nucleotide database (nt) and used for the comparison (Supplementary Data 1). Translated sequences of 11 CDSs (*atp6, atp9, cob, cox1, cox2, cox3, nad1, nad2, nad4, nad5*-f and *nad5*-s) were aligned by MAFFT 7.310^126^ and concatenated. From the concatenated alignment, amino acid similarity were calculated by Geneious 10.2.3 using blosum62 matrix^127^ with threshold 1 as well as nucleotide identity. Maximum likelihood phylogenetic tree was built using IQ-TREE 1.6.8^128^. Optimal evolutionary model was automatically chosen after model selection^129^. Pairwise dN/dS calculation was performed by ParaAT 2.0^130^ and KaKs Calculator 2.0^131^.

For species identification of seven Stylonematophyceae, we downloaded *rbcL* sequences of 16 Stylonematophyceae and one Compsopogonophyceae from NCBI and aligned with those of our samples using MAFFT 7.310^126^. IQ-TREE 1.6.8^128^ was used to construct a maximum likelihood phylogenetic tree. Optimal evolutionary model was automatically chosen after model selection^129^.

### Detecting mitochondrial tRNA, rRNA, and other sequences in the nuclear genome

We searched for tRNA genes using ARAGORN 1.2.38^132^. Ribosomal RNAs were initially searched in long-read assemblies of *R. marinus* and *C. ornatum* using barrnap 0.9 (https://github.com/tseemann/barrnap) and RNAmmer 1.2^133^ but no matches were found. We then looked for raw reads reporting BLASTn hits on collected red algal rRNA sequences under diverse *e*-values, up to 100 but the results were not useful. Next, we looked for RNA transcripts of *R. marinus*. Long-reads mapped on each of assembled transcripts were collected, and assembled using minimap2 (2.17-r941)^134,135^ and miniasm 0.3 (r179)^114^. Transcripts whose assembled contig has circular topology were aligned to red algal rRNA sequences and manually inspected. LSU rRNA of *C. ornatum* was detected using BLASTn with LSU rRNA of *R. marinus* as a query but not in the case of SSU rRNA. SSU rRNA minicircle has a constant region that is shared among most of other minicircle in *R. marinus*. Thus, we collect reads that have BLASTn match against constant regions and filtered out reads that have BLASTn match against coding sequences. After the assembly (described above), only one contig that has a circular topology left, a SSU rRNA minicircle of *C. ornatum*. Polishing was performed using the procedure described above.

For coding genes, we downloaded gene sets of six red algae and the transcriptome assembly of two red algae (Supplementary Data 1). As described above, we predicted gene sets for *C. crispus, Bangiopsis* sp. CCMP1999, and *C. ornatum*. We aligned all the mitochondrial genes from 30 species against gene sets of each red alga using DIAMOND 0.9.36.137^136^ to find EGT-derived genes. Then we aligned all the gene sets against the nr database. Hit queries and subjects were aligned using MAFFT 7.310^126^ and a phylogenetic tree was constructed using IQ-TREE 1.6.8^128^.

Genes that control genome stability were selected based on existing data^137–141^. Proteins were downloaded from the NCBI database and aligned against the red algal gene set using BLASTp (*e*-value 1e-03). Hit identity, alignment length, query length, and subject taxon from BLASTp results against NCBI database, as well as alignment and phylogenetic tree were taken into account to determine the presence of a gene. Specifically, for *RAD52*, *RAD52* and its paralog/homolog protein *RAD59*, *RTI1*, and *RAD22*, as well as some other related proteins *MGM101* and *RDM1* were collected from the NCBI database for query and an *e*-value with a maximum of 10 was used. Seven additional red genomes were used for identification of *RAD52* and *MSH1* (Supplementary Data 8 and Supplementary Note 9). Interproscan 5.52–86.0^142^ was used for domain prediction.

Transit peptides were identified using targetP2.0^143^ (-org pl). Statistical tests were performed using the Wilcoxon rank sum test in R 4.0.3. Complete mitogenome sequences were used as BLASTn queries against nuclear genome to find NUMTs. Hits with *e-*value under 1e-04 were considered as NUMTs^144,145^.

### Raw long-read length distribution

Raw long-reads of *R. marinus, C. ornatum, P. purpureum*, and *G. chorda* were aligned against mitochondrial, plastid, and nuclear CDSs of each species (Supplementary Data 1). Plastid CDSs for *R. marinus* and *C. ornatum* were manually annotated. For *P. purpureum* (NC_023133.1) and *G. chorda* (NC_031149.1), plastid genomes were downloaded from the nt database. Reads with hit length over 80% of CDS length and identity over 90% were used. In case of intron-rich genes (e.g., *R. marinus* nuclear genes), hit length over 200 bp was used as criteria. Finally, only CDS that has considerable coverage (differs genome by genome) was used for analysis to reduce noise signal.

### Polymerase chain reaction (PCR) and quantitative PCR (qPCR)

*R. marinus* total DNA was used to confirm hetero-concatemers and primers were designed to target ends of CDSs and toward the NCR, so that NCRs were amplified. *R. marinus* cDNA was used to confirm trans-spliced *nad5* transcript and primers were designed to target 3' end of *nad5*-f and 5' end of *nad5*-s. *R. marinus* cDNA synthesis was performed using First Strand cDNA Synthesis kit (random hexamer primer; Thermo Scientific, Massachusetts, USA). PCR was performed using AccuPower® PCR PreMix kit (BIONEER, Daejeon, Korea). All primer designs were done using a modified version of Primer3 (2.3.7) built in Geneious 10.2.3. PCR conditions consisted of initial denaturation at 95 °C for 3 min, followed by 35 cycles of denaturation at 95 °C for 30 sec, annealing at 55 °C for 30 sec, extension at 72 °C, and a final 7 min extension step at 72 °C. Extension steps take 4.5 min for the former and 1 min for the latter. PCR products were purified with LaboPass^TM^ PCR kit (Cosmo Genetech, Seoul, Korea). Purified PCR products were sequenced using Sanger method by Macrogen Inc. (Seoul, Korea).

The SsoFast^TM^ EvaGreen® Supermix (Bio-Rad, California, USA) was used for the qPCR assays (two replicates) that were run on a CFX96^TM^ system (Bio-Rad, California, USA). Primers were designed to amplify gene-specific 150 bp fragments and were tested in advance to check for primer-dimer formation in no-template control (NTC). Each tube contained 5 µl of supermix, 0.2 µl of forward and reverse primers, 3.6 µl of nuclease free deionized water, and 1 µl of template DNA (final volume 10 µl). Probes for Southern hybridization were used as template DNAs of standard (see Supplementary Data 2 for more information). Starting from concentration of 1 ng/µl, seven 10-fold serial dilution series were prepared. Starting concentration of standard sample of *sdhB* was 0.01 ng/µl because *sdhB* is a nuclear-encoded gene in *R. marinus*. For target samples, 0.06 ng of gDNA extracted using the CTAB method were used. Concentration of gDNA was measured using Qubit® 2.0 Fluorometer and Qubit^TM^ dsDNA BR Assay Kit (Invitrogen, Massachusetts, USA) and all samples underwent the same treatment. Quantitation cycle (C_q_) values were calculated in Bio-Rad CFX Manager 2.1 (C_q_ determination mode = Single Threshold). Copy number was calculated using equation $\tfrac{x\times N_A}{l\times 660\times {10}^{9}}$, where *x* is amount of DNA (ng), *N*_A_ is Avogadro number, and *l* is length of DNA (bp)^146^. PCR efficiency were calculated using equation ${10}^{-\tfrac{1}{k}}-1$, where *k* is the slope of a standard curve^147^. qPCR conditions consisted of initial denaturation at 95 °C for 3 min, followed by 50 cycles of denaturation at 95 °C for 5 sec, annealing and extension at 60 °C for 15 sec.

### Probe synthesis

All fragments for the minicircle DNA genes (*atp6*, *atp9*, *cob*, *cox1*, *cox2*, *cox3, nad1*, *nad5*-s, and *nad5-*f), LSU rDNA gene, and the *sdhB* nuclear gene sequences were prepared from gDNA with specific primers (Supplementary Data 2) by using PCR and were purified using the LaboPass^TM^ PCR kit (Cosmo Genetech, Seoul, Korea) prior to labeling. The digoxigenin (DIG)-labeled probes for Southern blot were synthesized using the DIG-High Prime DNA Labeling and Detection Starter Kit I (Roche Diagnostics, Mannheim, Germany), according to the manufacturer’s instructions.

### Southern blot analysis

For the Southern blot analysis, 1 µg of gDNA from of *R. marinus* was either undigested or digested with each minicircle-suitable restriction enzyme that has only one restriction sites outside the targeted region (Supplementary Fig. 2b). The digestion products were separated using 1% agarose gel electrophoresis in TAE buffer and transferred overnight to a positively charged nylon membrane (Cat. No. 11209299001, Sigma-Aldrich, Missouri, USA) through capillary blotting with 10X Saline-sodium citrate (SSC) buffer. After transfer, the membrane was auto-crosslinked using the Stratagene UV-Stratalinker. The crosslinked membrane was prehybridized, hybridized with the DIG-labeled probes, and then washed. Finally, the hybridized DNA probes were immunodetected with anti-digoxigenin-AP (Fab fragments) and visualized with the colorimetric substrates NBT/BCIP using the DIG-High Prime DNA Labeling and Detection Starter Kit I (Roche Diagnostics, Mannheim, Germany) according to the manufacturer’s instructions. Blot images were stored by photocopying the wet filters.

### Microchannel and positive surface preparation

Polydimethylsiloxane (PDMS) devices and positively charged surfaces were prepared as previously described^148^. In particular, microchannel template was utilized to create two layers on a silicon wafer through repeated photolithography procedures, following the protocol specified in the Kayaku Advanced Materials SU-8 2000 datasheet. First, a silicon wafer was spin-coated with a 20 µm layer of SU-8 2015 photoresist (Kayaku Advanced Materials, Massachusetts, USA) using a spin coater (Midas System SPIN-1200D, Daejeon, Korea). Subsequently, the spin-coated wafer was exposed to 350 nm radiation with an aligner (Midas System MDA-400LJ, Daejeon, Korea) through a mask and developed using an SU-8 developer (Kayaku Advanced Materials, Massachusetts, USA). Next, SU-8 TF 6002 was spin-coated as a second layer on top of the first layer. Since SmartPrint (SmartForce Technologies, La Tronche, France) is compatible with g-line photoresists, SU-8 TF 6002 was used. Following fabrication of the template wafer, the outlet port was created by attaching a tube to the peak area of the triangular section. At last, microchannel template was placed onto a silicon wafer. Then, a mixture (10:1 wt ratio) of PDMS pre-polymer and curing agent (K1 solution, Gwangmyeong, Korea) was poured onto it and incubated at 65 °C for 12 hr. The resulting PDMS layer was peeled from the wafer, and a chamber was created by physically punching a channel into it. The PDMS microchannel underwent oxidation for 30 sec at 100 W in an air plasma generator (Femto Science Cute Basic, Korea). Finally, the PDMS device was washed and stored in deionized water.

Silicon wafers (Wafer market, Yongin, Korea) were purchased with a 30 nm SiO_2_ layer on top. To eliminate the polymer coating, oxidized silicon wafers and glass coverslips were arranged in a Teflon rack and soaked in piranha etching solution (30:70 v/v H_2_O_2_/H_2_SO_4_) for 3 hr. The wafers and coverslips were washed thoroughly with deionized water. Neutral pH of 7 was achieved and confirmed by pH paper. Subsequently, the wafers and coverslips were sonicated in deionized water for 30 min, followed by another round of rinsing with deionized water, to uncover the piranha surface. In the end, solutions with a concentration of 1.1 mM were prepared by adding 150 µl of Q-siloxane in 50% methanol to 250 ml of deionized water. Wafers and coverslips were incubated at 65 °C and 100 rpm for 16 hr. Finally, they were rinsed three times with 99.9% ethanol and stored in 99.9% ethanol.

### DNA molecule visualization under FM and SEM

DNA molecules pre-mixed with FP-DBP^149,150^ were stained with 5% polyvinylpyrrolidone (PVP, molecular weight (MW): 40,000) solution. Stained DNA molecules were elongated and immobilized on a positively charged surface using a PDMS microfluidic device. DNA molecules were imaged under a FM. The microscopy system consisted of an inverted microscope (Olympus IX70, Japan) equipped with 100× Olympus UPlanSApo oil immersion objectives and an illuminated LED light source (SOLA SM 2 light engine, Lumencor, OR). Fluorescence images were captured using a scientific complementary metal-oxide semiconductor (sCMOS) camera (PRIME; Photometrics, AZ) and stored in a 16-bit TIFF format generated by Micro-manager software. In addition, DNA molecules were imaged using field emission SEM (FE-SEM; JSM-7100F, JEOL, Japan). Circular and supercoiled DNA molecules that appear as dots under the FM were confirmed under the SEM.

The length of circular and supercoiled DNA molecules was manually measured using ImageJ^151^. Length of 1 bp is commonly known to be 0.34 nm, however observed length depends on stretching of DNA molecules^148,152^ and may need case-specific conversion factor. For example, Kosar, et al.^153^ used conversion factor of 0.36 nm/bp which was calculated from internal standard. For DNA molecules <10 kb, fractional extensions are <80%^148^. Therefore, we used plasmid with known length (5.2 kb) for correct measurement. Average length of the plasmid was 1233.3 ± 150.6 nm (*n* = 37), which in turn tells that 1 bp is ~0.24 nm for ~5 kb DNA molecules (70.6% fractional extension).

### Reporting summary

Further information on research design is available in the Nature Portfolio Reporting Summary linked to this article.

## Supplementary information

Supplementary Information

Description of Additional Supplementary Files

Supplementary Data 1-8

Reporting Summary

## Data Availability

Short-read data generated in this study have been deposited in the NCBI Sequence Read Archive under BioProject PRJNA778797. Mitogenomes for two species and CDSs for other five species generated in this study have been deposited in the NCBI database under accession numbers OK643888-OK643971, ON716284, ON716285, OP177696, and OP146131. Whole Genome Shotgun project of *R. marinus* generated in this study has been deposited at GenBank under the accession number JAMWBK000000000.1 (GCA_029953675.1) and National Marine Genome Information Center of Korea (http://www.magic.re.kr) under the accession number MA00405. Source data are provided as a Source Data file. The other data generated in this study are deposited in DRYAD database^154^ (10.5061/dryad.tqjq2bw0w). Accession numbers for published genetic data used in the study are provided in the Supplementary Data 1 and 7. Source data are provided with this paper.

## Source data

Source Data

## Code availability

This study does not report custom codes or mathematical algorithms.
