# Supplementary material for: Origin of minicircular mitochondrial genomes in red algae
Source: Nat Commun. 2023 Jun 8;14:3363. doi: 10.1038/s41467-023-39084-2 (PMC10250338; doi:10.1038/s41467-023-39084-2)
Supplement: Supplementary file 1 — Supplementary Information [file 41467_2023_39084_MOESM1_ESM.pdf]

# Supplementary Information

## Origin of minicircular mitochondrial genomes in red algae

## Supplementary Notes

### 1. Read length distribution and mitogenome topology

Size selection was not used for HiFi sequencing of *C. ornatum* total DNA, whereas 6 kb size selection was used for Nanopore sequencing of *R. marinus*. The green and black dashed lines in Supplementary Fig. 3 are the average of reads that contained nuclear or plastid genes and the shaded regions are trajectories of individual nuclear or plastid-containing reads. These are indicators of the normal read length distribution because no reads can exceed the length of the plastid or nuclear genome. As a consequence, the plastid and nuclear read length distribution of *C. ornatum* is broad and flat, along the entire length range, whereas those of *R. marinus* sharply increased at ca. 6 kb.

What if a single read can be longer than a genome? In this case, reads at the size of the genome would accumulate at high levels because majority of these data would converge on the genome size. This is what happens with the mitochondrial data. The length distribution of mitochondrial reads increased dramatically at a given length and then immediately plummeted, implying the full size of the mitogenomes. This result is supported by the LMW bands on the gel (lane 5 in Fig. 1h).

Because inferred certain length corresponds to the assembled length (monomer), it is clear that all, or at least most of minicircles of the Stylonematophyceae are present as monomers, not concatemers.

As evidence against the idea that mitochondrial reads could be different from plastid and nuclear reads for an unknown reason, we also retrieved raw long-read data from *G. chorda* (PacBio RSII) and *P. purpureum* (Oxford Nanopore). All *G. chorda* reads exhibit sharp peaks at 2 kb, likely due to shearing and size selection. In *P. purpureum*, all reads exhibited an even distribution, similar with the cases above. In either case, regardless of where they originate (mitogenome, plastome, or nuclear genome), the trajectories of mitochondrial, plastid, and nuclear reads overlap, which is not the case for Stylonematophyceae.

### 2. Southern hybridization

Circular DNA is present in three conformations: nicked, linear, and supercoiled DNA. When circular DNA is cut only once, linear DNA of the true length remains. Thus, in Southern blot analysis, we should see single bands of the exact length in enzyme-treated lanes and multiple bands in enzyme-

untreated lanes.

Use of three restriction enzymes (PstI, SacI, and BamHI, respectively) did not result in a single band in lanes containing digested DNA, even after lengthy treatment times. Rather, we observed multiple bands, generally three (Lanes 2,5, and 8). Given that the lengths correspond to bands in the uncut DNA lane, it is likely that the upper ones represent nicked and the lower ones represent supercoiled DNA that may resulted from incomplete digestion. The middle band (indicated by the arrow) represents linearized DNA following digestion and its size corresponds to size of the assembled minicircle. Thus, although extra, unexpected bands appeared in our analysis, linear DNA of the predicted size was identified. Furthermore, the intensity of linear, digested DNA is much stronger than the case for uncut DNA (lanes 2 and 3; 5 and 6; 7 and 8), whereas the intensity of the supercoiled DNA band (indicated by asterisk) decreases: i.e., supercoiled DNAs are linearized. In addition, use of SpeI produced the ideal result (single band in lanes 1 and 4). Together, these results provide strong evidence that mtDNAs are present in a circular conformation.

**3. Scanning electron microscope (SEM) image of LMW DNA**

Images of Fluorescence Microscope (FM) and SEM are available in the Dryad database (see Data Availability statement). Calculated from 1 bp = 0.24 nm, lengths should be around 691.4 nm (2,881 bp), 998.6-1,337.8 nm (4,161-5,574 bp), and 1,511.3 nm (6,297 bp). We observed length of open circular and supercoiled DNAs ranges from 1,016-1,573 nm that fits into the calculated range. As gDNA was extracted from axenic cultures, there would be no possibility that the observed minicircles are bacterial plasmid. In conclusion, SEM image confirmed undoubtful physical evidence of minicircle DNAs.

**4. Mitogenomes with High GC Content**

GC contents of red algal mitogenomes (NCBI database) are 22-46%, however those of the Stylonematophyceae are 34-62% with *C. ornatum* having the lowest value (Supplementary Fig. 7a). GC content at the three codon positions (GC1, GC2, and GC3) also differs (Supplementary Fig. 7b and 7c). Typically, in red algae, GC1 is the highest and GC3 the lowest, due to codon degeneracy [4]. However, in the Stylonematophyceae, GC2 has the lowest GC content.

**5. Impacts of the loss of *sdhB, sdhC,* and *sdhD***

*SdhA* encodes a subunit where substrate binding and oxidization (succinate to fumarate), a step in TCA cycle, occurs [5]. This gene is not present in the mitochondrial genome of red algae. Serving as a

membrane anchor, sdhC and sdhD both play roles in ETC but the main subunit is sdhB [6, 7]. This may explain why *sdhC* and *sdhD* is all lost in mitogenomes of the Stylonematophyceae whereas *sdhB* is only absent in mitogenome of *R. marinus*.

**6. Group II introns and NUMTs**

It has been proposed that group II introns are ancestors of spliceosomal introns in nuclear genomes [8, 9]. In living species, with a few exceptions [10, 11], group II introns are absent in nuclear DNA [9, 12, 13, 14]. This also holds true for *P. purpureum* (Porphyridiophyceae). A possible explanation for this trend is that nuclear genes that contain group II introns may fail to be correctly expressed leading to the rapid removal of these intervening sequences [15, 16]. Nevertheless, given that non-coding DNA is the main source of NUMTs [17], there must be another mechanism that explains their origin.

**7. Statistic of concatemers and replication mode**

Yu*, et al.* (2022) showed that minicircle mitogenomes replicate *via* the rolling circle mechanism in *Rhopalocnemis phalloides*. In this species, 2,165/3,241 PacBio mitochondrial reads (66.8%) were concatemers of at least two units, up to 13. Among these, 1,929 of them (89.1%) include homo-concatemers. In stark contrast, we found 67 concatemers (0.96%) from 6,982 Nanopore mitochondrial reads, up to four units (Fig. 7a and Supplementary Fig. 15). Only two concatemers contained three units and one contained four units. A total of 36 concatemers (53.7%) contained the same minicircle. Though there are big differences, these can't be critical evidence of absence of rolling circle mechanism. Thus, mitogenome replication in the Stylonematophyceae is unknown and needs further study to discover.

**8. Phylogenetic trees and alignments of regulatory genes**

Phylogenetic trees and alignments for all the genes are available in the Dryad database (see Data Availability statement). Phylogenetic analysis failed to resolve the relationships among and within clades. A lack of even taxon sampling might explain this result. However, high support for the monophyly of each clade was recovered and branch lengths were comparable, with robust protein alignments. Because gene modeling and functional annotation are generally homology-based, inaccurate phylogenetic relationship alone should not undermine the findings we report.

**9. Additional seven red algal genomes**

These red algal genome data (Supplementary Data 7) do not have annotation information, and thus

were excluded from the analysis. *RAD52* was not found in the genome of *Gracilaria vermiculophylla.* Transcriptome data are needed to provide a more convincing result. Unfortunately, RNA-seq data are lacking from *G. vermiculophylla*, therefore we could not verify the abasence of *RAD52* in this species.

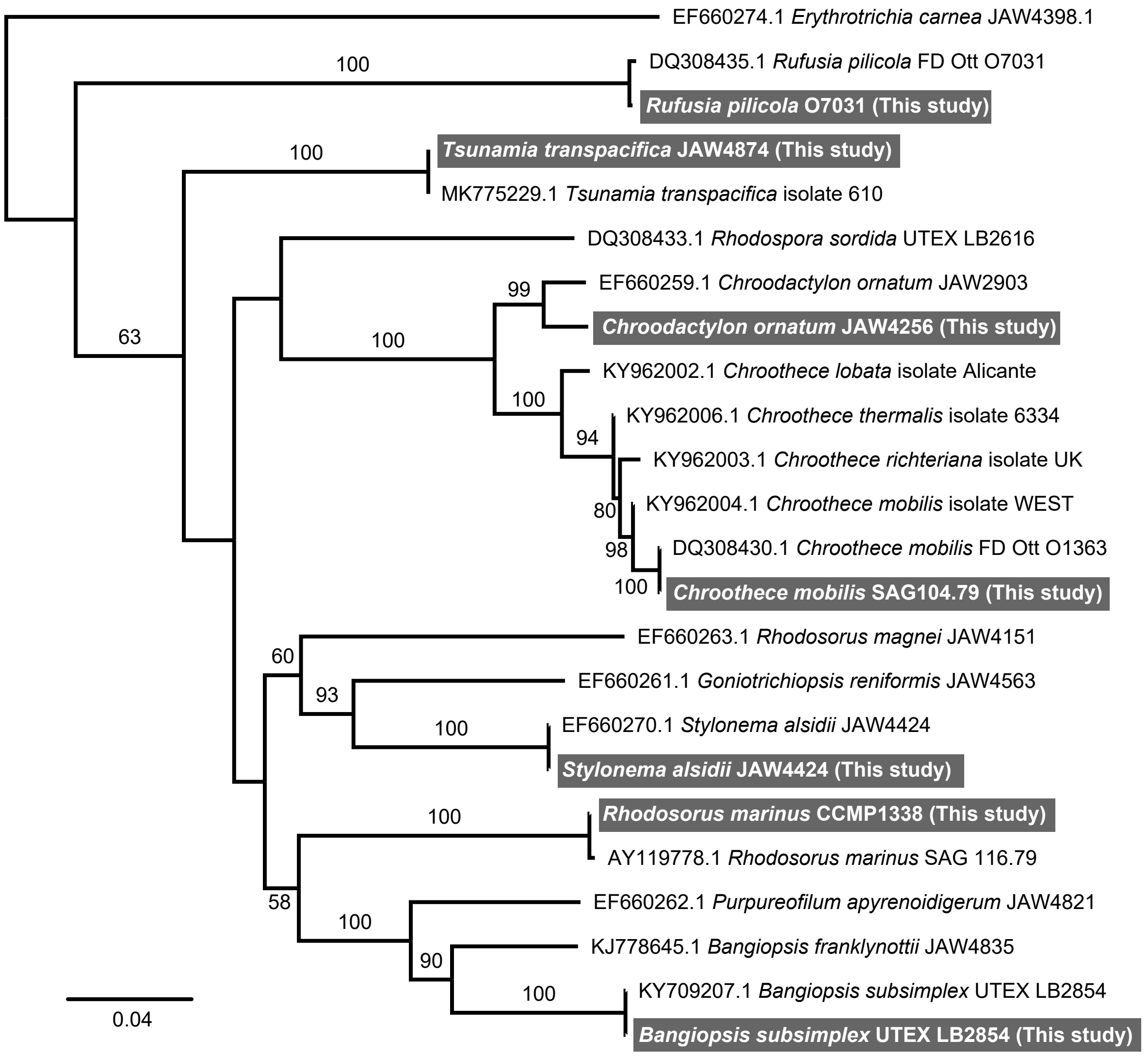


**Supplementary Fig. 1. Phylogeny of the Stylonematophyceae.** Maximum likelihood phylogenetic tree using the *rbcL* gene. SAG104.79 was labeled as *Chroothece richteriana*, however, the phylogenetic tree confirmed that SAG104.79, which we received, was *Chroothece mobilis*. Bootstrap values less than 50% are not shown. *Erythrotrichia carnea* (Compsopogonophyceae) was used as the outgroup root.

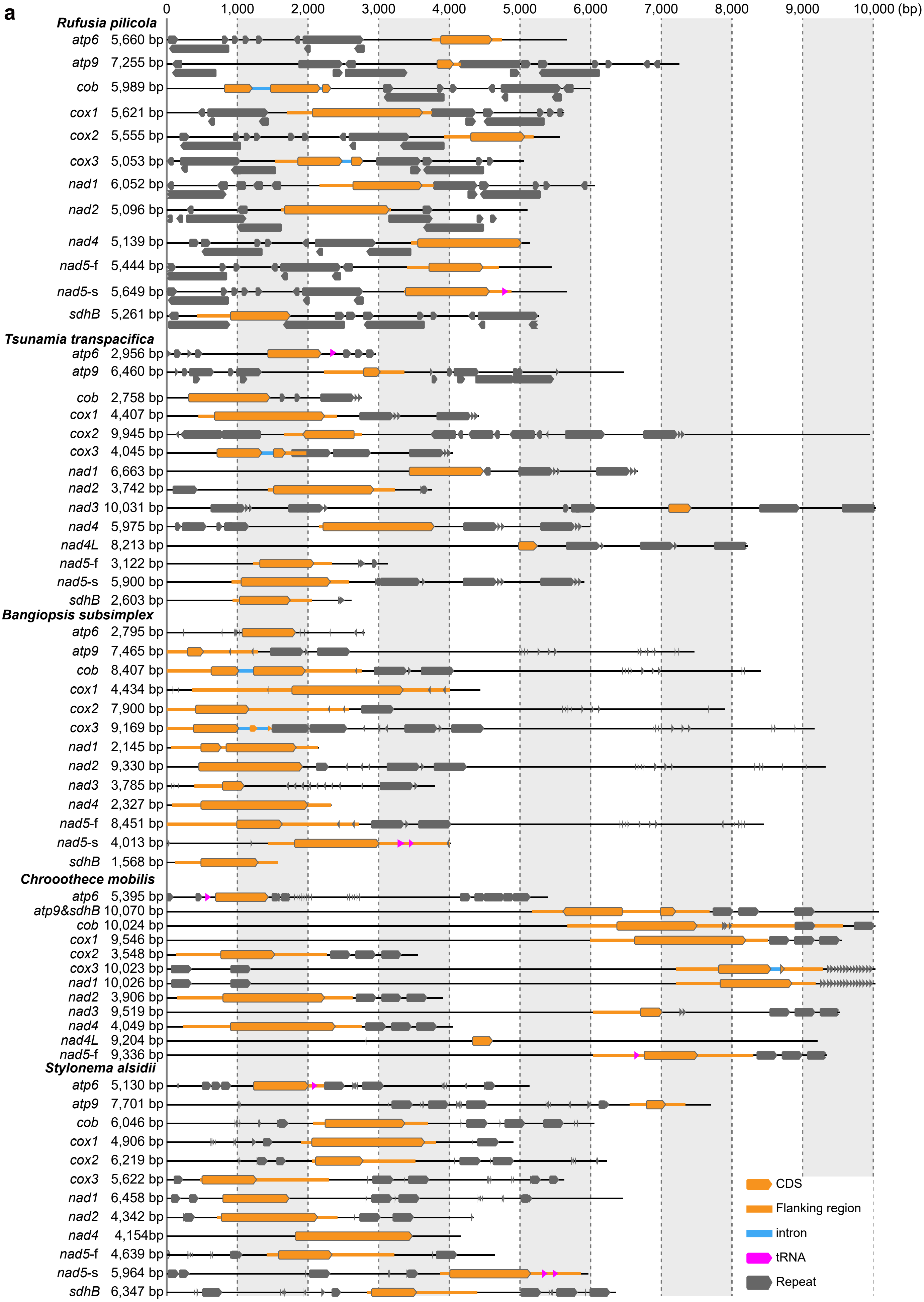
a
0
1,000
2,000
3,000
4,000
5,000
6,000
7,000
8,000
9,000
10,000 (bp)
Rufusia pilicola
atp6 5,660 bp
atp9 7,255 bp
cob 5,989 bp
cox1 5,621 bp
cox2 5,555 bp
cox3 5,053 bp
nad1 6,052 bp
nad2 5,096 bp
nad4 5,139 bp
nad5-f 5,444 bp
nad5-s 5,649 bp
sdhB 5,261 bp
Tsunamia transpacifica
atp6 2,956 bp
atp9 6,460 bp
cob 2,758 bp
cox1 4,407 bp
cox2 9,945 bp
cox3 4,045 bp
nad1 6,663 bp
nad2 3,742 bp
nad3 10,031 bp
nad4 5,975 bp
nad4L 8,213 bp
nad5-f 3,122 bp
nad5-s 5,900 bp
sdhB 2,603 bp
Bangiopsis subsimplex
atp6 2,795 bp
atp9 7,465 bp
cob 8,407 bp
cox1 4,434 bp
cox2 7,900 bp
cox3 9,169 bp
nad1 2,145 bp
nad2 9,330 bp
nad3 3,785 bp
nad4 2,327 bp
nad5-f 8,451 bp
nad5-s 4,013 bp
sdhB 1,568 bp
Chroothece mobilis
atp6 5,395 bp
atp9&sdhB 10,070 bp
cob 10,024 bp
cox1 9,546 bp
cox2 3,548 bp
cox3 10,023 bp
nad1 10,026 bp
nad2 3,906 bp
nad3 9,519 bp
nad4 4,049 bp
nad4L 9,204 bp
nad5-f 9,336 bp
Stylonema alsidii
atp6 5,130 bp
atp9 7,701 bp
cob 6,046 bp
cox1 4,906 bp
cox2 6,219 bp
cox3 5,622 bp
nad1 6,458 bp
nad2 4,342 bp
nad4 4,154bp
nad5-f 4,639 bp
nad5-s 5,964 bp
sdhB 6,347 bp
CDS
Flanking region
intron
tRNA
Repeat

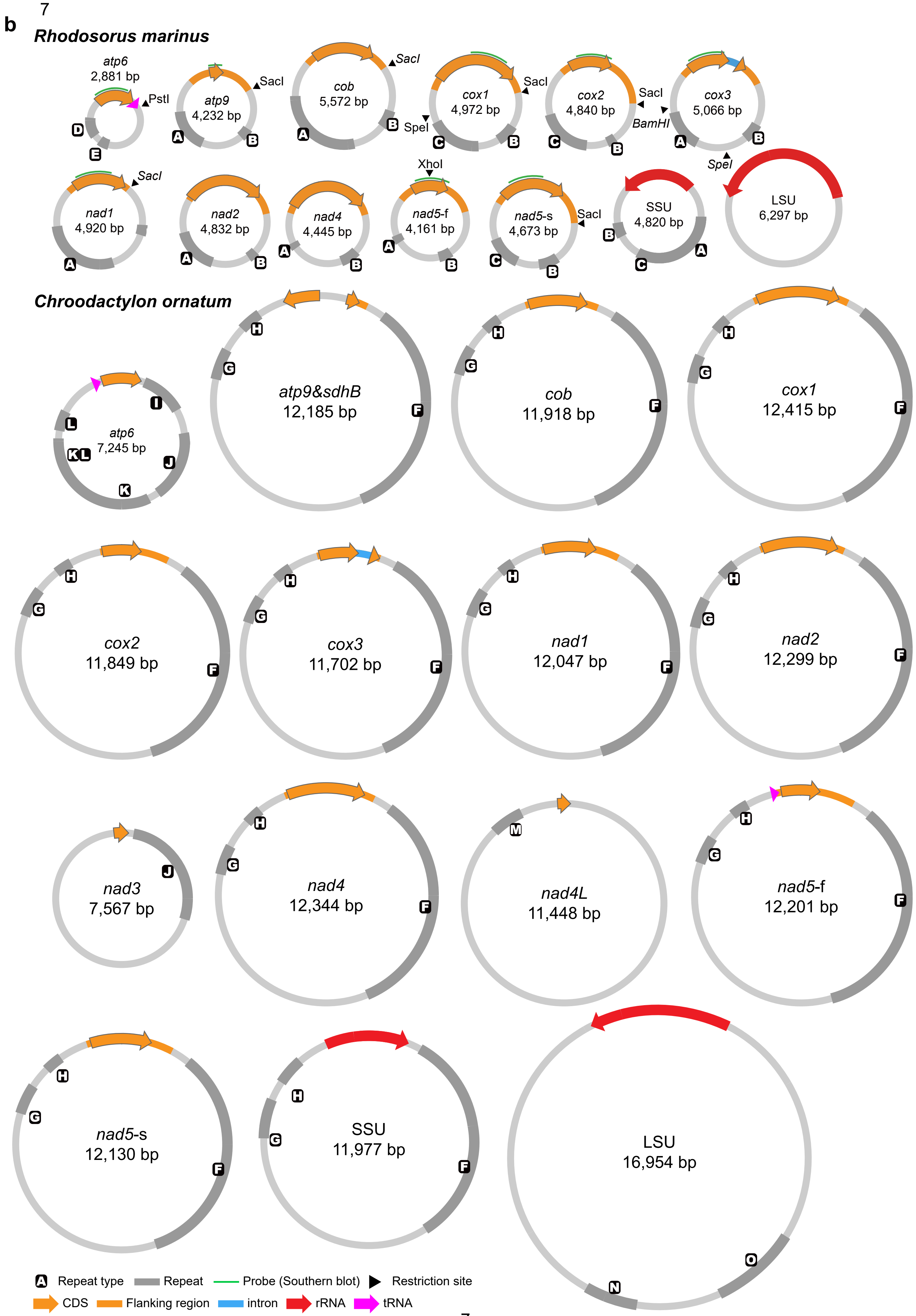
b
Rhodosorus marinus
atp6 2,881 bp
PstI
atp9 4,232 bp
SacI
cob 5,572 bp
SacI
cox1 4,972 bp
SacI
SpeI
cox2 4,840 bp
SacI
BamHI
cox3 5,066 bp
SpeI
nad1 4,920 bp
SacI
nad2 4,832 bp
nad4 4,445 bp
nad5-f 4,161 bp
XhoI
nad5-s 4,673 bp
SacI
SSU 4,820 bp
LSU 6,297 bp
Chroodactylon ornatum
atp6 7,245 bp
atp9&sdhB 12,185 bp
cob 11,918 bp
cox1 12,415 bp
cox2 11,849 bp
cox3 11,702 bp
nad1 12,047 bp
nad2 12,299 bp
nad3 7,567 bp
nad4 12,344 bp
nad4L 11,448 bp
nad5-f 12,201 bp
nad5-s 12,130 bp
SSU 11,977 bp
LSU 16,954 bp
A Repeat type
Repeat
Probe (Southern blot)
Restriction site
CDS
Flanking region
intron
rRNA
tRNA

**Supplementary Fig. 2. Visualization of mitogenomes of seven Stylonematophyceae species. (a)**

Partially retrieved mitogenome contigs of five species using short-read data. **(b)** Fully recovered mitogenomes (minicircle) of *R. marinus* (top) and *C. ornatum* (bottom) drawn to scale. As inferred from the type of repeat region (see Fig. 2b), five different constant regions reside in *atp6, nad3, nad4L, LSU* and the rest of the minicircles, respectively. Probes for the Southern blot analysis are shown with the greens line and restriction sites are marked with arrows with the name of restriction enzyme used.

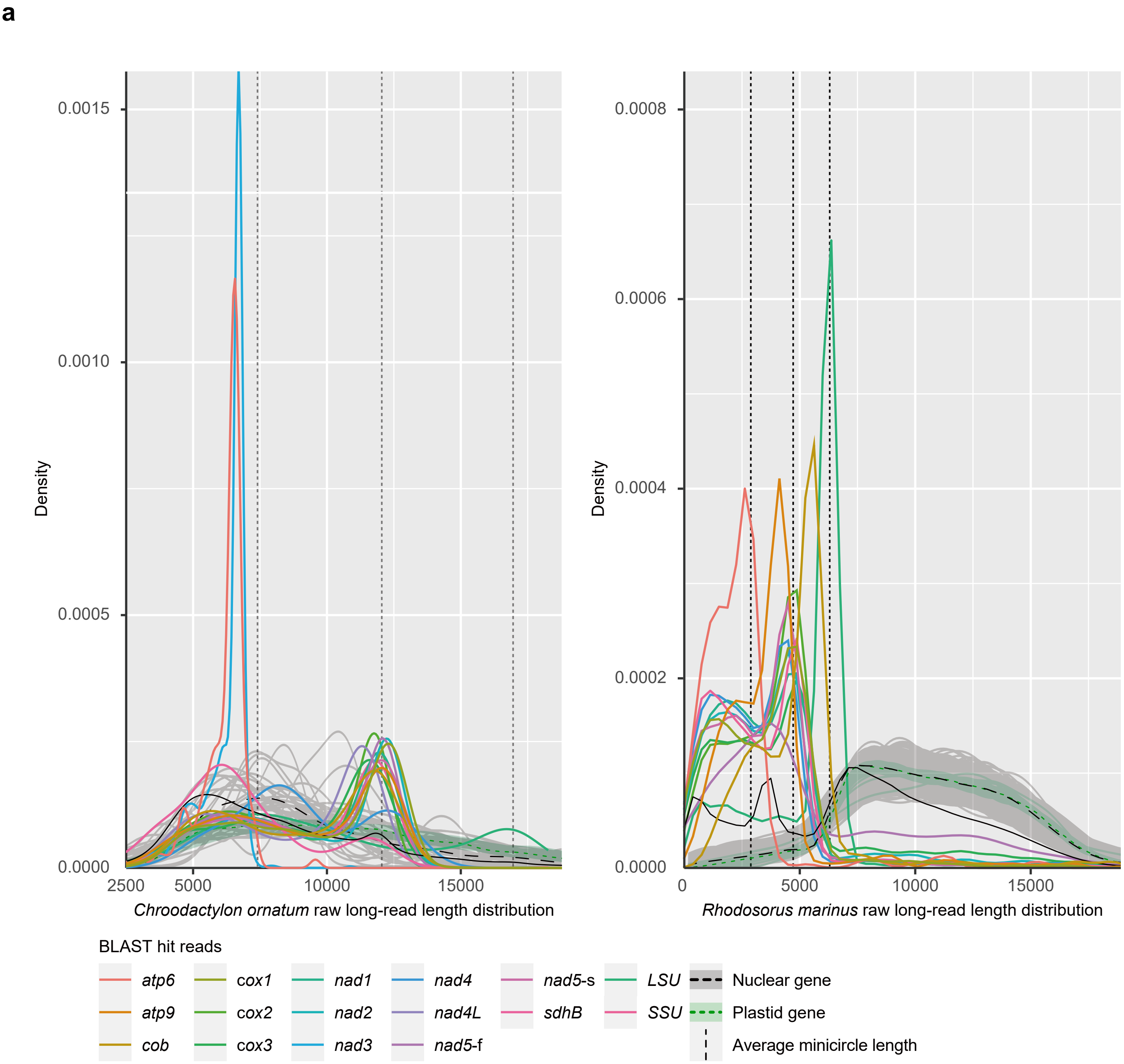
a
Density
0.0015
0.0010
0.0005
0.0000
2500
5000
10000
15000
Chroodactylon ornatum raw long-read length distribution
0.0008
0.0006
0.0004
0.0002
0
Rhodosorus marinus raw long-read length distribution
BLAST hit reads
atp6
atp9
cob
cox1
cox2
cox3
nad1
nad2
nad3
nad4
nad4L
nad5-f
nad5-s
sdhB
LSU
SSU
Nuclear gene
Plastid gene
Average minicircle length

**b**

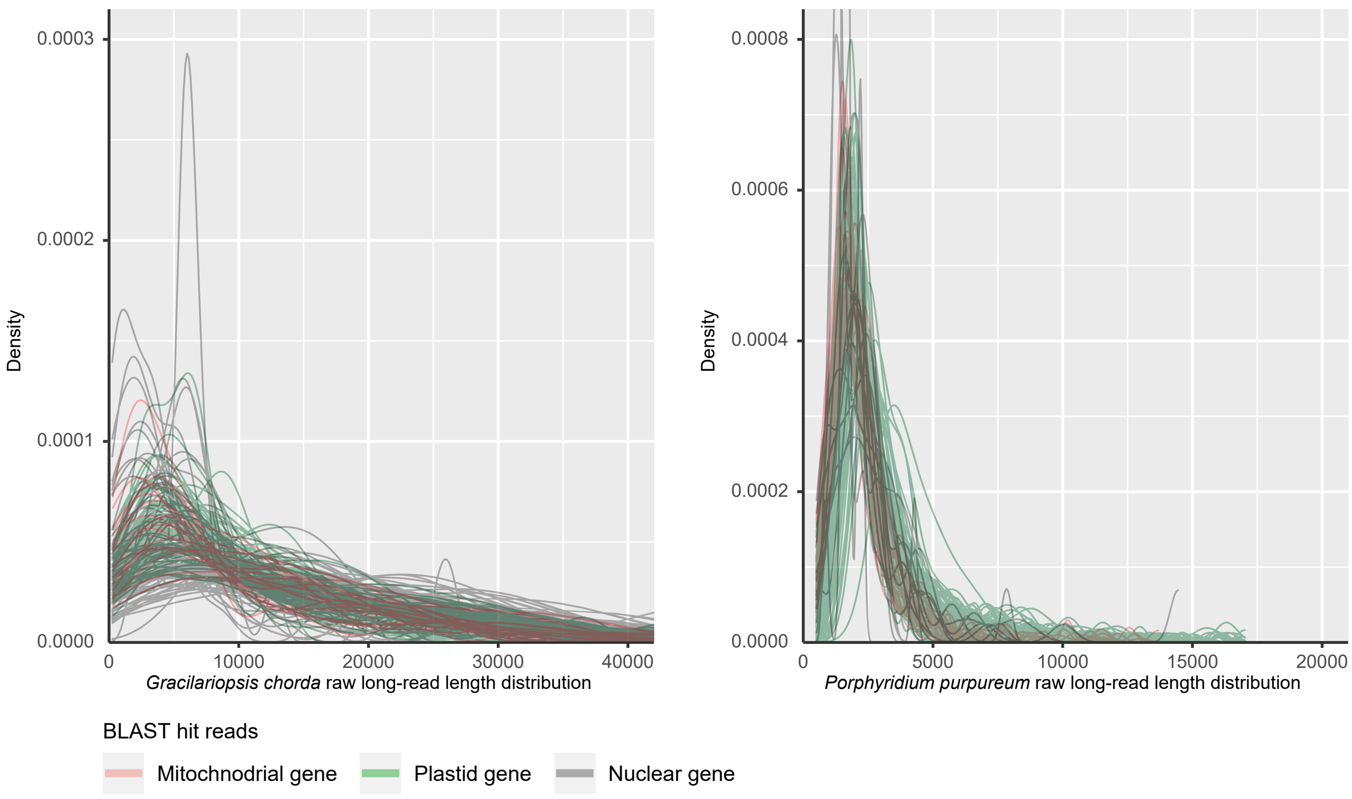

0.0003
0.0002
0.0001
0.0000
Density
0
10000
20000
30000
40000
*Gracilariopsis chorda* raw long-read length distribution
0.0008
0.0006
0.0004
0.0002
0.0000
Density
0
5000
10000
15000
20000
*Porphyridium purpureum* raw long-read length distribution
BLAST hit reads
Mitochnodrial gene
Plastid gene
Nuclear gene


**c**

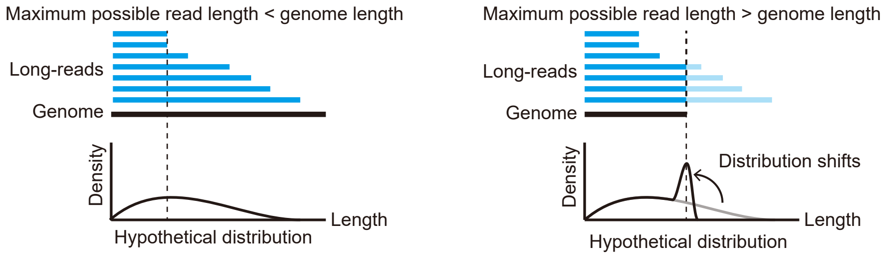

Maximum possible read length < genome length
Long-reads
Genome
Density
Length
Hypothetical distribution
Maximum possible read length > genome length
Long-reads
Genome
Distribution shifts
Density
Length
Hypothetical distribution

**Supplementary Fig. 3. Raw read length distribution density. (a)** Length distribution density plot of raw long-reads of *C. ornatum* (PacBio HiFi; left) and *R. marinus* (Oxford Nanopore; right). Dashed lines indicate average assembled length of minicircles classified into three types according to length. Black solid line indicates the average of all the other long-reads that were not annotated. Black and green dashed lines indicate the average of nuclear and plastid reads, respectively. Shaded regions represent all the trajectories of nuclear and plastid reads. In *C. ornatum,* the trajectory of nuclear reads almost completely overlaps with that of plastid reads. Colored solid lines indicate mitochondrial reads, labeled according to the gene they carry. Each read reaches at peak near the dashed line, implying that most reads are the size of the assembly (monomer; see Supplementary Note 1). **(b)** Length distribution density plot of raw long-reads of *G. chorda* (PacBio RSII; left) and *P. purpureum* (Oxford Nanopore; right) for comparison. *G. chorda* exhibit sharp peak near 2 kb, likely due to shearing and size selection during library preparation. In contrast, mitochondrial reads from *P. purpureum* show a wide and flat distribution. In either case, the distribution of mitochondrial reads is similar among plastid and nuclear data, unlike the case for Stylonematophyceae. **(c)** Illustrations on relationship between distribution and read length. If maximum possible read length is shorter than genome, length of reads will not be limited. Consequently, the distribution will be wide and flat. Peak location will vary data by data, depending on the most frequent read size generate in the whole procedure. On the other hand, if maximum possible read length is longer than genome, lengths of reads will be limited for the longer reads. This will produce a sharp peak near the size of the genome. Source data are provided as a Source Data file.

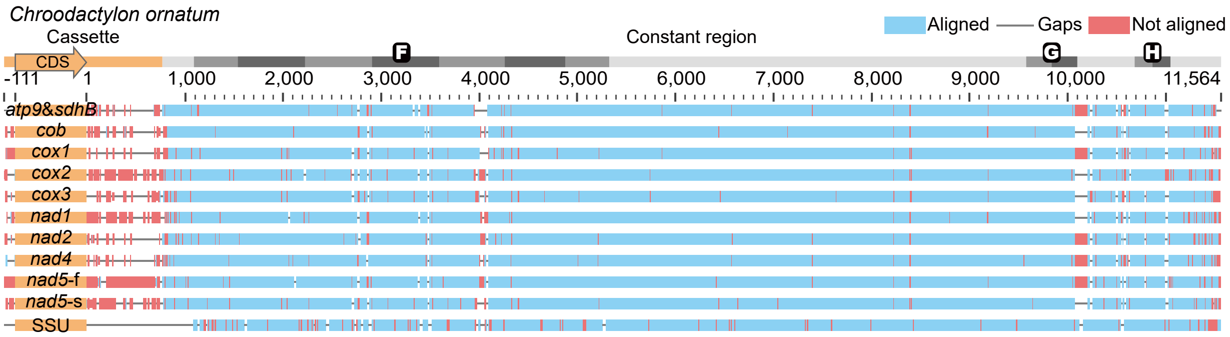


**Supplementary Fig. 4. Structure of minicircle and alignments of NCR of *C. ornatum*.** Structure of minicircles and alignments of the NCR of *C. ornatum*. The *atp6, nad3, nad4L* and *LSU* minicircles are not included, because these all have different NCRs. The blue and red boxes indicate aligned and unaligned regions, respectively. The grey line indicates gaps. The NCR in the cassette cannot be aligned, whereas that of the constant region aligns well. Dark gray regions indicate tandem repeat regions. Also see Supplementary Figure 6. Length and number of repeat units are indicated by alternating dark gray blocks. For example, two repeat units are present in repeat regions G and H. Note that red tick does not correctly correspond to scale, because it can arise from a single base mismatch.

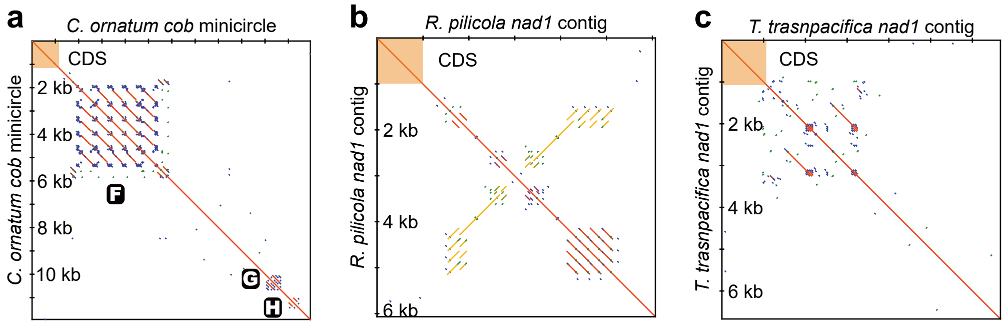


**Supplementary Fig. 5. Dot plots alignments of mitogenomes in the Stylonematophyceae.** Dot plot of self-alignment of (a) the *cob* minicircle in *C. ornatum* **(b)** the *nad1* contig in *R. pilicola,* and (c) the *nad1* contig in *T. transpacifica*. F, G, and H in (a) indicate repeat regions. Interestingly, only *R. pilicola* contains an inverted repeat in the minicircles. Short matches are colored blue or green whereas matches over 100 bp are colored red or yellow, depending on a direction.

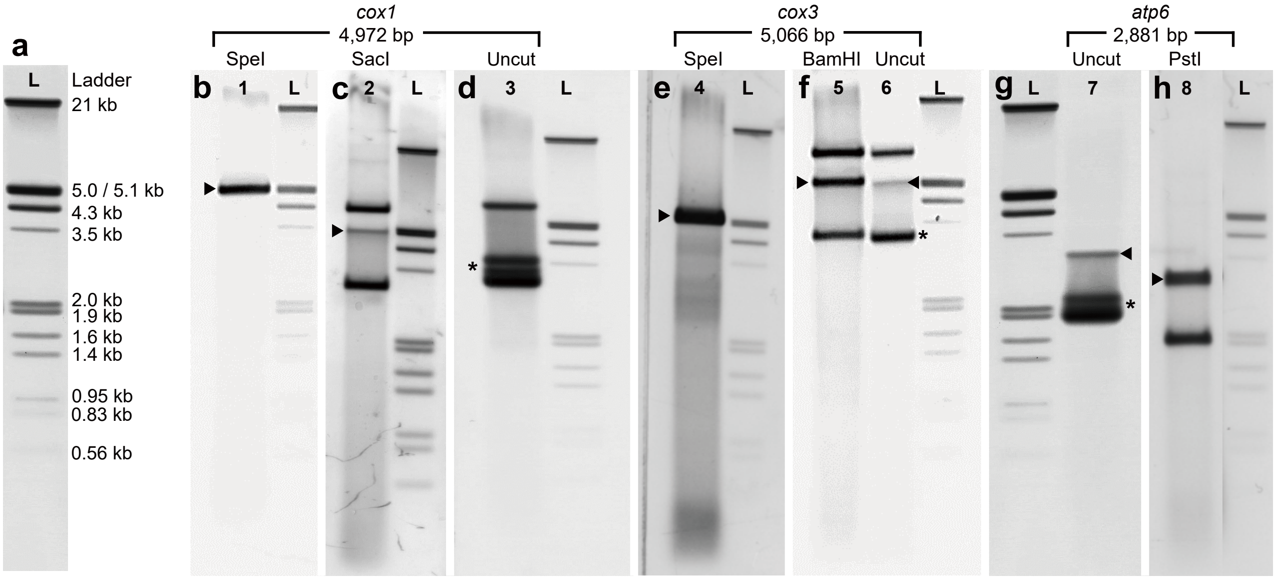


**Supplementary Fig. 6. Southern hybridization results. (a)** Ladders indicated with size. **(b-h)** Specific gene probes were used to study the gDNA of *R. marinus* and visualized on nylon membrane. Bands that represent linearized DNA are marked by a black arrow. Only with *Spe*I treatment, did a single band appear. When *Sac*I, *Pst*I, or *Bam*HI were used as restriction enzymes, multiple bands appeared. Additional bands may represent supercoiled and nicked DNAs. The multiple bands in lanes with digested DNAs are unexpected results. The changes in intensity of supercoiled DNA bands in uncut DNA (marked by asterisks) when compared to the linear DNA bands in digested DNA imply a circular topology for the former. See Supplementary Note 2. Membrane image in panel h were cropped to remove unnecessary lane. Ladder lanes that correspond to panel a are indicated with L. $n = 1$.

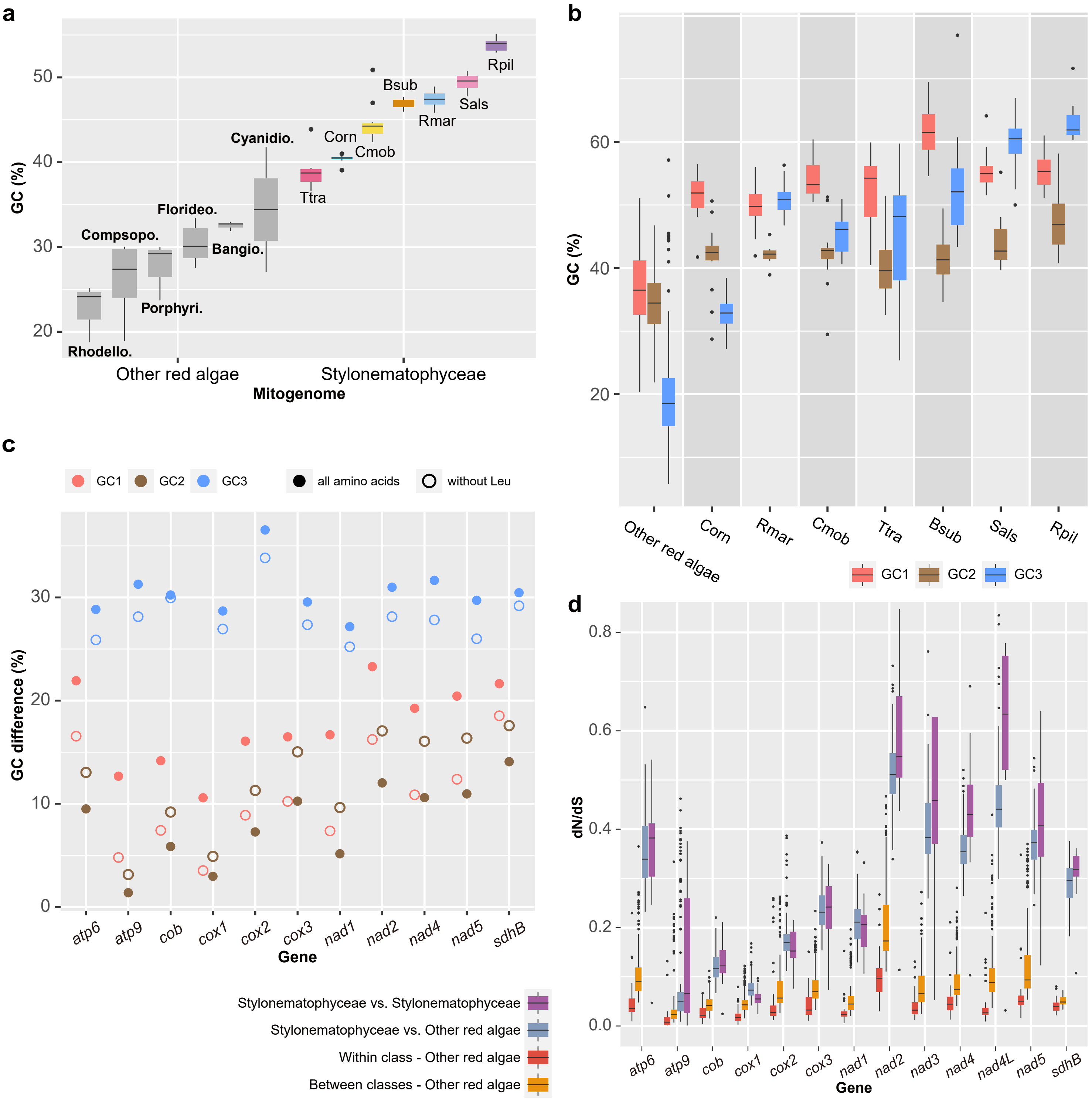
a
b
c
d
GC (%)
Rhodello.
Compsopo.
Porphyri.
Florideo.
Bangio.
Cyanidio.
Ttra
Corn
Cmob
Bsub
Rmar
Sals
Rpil
Other red algae
Stylonematophyceae
Mitogenome
Corn
Rmar
Cmob
Ttra
Bsub
Sals
Rpil
GC1
GC2
GC3
all amino acids
without Leu
GC difference (%)
atp6
atp9
cob
cox1
cox2
cox3
nad1
nad2
nad4
nad5
sdhB
Gene
dN/dS
nad3
nad4L
Stylonematophyceae vs. Stylonematophyceae
Stylonematophyceae vs. Other red algae
Within class - Other red algae
Between classes - Other red algae

**Supplementary Fig. 7. GC content per codon position and GC difference between Stylonematophyceae and other red algae.** See Fig. 4 for abbreviations. **(a)** Box plot indicating GC content of the entire mitogenome. Mitogenomes of the Stylonematophyceae show elevated GC content because both the CDS and the NCR share this trait. Bangio., $n$ = 3 species; Bsub, $n$ = 13 minicircles; Cmob, $n$ = 13 minicircles; Compsopo., $n$ = 5 species; Corn, $n$ = 13 minicircles; Cyanidio., $n$ = 2 species; Florideo., $n$ = 7 species; Porphyri., $n$ = 3 species; Rhodello., $n$ = 3 species; Rmar, $n$ = 11 minicircles; Rpil, $n$ = 11 minicircles; Sals, $n$ = 11 minicircles; Ttra, $n$ = 12 minicircles. **(b)** Box plot indicating GC1, GC2, and GC3 of 30 species. Only *C. ornatum* follows the pattern of other red algae (GC1>GC2>GC3), whereas GC3 is much higher in the other Stylonematophyceae. Bsub, $n$ = 13 CDSs; Cmob, $n$ = 13 CDSs; Corn, $n$ = 13 CDSs; Other red algae, $n$ = 300 CDSs over 23 species; Rmar, $n$ = 11 CDSs; Rpil, $n$ = 11 CDSs; Sals, $n$ = 11 CDSs; Ttra, $n$ = 12 CDSs. **(c)** GC content differences (between the Stylonematophyceae and the other red algae) in each CDS. GC3 difference is the highest in all CDSs. Without leucine (open circle), GC2 and GC1 differences become smaller, because six-fold degeneracy of leucine allows A→G transitions at the 1st codon position. **(d)** Dot plot indicating pairwise dN/dS values of four comparisons: 1) Stylonematophyceae vs. Stylonematophyceae, 2) Stylonematophyceae vs. other red algae, 3) Other red algae within the class, and 4) Other red algae between classes. Even within classes, Stylonematophyceae pairs have high dN/dS, comparable to Stylonematophyceae vs. other red algae pairs. In contrast, within-class pairs show lower dN/dS values compared to between-class pairs. However, it should be noted that although the dN/dS value generally indicates the direction of selection, here we used the dN/dS as a measure of sequence divergence. Source data are provided as a Source Data file.
Stylonematophyceae vs. Stylonematophyceae, $n$ = 42 (*atp6, atp9, cob, cox1, cox2, cox3, nad1, nad2,* and *nad4*), $n$ = 12 (*nad3*), $n$ = 20 (*nad4L*), $n$ = 30 (*nad5* and *sdhB*); Stylonematophyceae vs. Other red algae, $n$ = 322 (*atp6, atp9, cob, cox1, cox2, nad1, nad2,* and *nad4*), $n$ = 320 (*cox3*), $n$ = 184 (*nad3*), $n$ = 230 (*nad4L*), $n$ = 276 (*nad5*), $n$ = 132 (*sdhB*); Within class – Other red algae, $n$ = 82 (*atp6, atp9, coxb, cox1, cox2, cox3, nad1, nad3, nad4, nad4L* and *nad5*), $n$ = 80 (*nad2*), $n$ = 24 (*sdhB*); Between class – Other red algae, $n$ = 424 (*atp6, atp9, cob, cox1, cox2, cox3, nad1, nad3, nad4, nad4L* and *nad5*), $n$ = 408 (*nad2*), $n$ = 62 (*sdhB*). Boxes in panel a, b, and d include the 25$^{th}$ (Q1) to 75$^{th}$ (Q3) percentiles of the data with the median value in a thick line. Upper and lower whiskers indicate values within1.5 times interquartile range (Q3 – Q1) above 75$^{th}$ percentile and below 25$^{th}$ percentile, respectively.

| Protein / Nucleotide | | Stylo. | | | | | | | Cyanidio. | | Porphyri. | | | Compsopo. | | | | | Rhodello. | | | Bangio. | | | Florideo. | | | | | | |
|---|---|---|---|---|---|---|---|---|---|---|---|---|---|---|---|---|---|---|---|---|---|---|---|---|---|---|---|---|---|---|---|
| | | Rpil | Ttra | Bsub | Cmob | Corn | Sals | Rmar | Cmer | Gsul | Ppur | Paer | Ecox | Ccae | Ssub | Pven | Mery | Pkor | Dgri | Ncya | Rvio | Pumb | Pyez | Bfus | Hrub | Ppal | Apli | Coff | Gele | Ccri | Gcho |
| Stylo. | Rpil | | 57.1 | 58.7 | 60.8 | 59.7 | 53.1 | 54.5 | 56.5 | 51.3 | 50.3 | 53.9 | 54.4 | 57.1 | 56.3 | 56.8 | 57.4 | 56.7 | 55.6 | 54.2 | 54.5 | 58 | 58 | 55.6 | 55.3 | 57.7 | 58.6 | 57.7 | 58 | 58.2 | 58.4 |
| | Ttra | 44.7 | | 64 | 65.6 | 64.2 | 55.8 | 57 | 58.1 | 54.7 | 50.6 | 55.6 | 55.6 | 57.3 | 57.2 | 57.3 | 56.9 | 57.6 | 56.8 | 55.5 | 55.8 | 58.3 | 58.4 | 55.7 | 55.1 | 58.3 | 58.9 | 58.3 | 59.3 | 58.9 | 59 |
| | Bsub | 47 | 48.2 | | 67.6 | 66.5 | 57.7 | 58 | 58.6 | 54.6 | 52.2 | 57.2 | 56.3 | 58.7 | 59 | 58.3 | 58.4 | 59.1 | 57.3 | 55.9 | 56.4 | 59.3 | 59.5 | 56.9 | 57.3 | 60 | 60.6 | 59.4 | 60.1 | 60.5 | 60.3 |
| | Cmob | 46.4 | 49.1 | 53 | | 92.5 | 61.6 | 62.1 | 60 | 55.6 | 53.7 | 58.1 | 57.2 | 58.7 | 59.9 | 59.1 | 59.6 | 59.9 | 58.7 | 57.6 | 57.2 | 61.2 | 60.9 | 58 | 58.7 | 60.4 | 61.6 | 60.7 | 61.6 | 61.5 | 60.9 |
| | Corn | 45 | 48.7 | 52.4 | 79.1 | | 59.8 | 61.2 | 58.1 | 53.7 | 52.2 | 56.7 | 55.9 | 57 | 57.7 | 57.5 | 57.9 | 58 | 56.7 | 56.1 | 56.2 | 59.4 | 59 | 56.6 | 57.4 | 59.2 | 60 | 59.2 | 59.9 | 60.1 | 59.5 |
| | Sals | 42.2 | 40.5 | 45.8 | 49.1 | 46.6 | | 56.2 | 54.4 | 50.4 | 49.6 | 52.6 | 52.1 | 53.9 | 53.6 | 53.7 | 53.6 | 53.6 | 52.9 | 51.8 | 52.4 | 54.3 | 54.2 | 51.5 | 52.3 | 54.3 | 54.6 | 54.4 | 54.7 | 54.6 | 54.3 |
| | Rmar | 41.5 | 40.2 | 43.7 | 44.5 | 43.1 | 45.3 | | 55 | 51.4 | 49.8 | 53.8 | 52.8 | 54.5 | 54.4 | 54.2 | 54.2 | 54.6 | 53.1 | 52.2 | 53 | 54.7 | 54.6 | 52.3 | 53.2 | 55.4 | 55.6 | 55.5 | 55.4 | 55.5 | 55.7 |
| Cyanidio. | Cmer | 42.8 | 46 | 45.2 | 47.3 | 47.3 | 40.3 | 41.6 | | 60.9 | 62.7 | 66.9 | 68.4 | 74.2 | 74.3 | 73.6 | 74.8 | 75.1 | 71.2 | 70.5 | 70.5 | 76.1 | 76 | 72.9 | 71.2 | 75.9 | 77.2 | 76.2 | 76.5 | 77.3 | 76.8 |
| | Gsul | 39.8 | 45.1 | 41 | 40.9 | 40.3 | 36 | 40.3 | 48.3 | | 54.3 | 57.2 | 58.4 | 60.2 | 60.5 | 59.6 | 60.6 | 60.6 | 59.2 | 58 | 57.7 | 61.4 | 61.3 | 58.8 | 58.4 | 61.5 | 62.2 | 61.2 | 62.5 | 61.9 | 62.4 |
| Porphyri. | Ppur | 39.2 | 41.6 | 42.2 | 43.8 | 43.9 | 38.2 | 39.4 | 53.2 | 42.8 | | 62.9 | 64.6 | 62.4 | 62.5 | 62.6 | 62.6 | 62.9 | 62.6 | 61.9 | 59.5 | 63.1 | 62.9 | 60.4 | 60.3 | 63.6 | 63.9 | 63.7 | 63.7 | 64.4 | 63.7 |
| | Paer | 41.3 | 44.7 | 44 | 45.8 | 46.8 | 39.6 | 41.1 | 59.3 | 45 | 54.8 | | 69.2 | 67.1 | 67.1 | 67.4 | 67.8 | 68.1 | 65.3 | 64.9 | 63.4 | 68.3 | 68 | 65 | 64.9 | 67.8 | 67.9 | 67.8 | 68.3 | 69 | 69 |
| | Ecox | 40.9 | 44.6 | 43.5 | 46.4 | 46.9 | 39.5 | 41.2 | 60.3 | 45.9 | 55.7 | 62.3 | | 69.8 | 69.8 | 69.9 | 69.3 | 70.2 | 68.3 | 68 | 66.2 | 70 | 70.2 | 67.5 | 66.9 | 70.4 | 70.4 | 70.4 | 70.7 | 71.4 | 70.8 |
| Compsopo. | Ccae | 42.6 | 45.5 | 44.7 | 46.3 | 46.6 | 41 | 42 | 63 | 47.3 | 52.5 | 58.5 | 60.4 | | 81.1 | 85.7 | 81.2 | 81.9 | 73.2 | 72 | 71.4 | 76.9 | 77.4 | 74.3 | 71.6 | 76.6 | 77.2 | 76.4 | 77.2 | 76.8 | 77.2 |
| | Ssub | 42.8 | 45.6 | 45.5 | 47 | 47.3 | 41 | 41.7 | 63.8 | 46.9 | 53.8 | 59.2 | 60.9 | 68.1 | | 80 | 84.7 | 90.8 | 73.5 | 71.6 | 71.4 | 77.9 | 78 | 74.8 | 72.2 | 77.7 | 78.7 | 77.4 | 78.5 | 78.9 | 78.8 |
| | Pven | 42.7 | 46.2 | 44.7 | 47.4 | 48 | 39.9 | 41.4 | 64.5 | 47.1 | 54.3 | 60.9 | 62.5 | 74.2 | 69.5 | | 80.1 | 80.6 | 73.4 | 71.9 | 71.1 | 76 | 76.1 | 73.5 | 71.6 | 76.4 | 76.6 | 76.1 | 76.8 | 76.7 | 76.6 |
| | Mery | 42.8 | 46.3 | 45.5 | 47.7 | 47.8 | 40.6 | 42.5 | 64.1 | 47.4 | 53.8 | 60.5 | 62.2 | 68.3 | 72.1 | 70.3 | | 86 | 72.8 | 72.1 | 71.7 | 77.9 | 77.9 | 75.1 | 72.6 | 77.7 | 79.1 | 77.4 | 78.6 | 79.1 | 78.7 |
| | Pkor | 43.1 | 46 | 46 | 47.1 | 47.7 | 41 | 42 | 63.3 | 47.7 | 53.4 | 59.5 | 60.9 | 68.2 | 76.4 | 69 | 73 | | 74 | 72.1 | 72.2 | 78.8 | 78.7 | 75.8 | 72.4 | 78.3 | 79.8 | 78.4 | 79.3 | 79.3 | 79.2 |
| Rhodello. | Dgri | 42.1 | 45.9 | 44.2 | 46.9 | 47.6 | 40.2 | 41.2 | 62.8 | 46.9 | 54.1 | 59.3 | 62 | 63.5 | 63.8 | 66.1 | 64.7 | 64 | | 80 | 71 | 73.5 | 73.1 | 70.4 | 69.3 | 74.1 | 74.8 | 74.1 | 74.9 | 75.1 | 74.4 |
| | Ncya | 40.8 | 44.9 | 43.5 | 46.4 | 47 | 39.3 | 40.4 | 62.3 | 45.8 | 53.8 | 59.7 | 62 | 62.5 | 62.5 | 65.3 | 63.9 | 63.6 | 71.1 | | 70.6 | 72.4 | 72 | 69.7 | 68.6 | 72.7 | 73 | 72.5 | 73 | 73.3 | 73 |
| | Rvio | 41.3 | 45.4 | 44.1 | 46.4 | 47.2 | 40.3 | 40.8 | 61.2 | 44.9 | 51.5 | 56.7 | 58.5 | 60.5 | 61.8 | 62.3 | 62.4 | 61.4 | 62.1 | 62.1 | | 71.9 | 72.2 | 69.6 | 69.8 | 73 | 73.2 | 72.3 | 73.3 | 73.5 | 73.2 |
| Bangio. | Pumb | 43.7 | 46.4 | 45.8 | 47.8 | 48 | 41.4 | 43.1 | 63.8 | 48.2 | 53.5 | 58.8 | 60.2 | 64.1 | 65.1 | 65.2 | 65.7 | 65.1 | 63.1 | 62.1 | 61.5 | | 95 | 91.4 | 76.6 | 83 | 84.4 | 82.3 | 82.5 | 83.4 | 83.5 |
| | Pyez | 44.3 | 46.9 | 45.7 | 47.4 | 47.9 | 41.7 | 43 | 63.5 | 47.8 | 53.4 | 59 | 59.9 | 63.5 | 64.7 | 64.9 | 65.9 | 65.2 | 62.9 | 61.8 | 60.9 | 79.4 | | 91.5 | 76.5 | 82.9 | 84.1 | 81.8 | 82.7 | 83.1 | 83.1 |
| | Bfus | 42.1 | 44.5 | 43.3 | 45.1 | 45.3 | 39.5 | 41.4 | 61 | 46.2 | 51.3 | 56.1 | 57.4 | 61.4 | 62 | 62.3 | 63.1 | 62.8 | 60.6 | 59.8 | 58.8 | 76.9 | 76.5 | | 73.5 | 79.8 | 80.9 | 78.9 | 79.3 | 80 | 79.9 |
| Florideo. | Hrub | 42.1 | 44.6 | 44.6 | 46.3 | 46.8 | 40.7 | 41.7 | 60.9 | 45.6 | 51.5 | 56.3 | 58 | 61 | 61.3 | 62.3 | 62 | 61.1 | 60.6 | 60 | 59 | 64 | 63.6 | 61.3 | | 77.2 | 78.6 | 77.2 | 76.9 | 77.9 | 77.5 |
| | Ppal | 44.2 | 46.9 | 46.4 | 48.1 | 48.5 | 41 | 42 | 63.9 | 48.2 | 53.8 | 59.2 | 60.7 | 64.2 | 65.7 | 65.1 | 66.4 | 66.2 | 63.5 | 62.3 | 61.3 | 68.7 | 67.9 | 65.4 | 64.6 | | 88.6 | 85.5 | 85.3 | 86.7 | 86 |
| | Apli | 44.2 | 46.3 | 46.8 | 48.3 | 48.1 | 42.5 | 43.6 | 63.6 | 47.9 | 52.9 | 58.1 | 59.7 | 63.2 | 64.6 | 63.7 | 65.5 | 65.5 | 62.9 | 61.3 | 60.8 | 68.8 | 67.8 | 65.9 | 64.5 | 71.5 | | 87.3 | 87.9 | 89.7 | 89.2 |
| | Coff | 44.1 | 46 | 46.2 | 47.5 | 48.1 | 41.9 | 43.1 | 64.3 | 47.8 | 54 | 59.5 | 60.8 | 64.5 | 65.5 | 65.5 | 66.6 | 65.8 | 64.1 | 62.7 | 61.6 | 68.1 | 67.3 | 64.8 | 64.4 | 70.4 | 71.4 | | 85.1 | 86 | 85.3 |
| | Gele | 43.9 | 46.9 | 46.8 | 48.3 | 48.7 | 41.9 | 43 | 64.9 | 48.6 | 54.5 | 59.9 | 61.5 | 64.6 | 65.4 | 66 | 67 | 65.6 | 64.4 | 62.9 | 61.7 | 68.1 | 67.9 | 65.1 | 64 | 70.9 | 71.3 | 70 | | 87.9 | 87.8 |
| | Ccri | 43.9 | 46.5 | 46.4 | 48.3 | 48.7 | 42 | 42.9 | 65.1 | 47.8 | 54.7 | 60.9 | 62.5 | 65 | 66.6 | 66.3 | 67.7 | 66.6 | 65.2 | 64 | 62.5 | 69.1 | 68.9 | 66 | 65.6 | 72.1 | 74.2 | 71.9 | 73.8 | | 88.6 |
| | Gcho | 43.7 | 47.1 | 46.3 | 47.3 | 48 | 41 | 42.9 | 65.7 | 49 | 54.5 | 61 | 61.9 | 65.4 | 66.8 | 66.9 | 67.7 | 67.5 | 65.6 | 64 | 62.4 | 69.7 | 69.4 | 66.2 | 65.6 | 72.1 | 73 | 71.4 | 73.7 | 74.9 | |

36.0 Nucleotide identity 79.4

49.6 Protein similarity 95.0

**Supplementary Fig. 8. Pairwise sequence distances of 30 species.** See Fig. 4 for abbreviations. Pairwise nucleotide identity and amino acid similarity of 30 species is shown in the heatmap. The percentage values indicate nucleotide identity and amino acid similarity. Stylonematophyceae generally shows a low identity/similarity when compared to the other red algae.

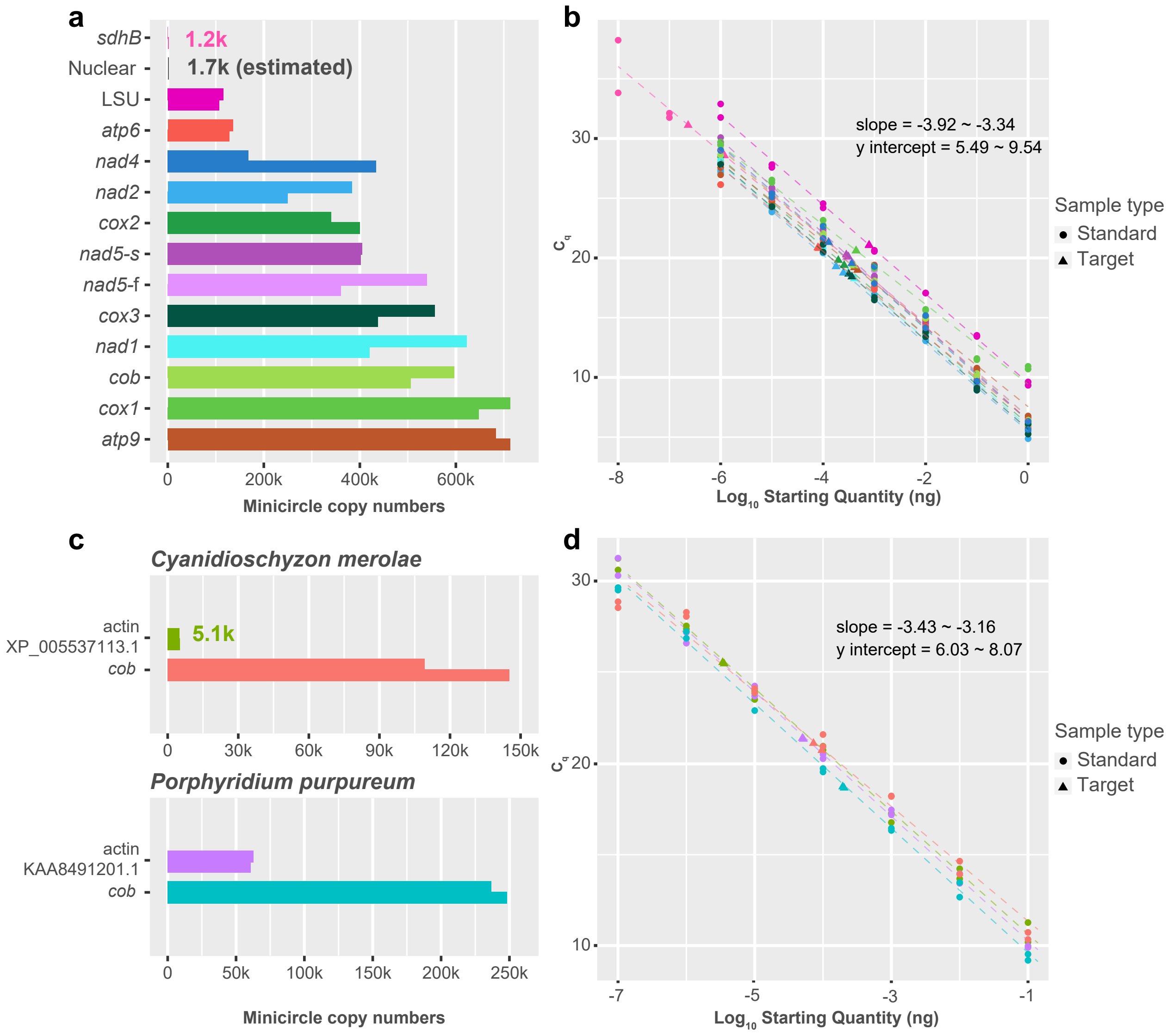


**Supplementary Fig. 9. Copy numbers calculated from qPCR and standard curve.** See Supplementary Data 3 for more information. **(a)** Copy numbers of each gene minicircles are calculated based on qPCR result. Two bars on each row represent two replicates. Copy number of nuclear genomes was estimated based on assembled nuclear genome size. The copy number of *sdhB*, which is an EGT-derived gene, roughly corresponds with the estimated copy number in the nuclear genome. Compared to *sdhB*, minicircles have, on average, more than a 357-fold higher copy number. **(b)** Standard curves plotted with target samples (0.06 ng of extracted gDNA). Colors represent genes on the left panel. Dashed lines indicate standard curves. **(c)** Copy numbers of the mitogenome (*cob*) and nuclear genome (actin) of *C. merolae* (top) and *P. purpureum* (bottom) are calculated based on qPCR results. The two bars on each row represent two replicates. *C. merolae* shows a 25.1-fold difference in copy number and *P. purpureum* shows 3.93-fold difference in copy number. **(d)** Standard curves plotted with target samples. Colors represent genes on the left panel. Dashed lines indicate standard curves. Source data are provided as a Source Data file.

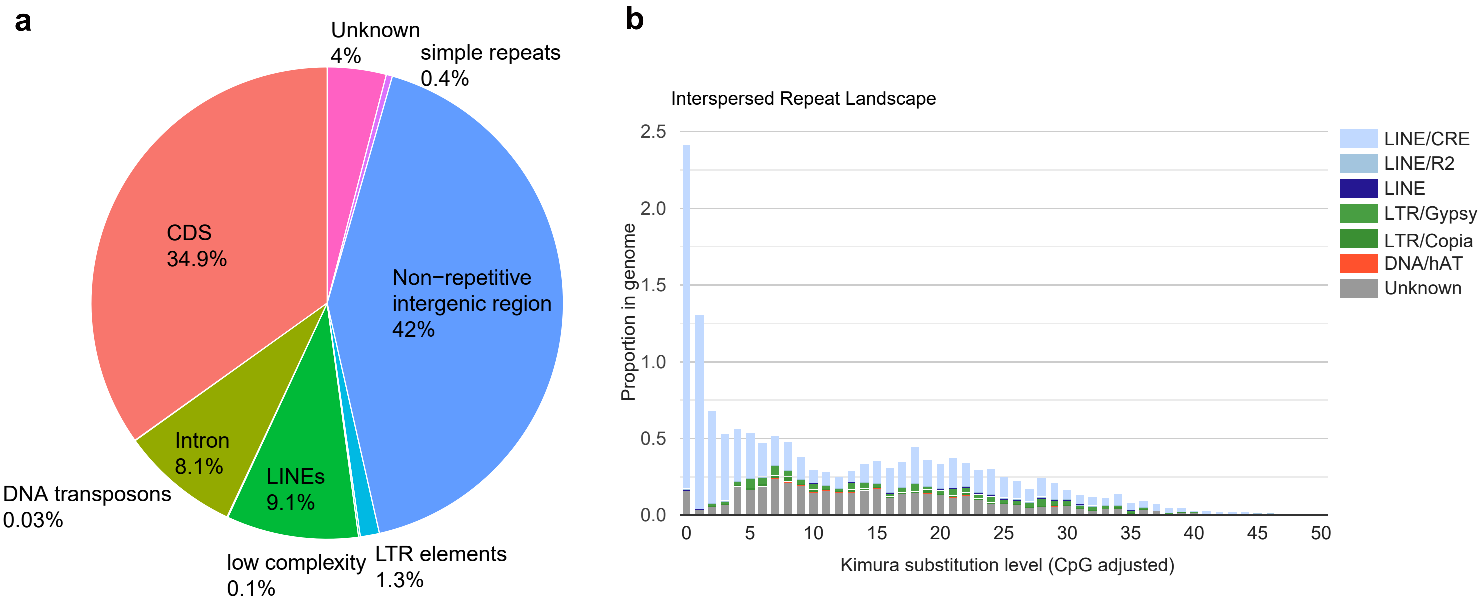


**Supplementary Fig. 10. Description of assembled nuclear genome of R. marinus. (a)** Composition of the *R. marinus* nuclear genome. Repeat region occupy ~ 15% of the genome. **(b)** Kimura distance of repetitive elements. Majority of repeats show low kimura substitution level. Source data are provided as a Source Data file.

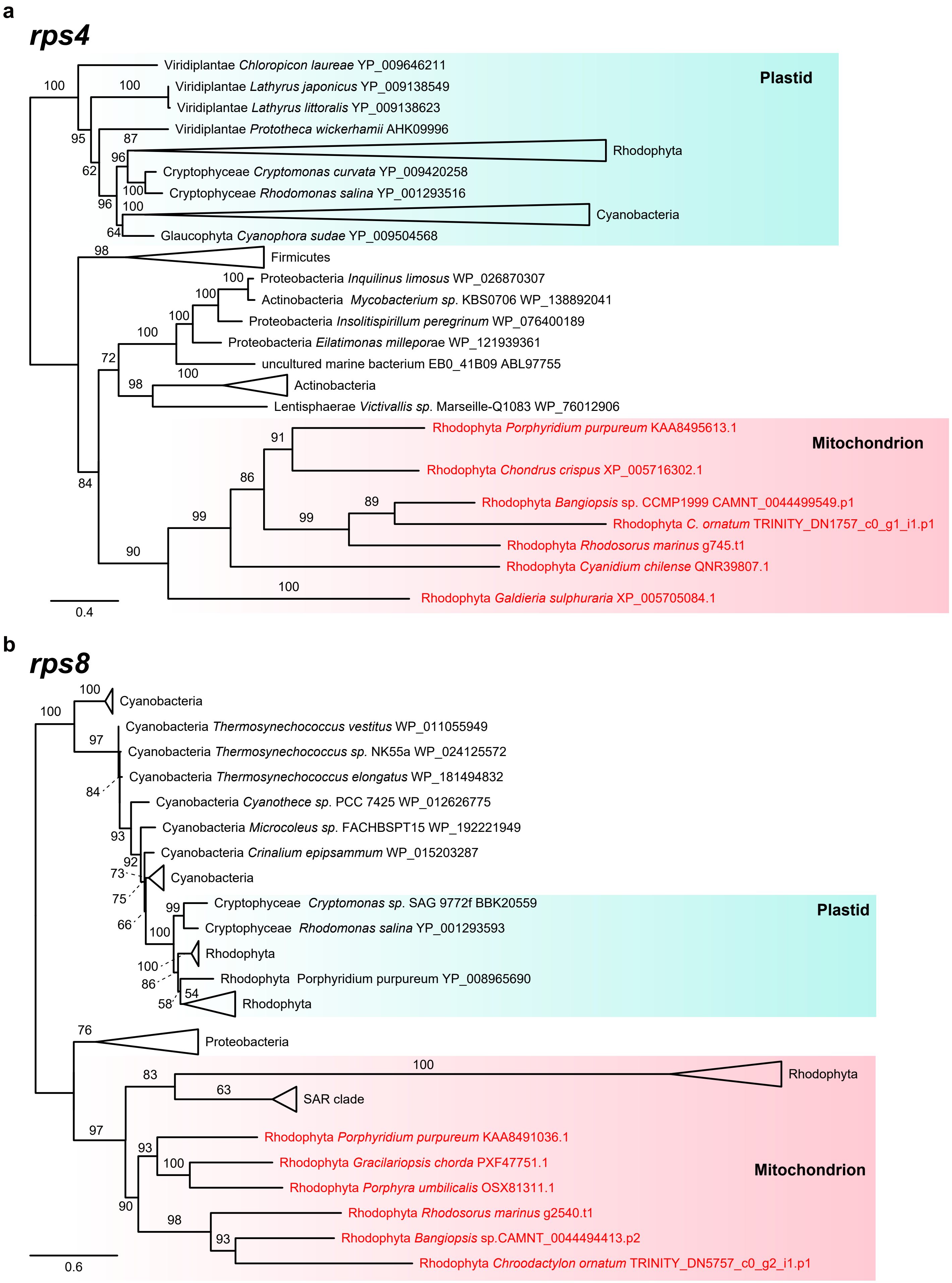
a
rps4
Plastid
Viridiplantae Chloropicon laureae YP_009646211
Viridiplantae Lathyrus japonicus YP_009138549
Viridiplantae Lathyrus littoralis YP_009138623
Viridiplantae Prototheca wickerhamii AHK09996
Rhodophyta
Cryptophyceae Cryptomonas curvata YP_009420258
Cryptophyceae Rhodomonas salina YP_001293516
Cyanobacteria
Glaucophyta Cyanophora sudae YP_009504568
Firmicutes
Proteobacteria Inquilinus limosus WP_026870307
Actinobacteria Mycobacterium sp. KBS0706 WP_138892041
Proteobacteria Insolitispirillum peregrinum WP_076400189
Proteobacteria Eilatimonas milleporae WP_121939361
uncultured marine bacterium EB0_41B09 ABL97755
Actinobacteria
Lentisphaerae Victivallis sp. Marseille-Q1083 WP_76012906
Mitochondrion
Rhodophyta Porphyridium purpureum KAA8495613.1
Rhodophyta Chondrus crispus XP_005716302.1
Rhodophyta Bangiopsis sp. CCMP1999 CAMNT_0044499549.p1
Rhodophyta C. ornatum TRINITY_DN1757_c0_g1_i1.p1
Rhodophyta Rhodosorus marinus g745.t1
Rhodophyta Cyanidium chilense QNR39807.1
Rhodophyta Galdieria sulphuraria XP_005705084.1
0.4
b
rps8
Cyanobacteria
Cyanobacteria Thermosynechococcus vestitus WP_011055949
Cyanobacteria Thermosynechococcus sp. NK55a WP_024125572
Cyanobacteria Thermosynechococcus elongatus WP_181494832
Cyanobacteria Cyanothece sp. PCC 7425 WP_012626775
Cyanobacteria Microcoleus sp. FACHBSPT15 WP_192221949
Cyanobacteria Crinalium epipsammum WP_015203287
Cyanobacteria
Plastid
Cryptophyceae Cryptomonas sp. SAG 9772f BBK20559
Cryptophyceae Rhodomonas salina YP_001293593
Rhodophyta
Rhodophyta Porphyridium purpureum YP_008965690
Rhodophyta
Proteobacteria
Rhodophyta
SAR clade
Mitochondrion
Rhodophyta Porphyridium purpureum KAA8491036.1
Rhodophyta Gracilariopsis chorda PXF47751.1
Rhodophyta Porphyra umbilicalis OSX81311.1
Rhodophyta Rhodosorus marinus g2540.t1
Rhodophyta Bangiopsis sp.CAMNT_0044494413.p2
Rhodophyta Chroodactylon ornatum TRINITY_DN5757_c0_g2_i1.p1
0.6

c

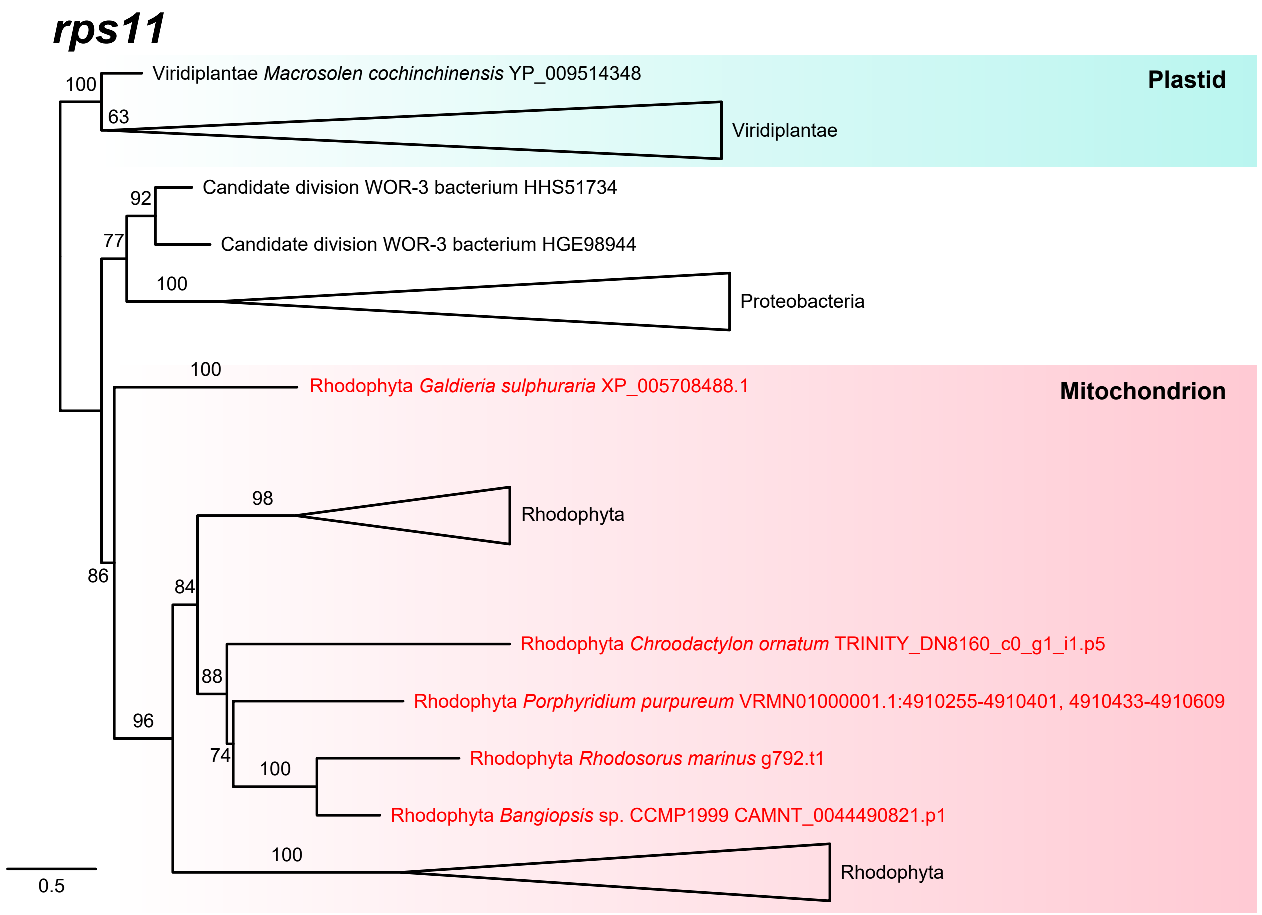
rps11
Plastid
Viridiplantae Macrosolen cochinchinensis YP_009514348
Viridiplantae
Candidate division WOR-3 bacterium HHS51734
Candidate division WOR-3 bacterium HGE98944
Proteobacteria
Mitochondrion
Rhodophyta Galdieria sulphuraria XP_005708488.1
Rhodophyta
Rhodophyta Chroodactylon ornatum TRINITY_DN8160_c0_g1_i1.p5
Rhodophyta Porphyridium purpureum VRMN01000001.1:4910255-4910401, 4910433-4910609
Rhodophyta Rhodosorus marinus g792.t1
Rhodophyta Bangiopsis sp. CCMP1999 CAMNT_0044490821.p1
Rhodophyta
0.5

d

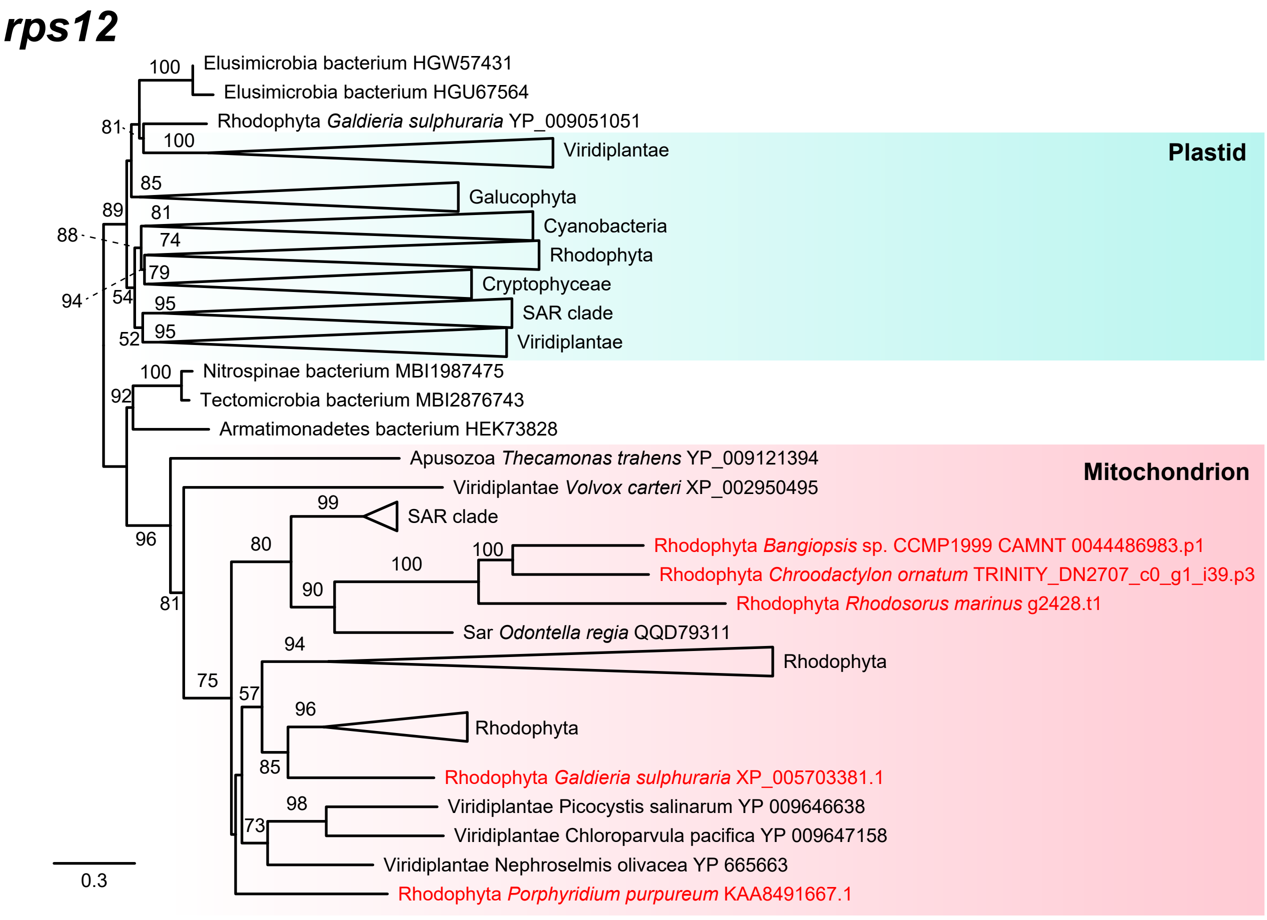
rps12
Elusimicrobia bacterium HGW57431
Elusimicrobia bacterium HGU67564
Rhodophyta Galdieria sulphuraria YP_009051051
Viridiplantae
Plastid
Galucophyta
Cyanobacteria
Rhodophyta
Cryptophyceae
SAR clade
Viridiplantae
Nitrospinae bacterium MBI1987475
Tectomicrobia bacterium MBI2876743
Armatimonadetes bacterium HEK73828
Apusozoa Thecamonas trahens YP_009121394
Viridiplantae Volvox carteri XP_002950495
Mitochondrion
SAR clade
Rhodophyta Bangiopsis sp. CCMP1999 CAMNT 0044486983.p1
Rhodophyta Chroodactylon ornatum TRINITY_DN2707_c0_g1_i39.p3
Rhodophyta Rhodosorus marinus g2428.t1
Sar Odontella regia QQD79311
Rhodophyta
Rhodophyta
Rhodophyta Galdieria sulphuraria XP_005703381.1
Viridiplantae Picocystis salinarum YP 009646638
Viridiplantae Chloroparvula pacifica YP 009647158
Viridiplantae Nephroselmis olivacea YP 665663
Rhodophyta Porphyridium purpureum KAA8491667.1
0.3

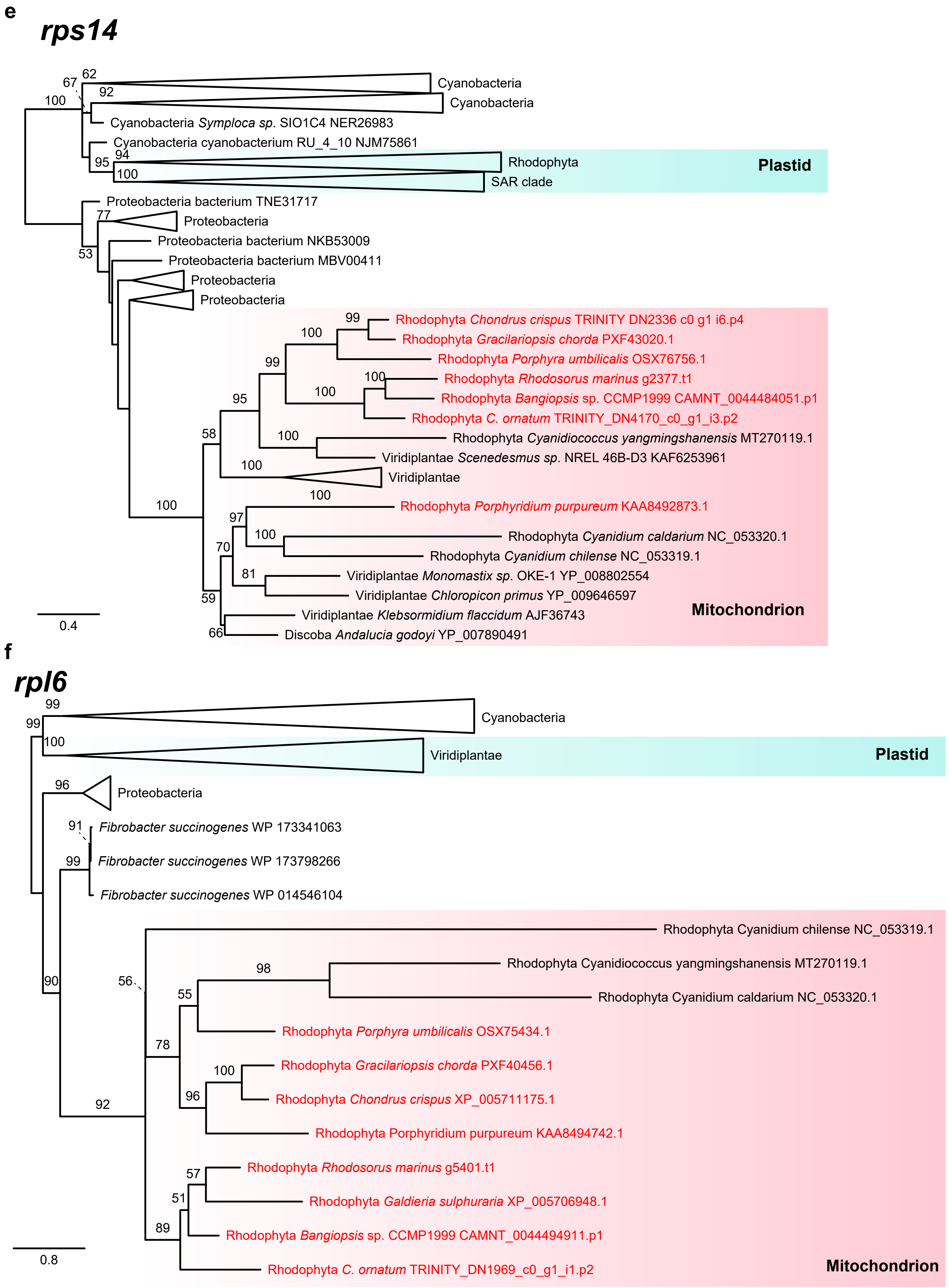
e
rps14
Cyanobacteria
Cyanobacteria
Cyanobacteria Symploca sp. SIO1C4 NER26983
Cyanobacteria cyanobacterium RU_4_10 NJM75861
Rhodophyta
SAR clade
Plastid
Proteobacteria bacterium TNE31717
Proteobacteria
Proteobacteria bacterium NKB53009
Proteobacteria bacterium MBV00411
Proteobacteria
Proteobacteria
Rhodophyta Chondrus crispus TRINITY DN2336 c0 g1 i6.p4
Rhodophyta Gracilariopsis chorda PXF43020.1
Rhodophyta Porphyra umbilicalis OSX76756.1
Rhodophyta Rhodosorus marinus g2377.t1
Rhodophyta Bangiopsis sp. CCMP1999 CAMNT_0044484051.p1
Rhodophyta C. ornatum TRINITY_DN4170_c0_g1_i3.p2
Rhodophyta Cyanidiococcus yangmingshanensis MT270119.1
Viridiplantae Scenedesmus sp. NREL 46B-D3 KAF6253961
Viridiplantae
Rhodophyta Porphyridium purpureum KAA8492873.1
Rhodophyta Cyanidium caldarium NC_053320.1
Rhodophyta Cyanidium chilense NC_053319.1
Viridiplantae Monomastix sp. OKE-1 YP_008802554
Viridiplantae Chloropicon primus YP_009646597
Viridiplantae Klebsormidium flaccidum AJF36743
Discoba Andalucia godoyi YP_007890491
Mitochondrion
0.4
f
rpl6
Cyanobacteria
Viridiplantae
Plastid
Proteobacteria
Fibrobacter succinogenes WP 173341063
Fibrobacter succinogenes WP 173798266
Fibrobacter succinogenes WP 014546104
Rhodophyta Cyanidium chilense NC_053319.1
Rhodophyta Cyanidiococcus yangmingshanensis MT270119.1
Rhodophyta Cyanidium caldarium NC_053320.1
Rhodophyta Porphyra umbilicalis OSX75434.1
Rhodophyta Gracilariopsis chorda PXF40456.1
Rhodophyta Chondrus crispus XP_005711175.1
Rhodophyta Porphyridium purpureum KAA8494742.1
Rhodophyta Rhodosorus marinus g5401.t1
Rhodophyta Galdieria sulphuraria XP_005706948.1
Rhodophyta Bangiopsis sp. CCMP1999 CAMNT_0044494911.p1
Rhodophyta C. ornatum TRINITY_DN1969_c0_g1_i1.p2
Mitochondrion
0.8

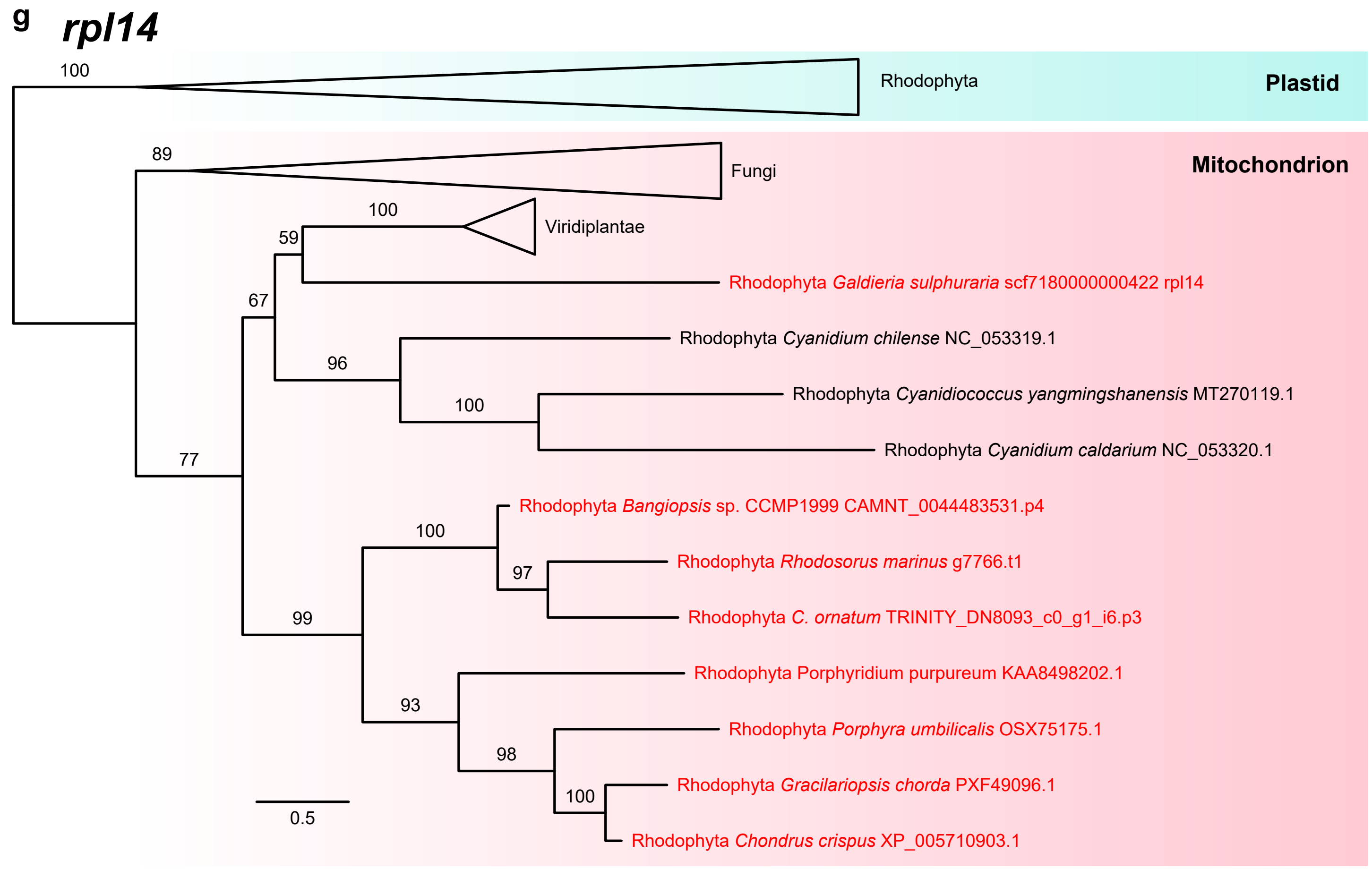
g
rpl14
100
Rhodophyta
Plastid
89
Fungi
Mitochondrion
100
59
Viridiplantae
Rhodophyta Galdieria sulphuraria scf7180000000422 rpl14
67
Rhodophyta Cyanidium chilense NC_053319.1
96
Rhodophyta Cyanidiococcus yangmingshanensis MT270119.1
100
Rhodophyta Cyanidium caldarium NC_053320.1
77
Rhodophyta Bangiopsis sp. CCMP1999 CAMNT_0044483531.p4
100
Rhodophyta Rhodosorus marinus g7766.t1
97
Rhodophyta C. ornatum TRINITY_DN8093_c0_g1_i6.p3
99
Rhodophyta Porphyridium purpureum KAA8498202.1
93
Rhodophyta Porphyra umbilicalis OSX75175.1
98
Rhodophyta Gracilariopsis chorda PXF49096.1
100
Rhodophyta Chondrus crispus XP_005710903.1
0.5

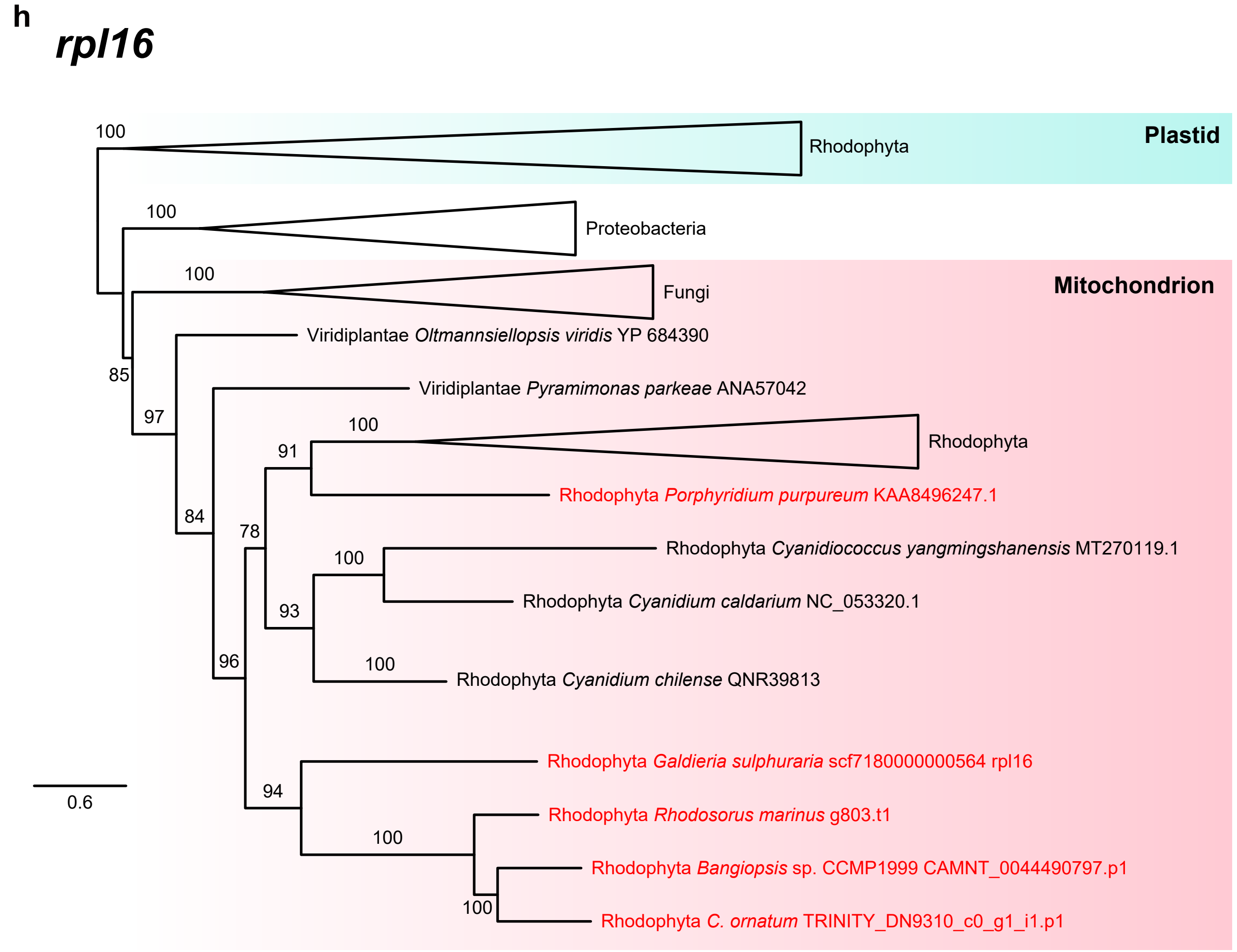
h
rpl16
100
Rhodophyta
Plastid
100
Proteobacteria
100
Fungi
Mitochondrion
Viridiplantae Oltmannsiellopsis viridis YP 684390
85
Viridiplantae Pyramimonas parkeae ANA57042
97
100
Rhodophyta
91
Rhodophyta Porphyridium purpureum KAA8496247.1
84
78
Rhodophyta Cyanidiococcus yangmingshanensis MT270119.1
100
Rhodophyta Cyanidium caldarium NC_053320.1
93
100
Rhodophyta Cyanidium chilense QNR39813
96
Rhodophyta Galdieria sulphuraria scf7180000000564 rpl16
94
Rhodophyta Rhodosorus marinus g803.t1
100
Rhodophyta Bangiopsis sp. CCMP1999 CAMNT_0044490797.p1
100
Rhodophyta C. ornatum TRINITY_DN9310_c0_g1_i1.p1
0.6

i

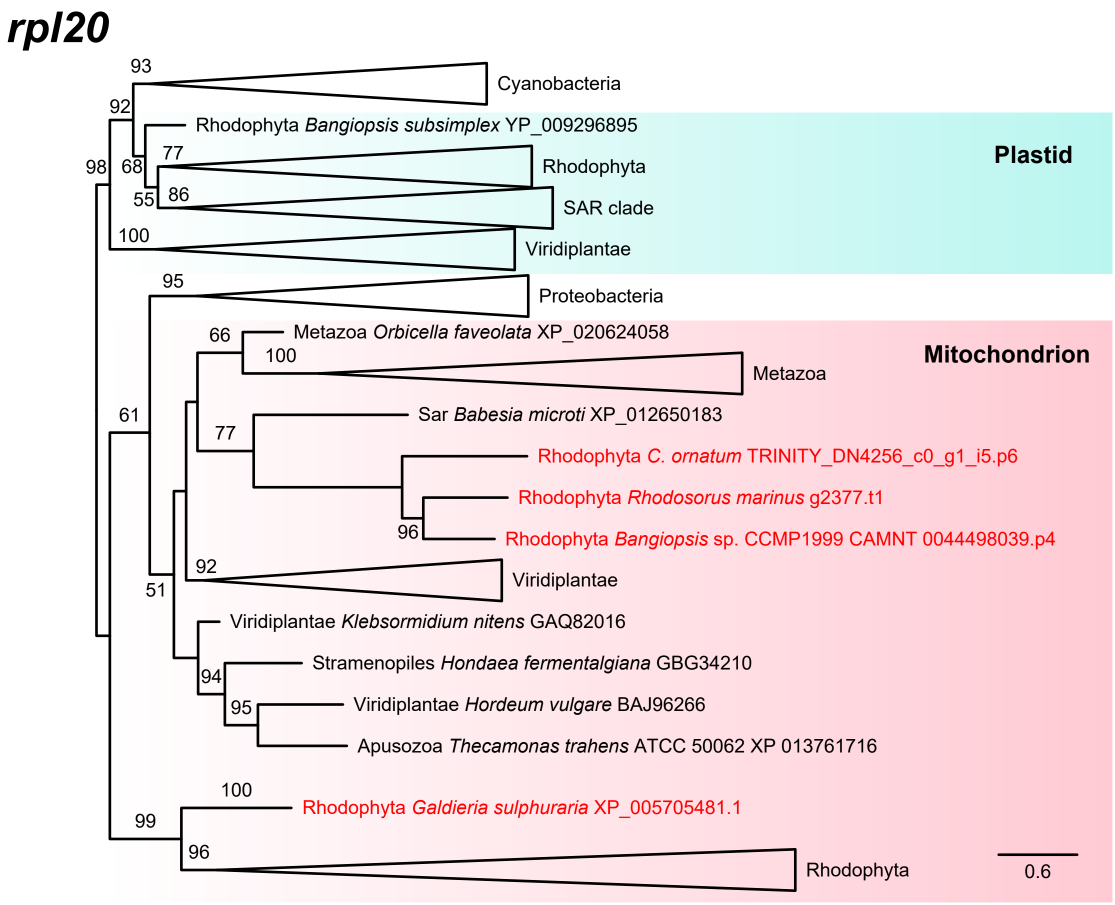
rpl20
Cyanobacteria
Rhodophyta Bangiopsis subsimplex YP_009296895
Rhodophyta
SAR clade
Viridiplantae
Plastid
Proteobacteria
Metazoa Orbicella faveolata XP_020624058
Metazoa
Mitochondrion
Sar Babesia microti XP_012650183
Rhodophyta C. ornatum TRINITY_DN4256_c0_g1_i5.p6
Rhodophyta Rhodosorus marinus g2377.t1
Rhodophyta Bangiopsis sp. CCMP1999 CAMNT 0044498039.p4
Viridiplantae
Viridiplantae Klebsormidium nitens GAQ82016
Stramenopiles Hondaea fermentalgiana GBG34210
Viridiplantae Hordeum vulgare BAJ96266
Apusozoa Thecamonas trahens ATCC 50062 XP 013761716
Rhodophyta Galdieria sulphuraria XP_005705481.1
Rhodophyta
0.6

j

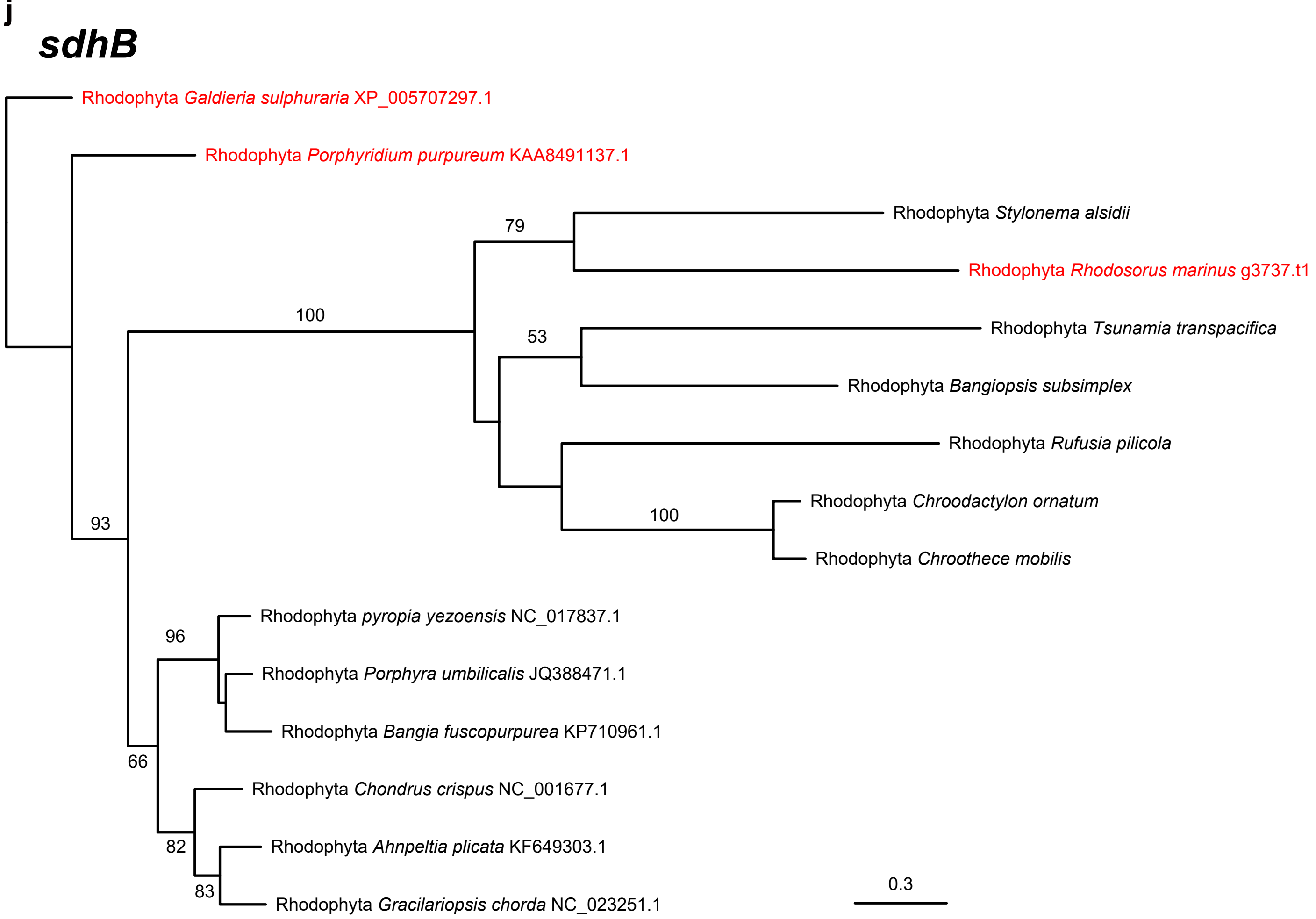
sdhB
Rhodophyta Galdieria sulphuraria XP_005707297.1
Rhodophyta Porphyridium purpureum KAA8491137.1
Rhodophyta Stylonema alsidii
Rhodophyta Rhodosorus marinus g3737.t1
Rhodophyta Tsunamia transpacifica
Rhodophyta Bangiopsis subsimplex
Rhodophyta Rufusia pilicola
Rhodophyta Chroodactylon ornatum
Rhodophyta Chroothece mobilis
Rhodophyta pyropia yezoensis NC_017837.1
Rhodophyta Porphyra umbilicalis JQ388471.1
Rhodophyta Bangia fuscopurpurea KP710961.1
Rhodophyta Chondrus crispus NC_001677.1
Rhodophyta Ahnpeltia plicata KF649303.1
Rhodophyta Gracilariopsis chorda NC_023251.1
0.3

**Supplementary Fig. 11. Phylogenetic tree of EGT-derived genes.** Phylogenetic tree of **(a)** *rps4*, **(b)** *rps8*, **(c)** *rps11*, **(d)** *rps12*, **(e)** *rps14*, **(f)** *rpl6*, **(g)** *rpl14*, **(h)** *rpl16*, **(i)** *rpl20*, **(j)** *sdhB.* Only EGT-derived genes from red algae are colored in red. Bootstrap value under 50 are not shown.

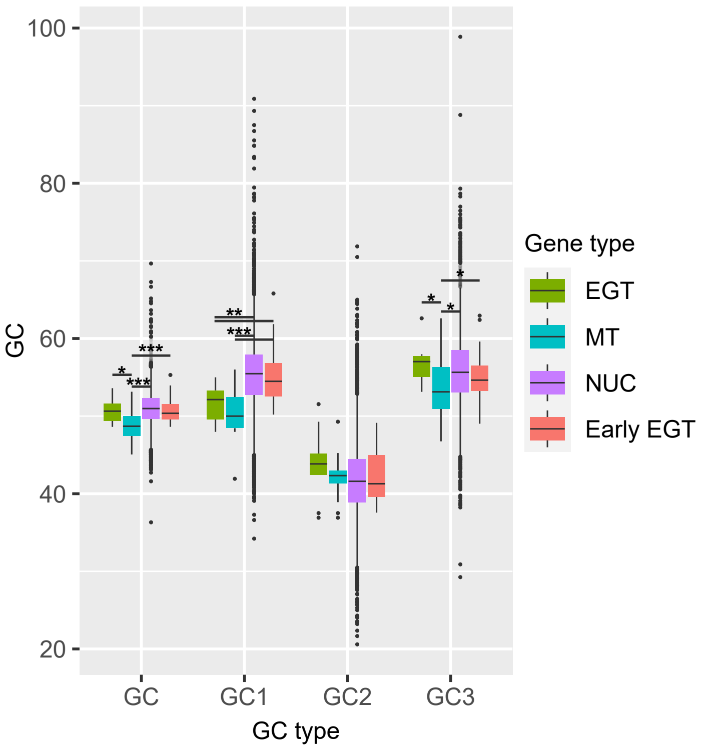


**Supplementary Fig. 12. GC content of EGT, mitochondrial, nuclear, and early EGT genes.** Overall GC content shows that mitochondrial genes are different from other genes. This pattern is also shown in GC3. In contrast, GC1 reveals difference between EGT-derived and NUC+Early EGT-derived genes. Simultaneously, the difference between EGT-derived and mitochondrial genes becomes insignificant. GC2 does not show any significant difference. The $p$-values (Wilcoxon rank sum test with continuity correction, two-sided) are indicated by stars, * $p<0.05$, ** $p<0.01$, *** $p<0.001$. GC, $p = 0.021$ (EGT vs. MT), $p = 5.74e-05$ (NUC vs. MT), $p = 5.932e-05$ (MT vs. Early EGT); GC1, $p = 4.73e-06$ (NUC vs. MT), $p = 0.002$ (NUC vs. EGT), $p = 0.002$ (EGT vs. Early EGT), $p = 5.01e-06$ (MT vs. Early EGT); GC3, $p = 0.044$ (EGT vs. MT), $p = 0.036$ (NUC vs. MT), $p = 0.036$ (MT vs. Early EGT). Source data are provided as a Source Data file. EGT, $n = 11$ CDSs; Early EGT, $n = 23$ CDSs; MT, $n = 18$ CDSs; NUC, $n = 8542$ CDSs. Box includes the 25$^{th}$ (Q1) to 75$^{th}$ (Q3) percentiles of the data with the median value in a thick line. Upper and lower whiskers indicate values within1.5 times interquartile range (Q3 – Q1) above 75$^{th}$ percentile and below 25$^{th}$ percentile, respectively.

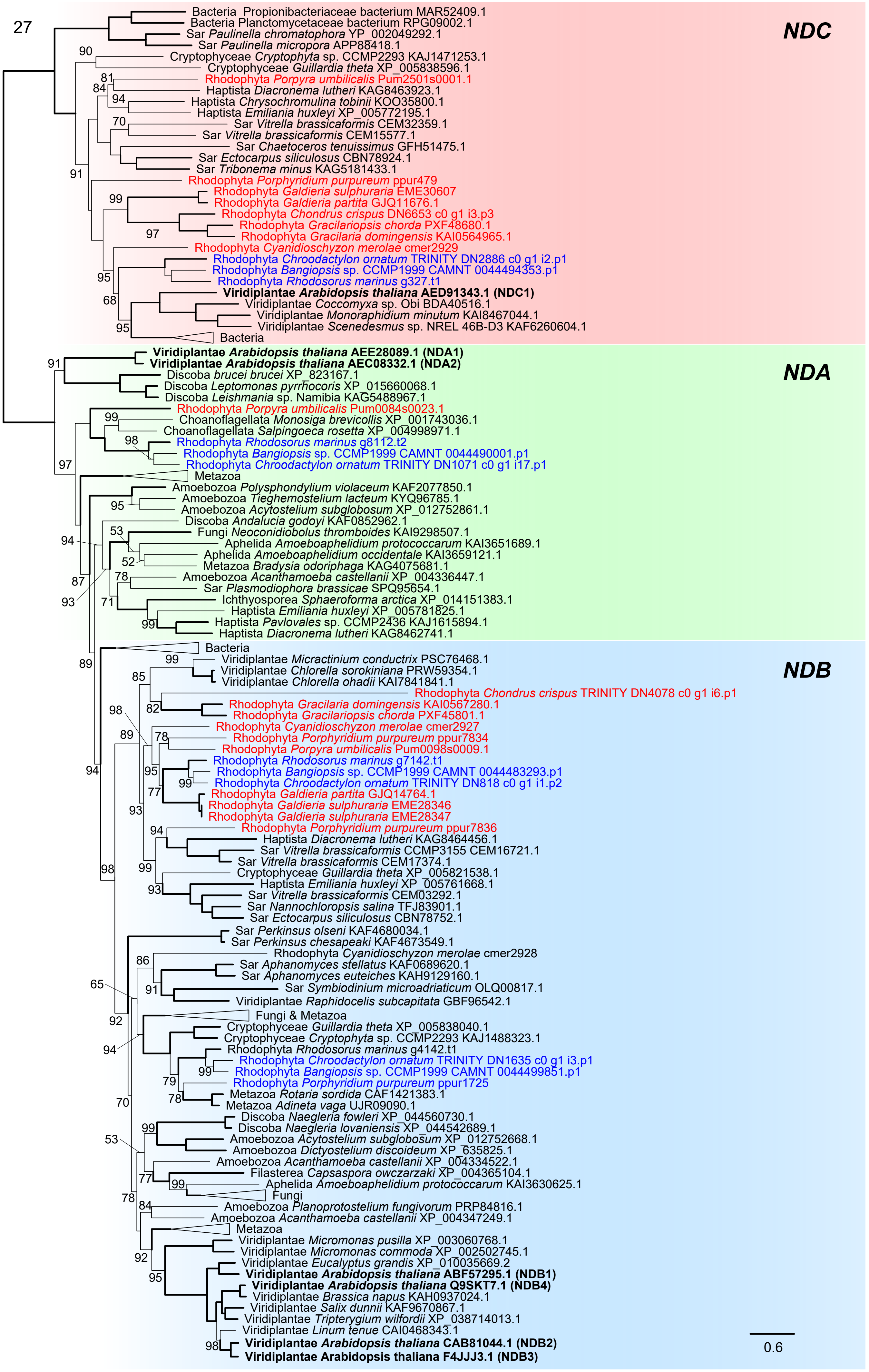
NDC
Bacteria Propionibacteriaceae bacterium MAR52409.1
Bacteria Planctomycetaceae bacterium RPG09002.1
Sar Paulinella chromatophora YP_002049292.1
Sar Paulinella micropora APP88418.1
Cryptophyceae Cryptophyta sp. CCMP2293 KAJ1471253.1
Cryptophyceae Guillardia theta XP_005838596.1
Rhodophyta Porpyra umbilicalis Pum2501s0001.1
Haptista Diacronema lutheri KAG8463923.1
Haptista Chrysochromulina tobinii KOO35800.1
Haptista Emiliania huxleyi XP_005772195.1
Sar Vitrella brassicaformis CEM32359.1
Sar Vitrella brassicaformis CEM15577.1
Sar Chaetoceros tenuissimus GFH51475.1
Sar Ectocarpus siliculosus CBN78924.1
Sar Tribonema minus KAG5181433.1
Rhodophyta Porphyridium purpureum ppur479
Rhodophyta Galdieria sulphuraria EME30607
Rhodophyta Galdieria partita GJQ11676.1
Rhodophyta Chondrus crispus DN6653 c0 g1 i3.p3
Rhodophyta Gracilariopsis chorda PXF48680.1
Rhodophyta Gracilaria domingensis KAI0564965.1
Rhodophyta Cyanidioschyzon merolae cmer2929
Rhodophyta Chroodactylon ornatum TRINITY DN2886 c0 g1 i2.p1
Rhodophyta Bangiopsis sp. CCMP1999 CAMNT 0044494353.p1
Rhodophyta Rhodosorus marinus g327.t1
Viridiplantae Arabidopsis thaliana AED91343.1 (NDC1)
Viridiplantae Coccomyxa sp. Obi BDA40516.1
Viridiplantae Monoraphidium minutum KAI8467044.1
Viridiplantae Scenedesmus sp. NREL 46B-D3 KAF6260604.1
Bacteria
NDA
Viridiplantae Arabidopsis thaliana AEE28089.1 (NDA1)
Viridiplantae Arabidopsis thaliana AEC08332.1 (NDA2)
Discoba brucei brucei XP_823167.1
Discoba Leptomonas pyrrhocoris XP_015660068.1
Discoba Leishmania sp. Namibia KAG5488967.1
Rhodophyta Porpyra umbilicalis Pum0084s0023.1
Choanoflagellata Monosiga brevicollis XP_001743036.1
Choanoflagellata Salpingoeca rosetta XP_004998971.1
Rhodophyta Rhodosorus marinus g8112.t2
Rhodophyta Bangiopsis sp. CCMP1999 CAMNT 0044490001.p1
Rhodophyta Chroodactylon ornatum TRINITY DN1071 c0 g1 i17.p1
Metazoa
Amoebozoa Polysphondylium violaceum KAF2077850.1
Amoebozoa Tieghemostelium lacteum KYQ96785.1
Amoebozoa Acytostelium subglobosum XP_012752861.1
Discoba Andalucia godoyi KAF0852962.1
Fungi Neoconidiobolus thromboides KAI9298507.1
Aphelida Amoeboaphelidium protococcarum KAI3651689.1
Aphelida Amoeboaphelidium occidentale KAI3659121.1
Metazoa Bradysia odoriphaga KAG4075681.1
Amoebozoa Acanthamoeba castellanii XP_004336447.1
Sar Plasmodiophora brassicae SPQ95654.1
Ichthyosporea Sphaeroforma arctica XP_014151383.1
Haptista Emiliania huxleyi XP_005781825.1
Haptista Pavlovales sp. CCMP2436 KAJ1615894.1
Haptista Diacronema lutheri KAG8462741.1
NDB
Bacteria
Viridiplantae Micractinium conductrix PSC76468.1
Viridiplantae Chlorella sorokiniana PRW59354.1
Viridiplantae Chlorella ohadii KAI7841841.1
Rhodophyta Chondrus crispus TRINITY DN4078 c0 g1 i6.p1
Rhodophyta Gracilaria domingensis KAI0567280.1
Rhodophyta Gracilariopsis chorda PXF45801.1
Rhodophyta Cyanidioschyzon merolae cmer2927
Rhodophyta Porphyridium purpureum ppur7834
Rhodophyta Porpyra umbilicalis Pum0098s0009.1
Rhodophyta Rhodosorus marinus g7142.t1
Rhodophyta Bangiopsis sp. CCMP1999 CAMNT 0044483293.p1
Rhodophyta Chroodactylon ornatum TRINITY DN818 c0 g1 i1.p2
Rhodophyta Galdieria partita GJQ14764.1
Rhodophyta Galdieria sulphuraria EME28346
Rhodophyta Galdieria sulphuraria EME28347
Rhodophyta Porphyridium purpureum ppur7836
Haptista Diacronema lutheri KAG8464456.1
Sar Vitrella brassicaformis CCMP3155 CEM16721.1
Sar Vitrella brassicaformis CEM17374.1
Cryptophyceae Guillardia theta XP_005821538.1
Haptista Emiliania huxleyi XP_005761668.1
Sar Vitrella brassicaformis CEM03292.1
Sar Nannochloropsis salina TFJ83901.1
Sar Ectocarpus siliculosus CBN78752.1
Sar Perkinsus olseni KAF4680034.1
Sar Perkinsus chesapeaki KAF4673549.1
Rhodophyta Cyanidioschyzon merolae cmer2928
Sar Aphanomyces stellatus KAF0689620.1
Sar Aphanomyces euteiches KAH9129160.1
Sar Symbiodinium microadriaticum OLQ00817.1
Viridiplantae Raphidocelis subcapitata GBF96542.1
Fungi & Metazoa
Cryptophyceae Guillardia theta XP_005838040.1
Cryptophyceae Cryptophyta sp. CCMP2293 KAJ1488323.1
Rhodophyta Rhodosorus marinus g4142.t1
Rhodophyta Chroodactylon ornatum TRINITY DN1635 c0 g1 i3.p1
Rhodophyta Bangiopsis sp. CCMP1999 CAMNT 0044499851.p1
Rhodophyta Porphyridium purpureum ppur1725
Metazoa Rotaria sordida CAF1421383.1
Metazoa Adineta vaga UJR09090.1
Discoba Naegleria fowleri XP_044560730.1
Discoba Naegleria lovaniensis XP_044542689.1
Amoebozoa Acytostelium subglobosum XP_012752668.1
Amoebozoa Dictyostelium discoideum XP_635825.1
Amoebozoa Acanthamoeba castellanii XP_004334522.1
Filasterea Capsaspora owczarzaki XP_004365104.1
Aphelida Amoeboaphelidium protococcarum KAI3630625.1
Fungi
Amoebozoa Planoprotostelium fungivorum PRP84816.1
Amoebozoa Acanthamoeba castellanii XP_004347249.1
Metazoa
Viridiplantae Micromonas pusilla XP_003060768.1
Viridiplantae Micromonas commoda XP_002502745.1
Viridiplantae Eucalyptus grandis XP_010035669.2
Viridiplantae Arabidopsis thaliana ABF57295.1 (NDB1)
Viridiplantae Arabidopsis thaliana Q9SKT7.1 (NDB4)
Viridiplantae Brassica napus KAH0937024.1
Viridiplantae Salix dunnii KAF9670867.1
Viridiplantae Tripterygium wilfordii XP_038714013.1
Viridiplantae Linum tenue CAI0468343.1
Viridiplantae Arabidopsis thaliana CAB81044.1 (NDB2)
Viridiplantae Arabidopsis thaliana F4JJJ3.1 (NDB3)
0.6

**Supplementary Fig. 13. Phylogenetic tree of alternative NAD(P)H dehydrogenases.** Three subfamilies (*NDA*, *NDB*, and *NDC*) were identified based on genes from *Arabidopsis thaliana*. *NDC* clade was used as root. Although none of the additional genes are specific to the Stylonematophyceae, only the Stylonematophyceae has all the red algal alternative NAD(P)H dehydrogenases. The Stylonematophyceae is colored in blue and the other red algae are colored in red. Bootstrap values <50% are not shown. The thick branches indicate 100% bootstrap values.

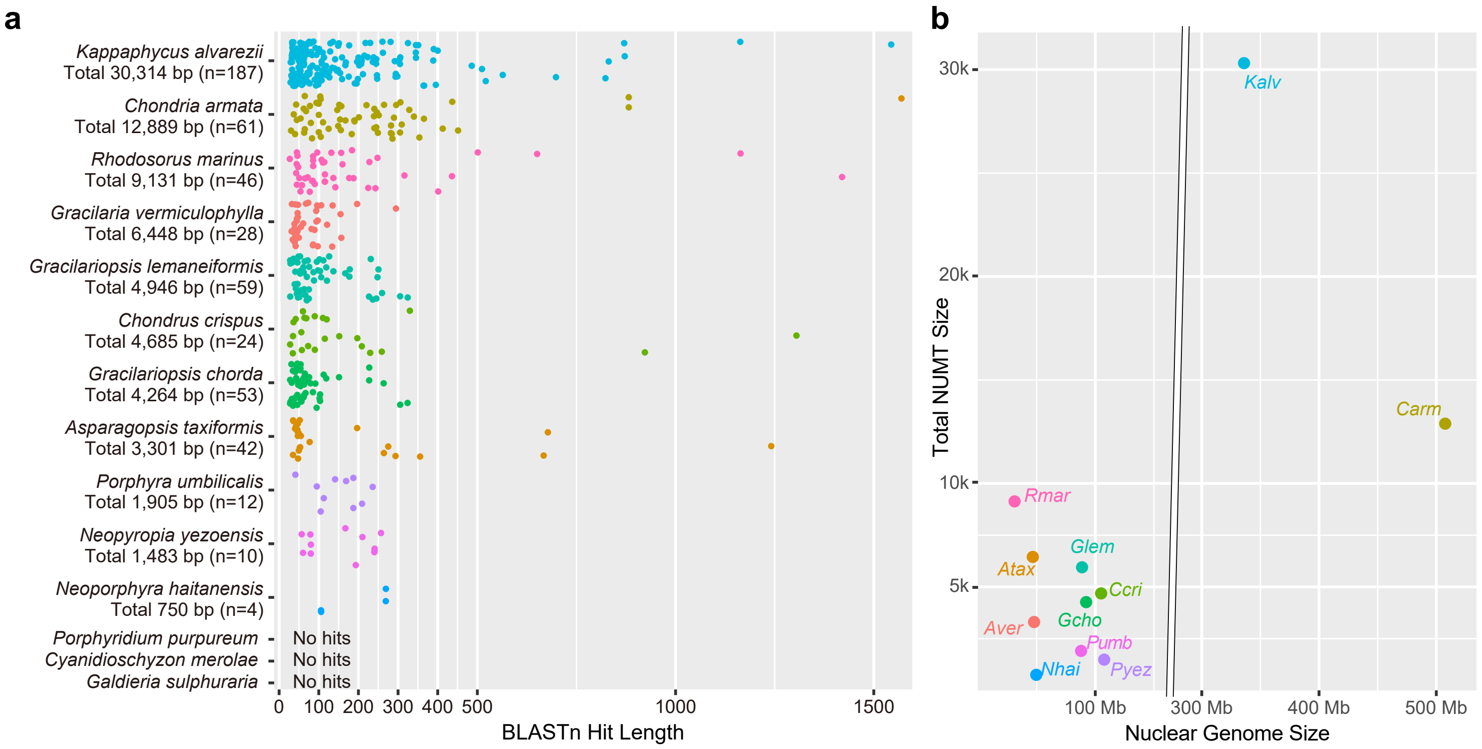


**Supplementary Fig. 14. Length distribution of NUMTs in 14 species. (a)** Scattered dot plot indicating the length of BLASTn hits, total hit, and length. *R. marinus* has the third longest NUMT. *C. merolae*, *G. sulphuraria*, and *P. purpureum* did not have significant hits. **(b)** Dot plot showing proportion of total NUMT size to nuclear genome size. Two species with the longest NUMTs have incomparably larger nuclear genome sizes. However, *R. marinus* has the third longest NUMT while having the smallest nuclear genome size. Thus, the proportion of NUMT to nuclear genome size is the highest in *R. marinus*. Source data are provided as a Source Data file.

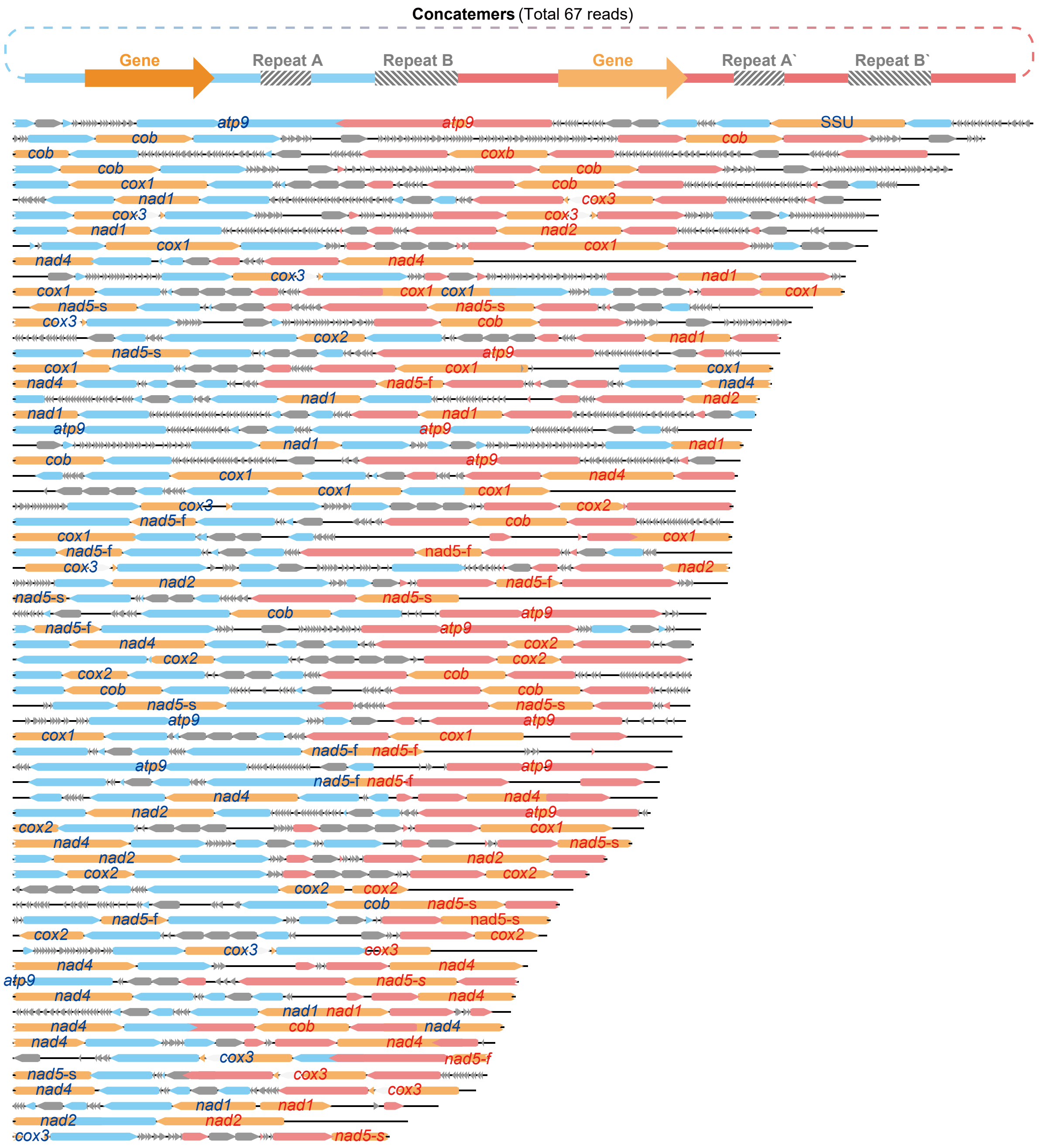


**Supplementary Fig. 15. Structure and composition of full sets of concatemers.** Concatemers with variable lengths consistently have an identical structure, shown at the top. Blue and red boxes represent the NCR of the former and the latter genes.

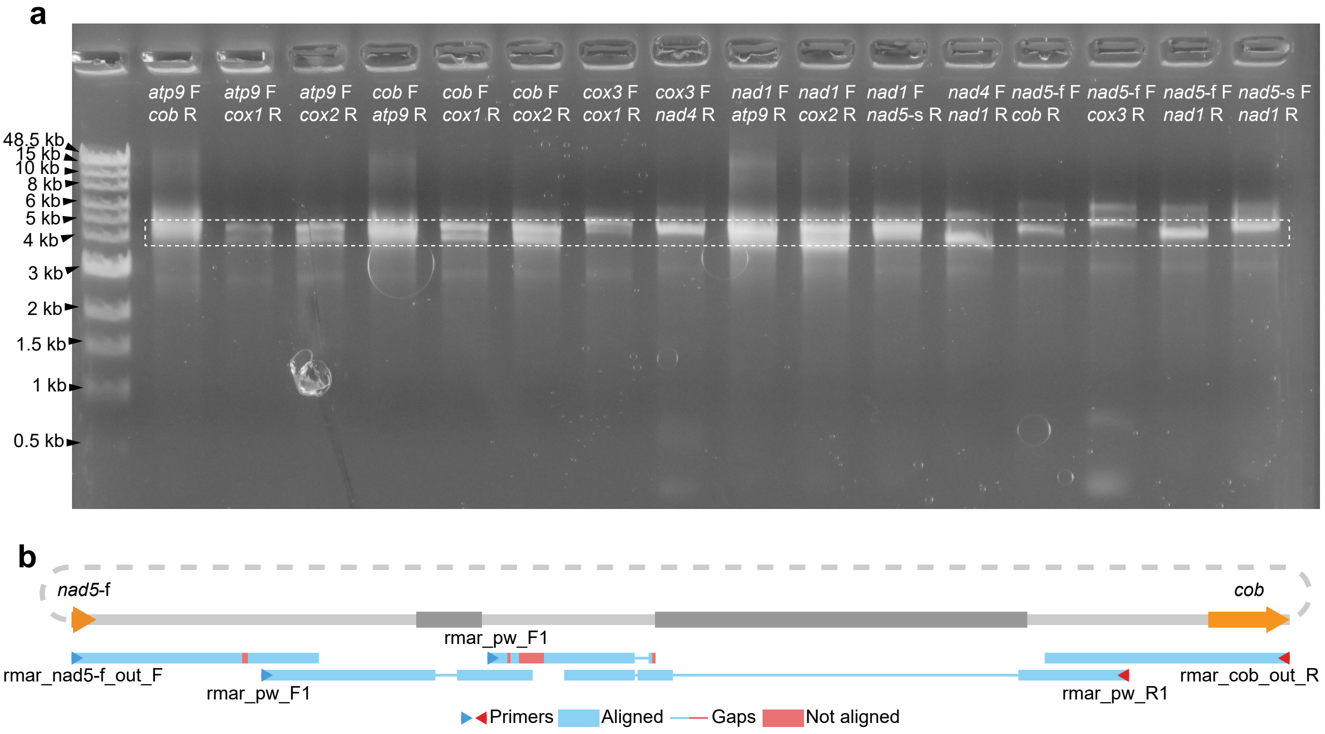


**Supplementary Fig. 16. PCR verification of hetero-concatemers. (a)** Primers designed for different minicircles were used to identify presence of hetero-concatemers and recombination. White dashed box indicates target PCR products. Bands with expected lengths are consistently shown in all the combinations. Only PCR products 16 combinations are displayed. Representative images of $n$ = 4 biological replicates. **(b)** PCR products mapped to *nad5*-f+*cob* concatemer. Except repetitive regions that were not sequenced, all the other regions were covered.

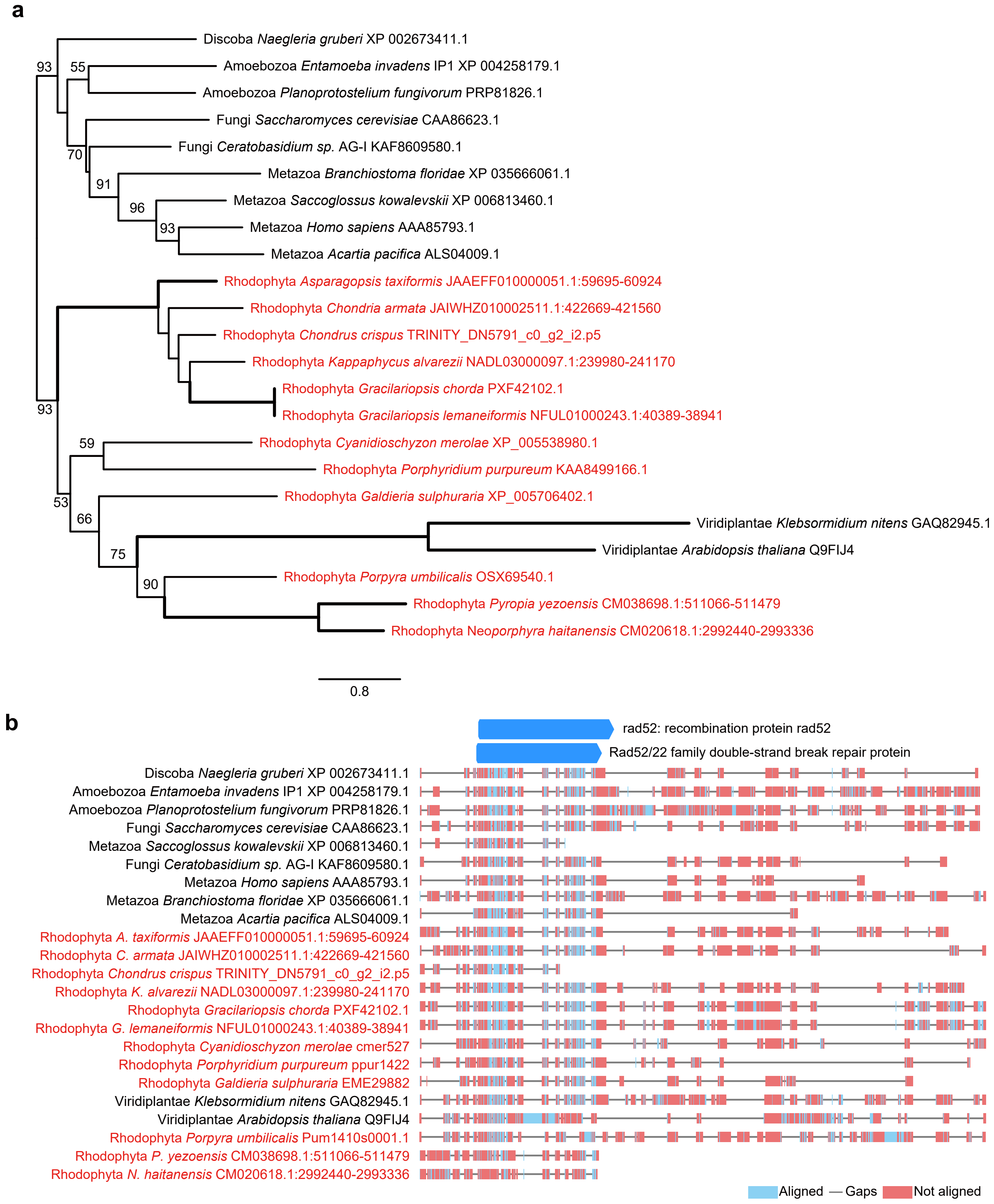
a
Discoba Naegleria gruberi XP 002673411.1
Amoebozoa Entamoeba invadens IP1 XP 004258179.1
Amoebozoa Planoprotostelium fungivorum PRP81826.1
Fungi Saccharomyces cerevisiae CAA86623.1
Fungi Ceratobasidium sp. AG-I KAF8609580.1
Metazoa Branchiostoma floridae XP 035666061.1
Metazoa Saccoglossus kowalevskii XP 006813460.1
Metazoa Homo sapiens AAA85793.1
Metazoa Acartia pacifica ALS04009.1
Rhodophyta Asparagopsis taxiformis JAAEFF010000051.1:59695-60924
Rhodophyta Chondria armata JAIWHZ010002511.1:422669-421560
Rhodophyta Chondrus crispus TRINITY_DN5791_c0_g2_i2.p5
Rhodophyta Kappaphycus alvarezii NADL03000097.1:239980-241170
Rhodophyta Gracilariopsis chorda PXF42102.1
Rhodophyta Gracilariopsis lemaneiformis NFUL01000243.1:40389-38941
Rhodophyta Cyanidioschyzon merolae XP_005538980.1
Rhodophyta Porphyridium purpureum KAA8499166.1
Rhodophyta Galdieria sulphuraria XP_005706402.1
Viridiplantae Klebsormidium nitens GAQ82945.1
Viridiplantae Arabidopsis thaliana Q9FIJ4
Rhodophyta Porpyra umbilicalis OSX69540.1
Rhodophyta Pyropia yezoensis CM038698.1:511066-511479
Rhodophyta Neoporphyra haitanensis CM020618.1:2992440-2993336
93
55
70
91
96
93
93
59
53
66
75
90
0.8
b
rad52: recombination protein rad52
Rad52/22 family double-strand break repair protein
Discoba Naegleria gruberi XP 002673411.1
Amoebozoa Entamoeba invadens IP1 XP 004258179.1
Amoebozoa Planoprotostelium fungivorum PRP81826.1
Fungi Saccharomyces cerevisiae CAA86623.1
Metazoa Saccoglossus kowalevskii XP 006813460.1
Fungi Ceratobasidium sp. AG-I KAF8609580.1
Metazoa Homo sapiens AAA85793.1
Metazoa Branchiostoma floridae XP 035666061.1
Metazoa Acartia pacifica ALS04009.1
Rhodophyta A. taxiformis JAAEFF010000051.1:59695-60924
Rhodophyta C. armata JAIWHZ010002511.1:422669-421560
Rhodophyta Chondrus crispus TRINITY_DN5791_c0_g2_i2.p5
Rhodophyta K. alvarezii NADL03000097.1:239980-241170
Rhodophyta Gracilariopsis chorda PXF42102.1
Rhodophyta G. lemaneiformis NFUL01000243.1:40389-38941
Rhodophyta Cyanidioschyzon merolae cmer527
Rhodophyta Porphyridium purpureum ppur1422
Rhodophyta Galdieria sulphuraria EME29882
Viridiplantae Klebsormidium nitens GAQ82945.1
Viridiplantae Arabidopsis thaliana Q9FIJ4
Rhodophyta Porpyra umbilicalis Pum1410s0001.1
Rhodophyta P. yezoensis CM038698.1:511066-511479
Rhodophyta N. haitanensis CM020618.1:2992440-2993336
Aligned
Gaps
Not aligned

**Supplementary Fig. 17. Phylogenetic tree and alignment of *RAD52*. (a)** Phylogenetic tree of *RAD52*. Red algal *RAD52* forms a well-supported monophyletic group with Viridiplantae. Among red algae, only the Stylonematophyceae lacks *RAD52*. Red algae are colored red. Bootstrap values <50% are not shown. The thick branch indicates bootstrap values of 100%. **(b)** Alignments of RAD52. Predicted domains from *Saccharomyces cerevisiae* and *Arabidopsis thaliana* are shown above the alignment. RAD52 domains are generally conserved across lineages. The blue and red boxes indicate aligned and unaligned regions, respectively. The grey line indicates gaps. Amino acids with over 50% frequency at each site are considered to be aligned. Gaps are ignored.

a

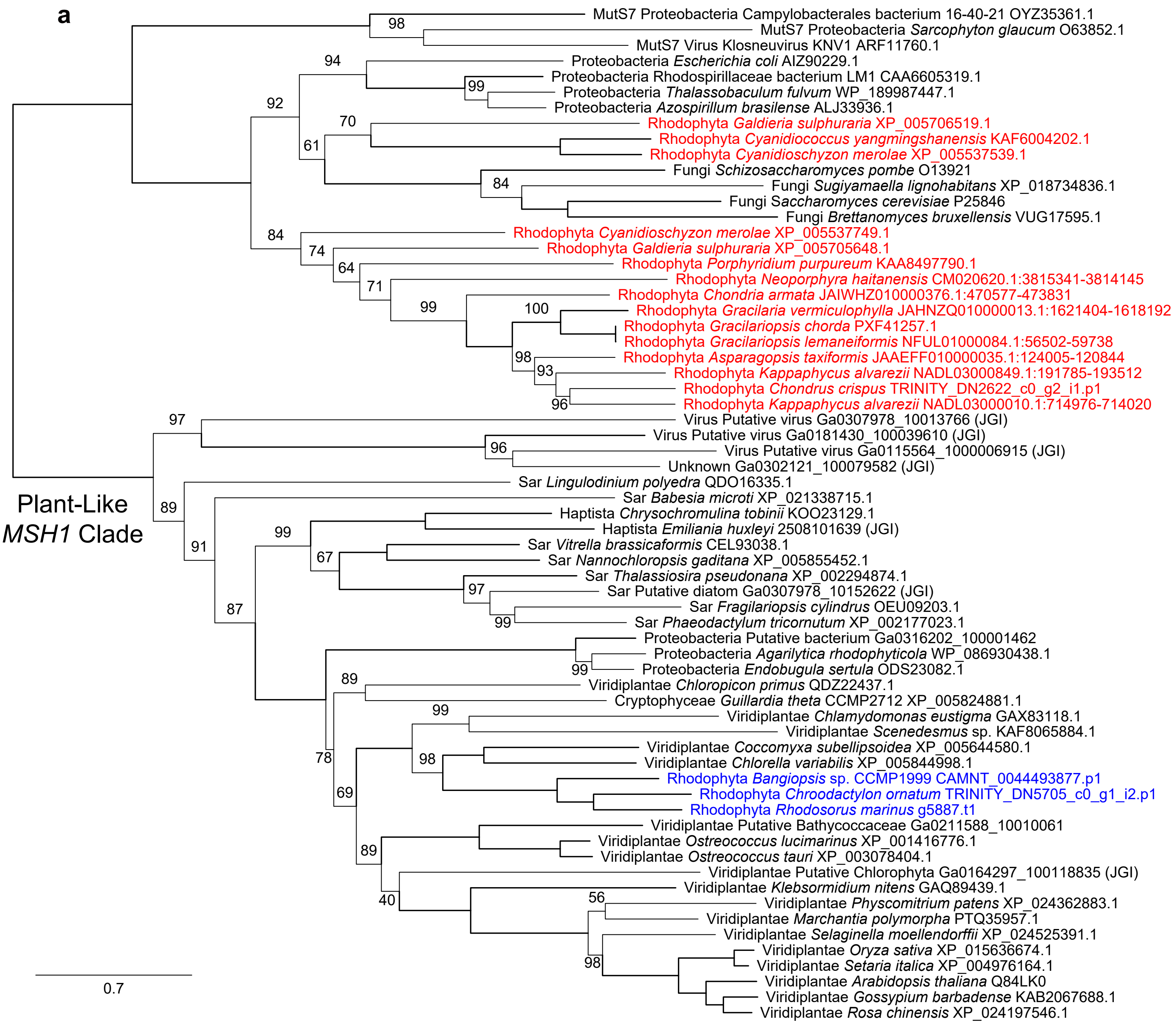
MutS7 Proteobacteria Campylobacterales bacterium 16-40-21 OYZ35361.1
MutS7 Proteobacteria Sarcophyton glaucum O63852.1
MutS7 Virus Klosneuvirus KNV1 ARF11760.1
Proteobacteria Escherichia coli AIZ90229.1
Proteobacteria Rhodospirillaceae bacterium LM1 CAA6605319.1
Proteobacteria Thalassobaculum fulvum WP_189987447.1
Proteobacteria Azospirillum brasilense ALJ33936.1
Rhodophyta Galdieria sulphuraria XP_005706519.1
Rhodophyta Cyanidiococcus yangmingshanensis KAF6004202.1
Rhodophyta Cyanidioschyzon merolae XP_005537539.1
Fungi Schizosaccharomyces pombe O13921
Fungi Sugiyamaella lignohabitans XP_018734836.1
Fungi Saccharomyces cerevisiae P25846
Fungi Brettanomyces bruxellensis VUG17595.1
Rhodophyta Cyanidioschyzon merolae XP_005537749.1
Rhodophyta Galdieria sulphuraria XP_005705648.1
Rhodophyta Porphyridium purpureum KAA8497790.1
Rhodophyta Neoporphyra haitanensis CM020620.1:3815341-3814145
Rhodophyta Chondria armata JAIWHZ010000376.1:470577-473831
Rhodophyta Gracilaria vermiculophylla JAHNZQ010000013.1:1621404-1618192
Rhodophyta Gracilariopsis chorda PXF41257.1
Rhodophyta Gracilariopsis lemaneiformis NFUL01000084.1:56502-59738
Rhodophyta Asparagopsis taxiformis JAAEFF010000035.1:124005-120844
Rhodophyta Kappaphycus alvarezii NADL03000849.1:191785-193512
Rhodophyta Chondrus crispus TRINITY_DN2622_c0_g2_i1.p1
Rhodophyta Kappaphycus alvarezii NADL03000010.1:714976-714020
Virus Putative virus Ga0307978_10013766 (JGI)
Virus Putative virus Ga0181430_100039610 (JGI)
Virus Putative virus Ga0115564_1000006915 (JGI)
Unknown Ga0302121_100079582 (JGI)
Sar Lingulodinium polyedra QDO16335.1
Sar Babesia microti XP_021338715.1
Haptista Chrysochromulina tobinii KOO23129.1
Haptista Emiliania huxleyi 2508101639 (JGI)
Sar Vitrella brassicaformis CEL93038.1
Sar Nannochloropsis gaditana XP_005855452.1
Sar Thalassiosira pseudonana XP_002294874.1
Sar Putative diatom Ga0307978_10152622 (JGI)
Sar Fragilariopsis cylindrus OEU09203.1
Sar Phaeodactylum tricornutum XP_002177023.1
Proteobacteria Putative bacterium Ga0316202_100001462
Proteobacteria Agarilytica rhodophyticola WP_086930438.1
Proteobacteria Endobugula sertula ODS23082.1
Viridiplantae Chloropicon primus QDZ22437.1
Cryptophyceae Guillardia theta CCMP2712 XP_005824881.1
Viridiplantae Chlamydomonas eustigma GAX83118.1
Viridiplantae Scenedesmus sp. KAF8065884.1
Viridiplantae Coccomyxa subellipsoidea XP_005644580.1
Viridiplantae Chlorella variabilis XP_005844998.1
Rhodophyta Bangiopsis sp. CCMP1999 CAMNT_0044493877.p1
Rhodophyta Chroodactylon ornatum TRINITY_DN5705_c0_g1_i2.p1
Rhodophyta Rhodosorus marinus g5887.t1
Viridiplantae Putative Bathycoccaceae Ga0211588_10010061
Viridiplantae Ostreococcus lucimarinus XP_001416776.1
Viridiplantae Ostreococcus tauri XP_003078404.1
Viridiplantae Putative Chlorophyta Ga0164297_100118835 (JGI)
Viridiplantae Klebsormidium nitens GAQ89439.1
Viridiplantae Physcomitrium patens XP_024362883.1
Viridiplantae Marchantia polymorpha PTQ35957.1
Viridiplantae Selaginella moellendorffii XP_024525391.1
Viridiplantae Oryza sativa XP_015636674.1
Viridiplantae Setaria italica XP_004976164.1
Viridiplantae Arabidopsis thaliana Q84LK0
Viridiplantae Gossypium barbadense KAB2067688.1
Viridiplantae Rosa chinensis XP_024197546.1
Plant-Like MSH1 Clade
0.7

**b**

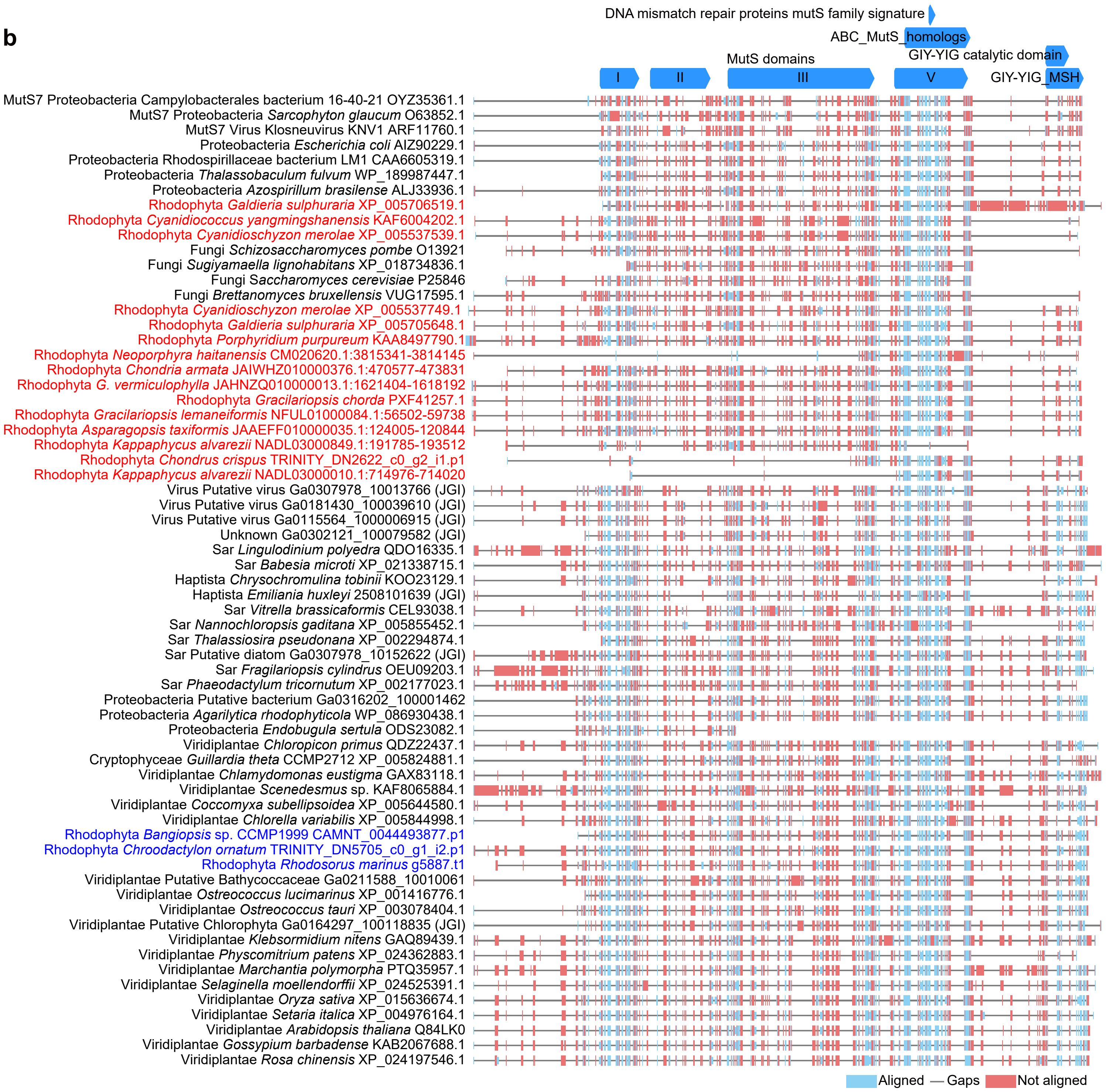
DNA mismatch repair proteins mutS family signature
ABC_MutS_homologs
GIY-YIG catalytic domain
MutS domains
I
II
III
V
GIY-YIG_MSH
MutS7 Proteobacteria Campylobacterales bacterium 16-40-21 OYZ35361.1
MutS7 Proteobacteria *Sarcophyton glaucum* O63852.1
MutS7 Virus Klosneuvirus KNV1 ARF11760.1
Proteobacteria *Escherichia coli* AIZ90229.1
Proteobacteria Rhodospirillaceae bacterium LM1 CAA6605319.1
Proteobacteria *Thalassobaculum fulvum* WP_189987447.1
Proteobacteria *Azospirillum brasilense* ALJ33936.1
Rhodophyta *Galdieria sulphuraria* XP_005706519.1
Rhodophyta *Cyanidiococcus yangmingshanensis* KAF6004202.1
Rhodophyta *Cyanidioschyzon merolae* XP_005537539.1
Fungi *Schizosaccharomyces pombe* O13921
Fungi *Sugiyamaella lignohabitans* XP_018734836.1
Fungi *Saccharomyces cerevisiae* P25846
Fungi *Brettanomyces bruxellensis* VUG17595.1
Rhodophyta *Cyanidioschyzon merolae* XP_005537749.1
Rhodophyta *Galdieria sulphuraria* XP_005705648.1
Rhodophyta *Porphyridium purpureum* KAA8497790.1
Rhodophyta *Neoporphyra haitanensis* CM020620.1:3815341-3814145
Rhodophyta *Chondria armata* JAIWHZ010000376.1:470577-473831
Rhodophyta *G. vermiculophylla* JAHNZQ010000013.1:1621404-1618192
Rhodophyta *Gracilariopsis chorda* PXF41257.1
Rhodophyta *Gracilariopsis lemaneiformis* NFUL01000084.1:56502-59738
Rhodophyta *Asparagopsis taxiformis* JAAEFF010000035.1:124005-120844
Rhodophyta *Kappaphycus alvarezii* NADL03000849.1:191785-193512
Rhodophyta *Chondrus crispus* TRINITY_DN2622_c0_g2_i1.p1
Rhodophyta *Kappaphycus alvarezii* NADL03000010.1:714976-714020
Virus Putative virus Ga0307978_10013766 (JGI)
Virus Putative virus Ga0181430_100039610 (JGI)
Virus Putative virus Ga0115564_1000006915 (JGI)
Unknown Ga0302121_100079582 (JGI)
Sar *Lingulodinium polyedra* QDO16335.1
Sar *Babesia microti* XP_021338715.1
Haptista *Chrysochromulina tobinii* KOO23129.1
Haptista *Emiliania huxleyi* 2508101639 (JGI)
Sar *Vitrella brassicaformis* CEL93038.1
Sar *Nannochloropsis gaditana* XP_005855452.1
Sar *Thalassiosira pseudonana* XP_002294874.1
Sar Putative diatom Ga0307978_10152622 (JGI)
Sar *Fragilariopsis cylindrus* OEU09203.1
Sar *Phaeodactylum tricornutum* XP_002177023.1
Proteobacteria Putative bacterium Ga0316202_100001462
Proteobacteria *Agarilytica rhodophyticola* WP_086930438.1
Proteobacteria *Endobugula sertula* ODS23082.1
Viridiplantae *Chloropicon primus* QDZ22437.1
Cryptophyceae *Guillardia theta* CCMP2712 XP_005824881.1
Viridiplantae *Chlamydomonas eustigma* GAX83118.1
Viridiplantae *Scenedesmus* sp. KAF8065884.1
Viridiplantae *Coccomyxa subellipsoidea* XP_005644580.1
Viridiplantae *Chlorella variabilis* XP_005844998.1
Rhodophyta *Bangiopsis* sp. CCMP1999 CAMNT_0044493877.p1
Rhodophyta *Chroodactylon ornatum* TRINITY_DN5705_c0_g1_i2.p1
Rhodophyta *Rhodosorus marinus* g5887.t1
Viridiplantae Putative Bathycoccaceae Ga0211588_10010061
Viridiplantae *Ostreococcus lucimarinus* XP_001416776.1
Viridiplantae *Ostreococcus tauri* XP_003078404.1
Viridiplantae Putative Chlorophyta Ga0164297_100118835 (JGI)
Viridiplantae *Klebsormidium nitens* GAQ89439.1
Viridiplantae *Physcomitrium patens* XP_024362883.1
Viridiplantae *Marchantia polymorpha* PTQ35957.1
Viridiplantae *Selaginella moellendorffii* XP_024525391.1
Viridiplantae *Oryza sativa* XP_015636674.1
Viridiplantae *Setaria italica* XP_004976164.1
Viridiplantae *Arabidopsis thaliana* Q84LK0
Viridiplantae *Gossypium barbadense* KAB2067688.1
Viridiplantae *Rosa chinensis* XP_024197546.1
Aligned
Gaps
Not aligned

**Supplementary Fig. 18. Phylogenetic tree and alignment of *MSH1*. (a)** Phylogenetic tree of *MSH1*. *MutS7*+*MutS1*+Fungal *MSH1* clade was used as root [19]. Stylonematophyceae *MSH1* forms a monophyletic group with Viridiplantae and Cryptophyceae, which was formerly described as Plant-Like *MSH1* Clade [19], but not with the other red algae. The Stylonematophyceae is colored in blue and the other red algae are colored in red. Bootstrap values <50% are not shown. The thick branches indicate 100% bootstrap values. **(b)** Alignments of MSH1. Predicted domains from *Arabidopsis thaliana* are shown above the alignment. Note that Stylonematophyceae MSH1 has a C-terminal domain, whereas the other red algal MSH1 proteins do not. The blue and red boxes indicate aligned and unaligned regions, respectively. The grey line indicates gaps. Amino acids with >50% frequency at each site is considered to be aligned. Gaps are ignored.

## Supplementary References


1. Lee S*, et al.* Investigation of various fluorescent protein–DNA binding peptides for effectively visualizing large DNA molecules. *RSC Advances* **6**, 46291-46298 (2016).
2. Kim T*, et al.* Counting DNA molecules on a microchannel surface for quantitative analysis. *Talanta* **252**, 123826 (2023).
3. Kosar M, Piccini D, Foiani M, Giannattasio M. A rapid method to visualize human mitochondrial DNA replication through rotary shadowing and transmission electron microscopy. *Nucleic Acids Research* **49**, e121-e121 (2021).
4. Li WH, Wu CI, Luo CC. A new method for estimating synonymous and nonsynonymous rates of nucleotide substitution considering the relative likelihood of nucleotide and codon changes. *Molecular Biology and Evolution* **2**, 150-174 (1985).
5. Yankovskaya V*, et al.* Architecture of succinate dehydrogenase and reactive oxygen species generation. *Science* **299**, 700-704 (2003).
6. Maklashina E, Rothery RA, Weiner JH, Cecchini G. Retention of heme in axial ligand mutants of succinate-ubiquinone oxidoreductase (Complex II) from *Escherichia coli*. *Journal of Biological Chemistry* **276**, 18968-18976 (2001).
7. Pasini B, Stratakis CA. SDH mutations in tumorigenesis and inherited endocrine tumours: lesson from the phaeochromocytoma–paraganglioma syndromes. *Journal of Internal Medicine* **266**, 19-42 (2009).
8. Lambowitz AM, Zimmerly S. Mobile Group II Introns. *Annual Review of Genetics* **38**, 1-35 (2004).
9. Zimmerly S, Semper C. Evolution of group II introns. *Mobile DNA* **6**, 7 (2015).
10. Knoop V, Brennicke A. Promiscuous mitochondrial group II intron sequences in plant nuclear genomes. *Journal of Molecular Evolution* **39**, 144-150 (1994).
11. Lin X*, et al.* Sequence and analysis of chromosome 2 of the plant *Arabidopsis thaliana*. *Nature* **402**, 761-768 (1999).
12. Doolittle WF. The trouble with (group II) introns. *Proceedings of the National Academy of Sciences* **111**, 6536-6537 (2014).
13. Mukhopadhyay J, Hausner G. Organellar introns in fungi, algae, and plants (2021).
14. Novikova O, Belfort M. Mobile group II introns as ancestral eukaryotic elements. *Trends in Genetics* **33**, 773-783 (2017).
15. Chalamcharla VR, Curcio MJ, Belfort M. Nuclear expression of a group II intron is consistent with spliceosomal intron ancestry. *Genes & Development* **24**, 827-836 (2010).

16. Qu G*, et al.* RNA–RNA interactions and pre-mRNA mislocalization as drivers of group II intron loss from nuclear genomes. *Proceedings of the National Academy of Sciences* **111**, 6612-6617 (2014).
17. Kleine T, Maier UG, Leister D. DNA transfer from organelles to the nucleus: the idiosyncratic genetics of endosymbiosis. *Annual Review of Plant Biology* **60**, 115-138 (2009).
18. Yu R*, et al.* The minicircular and extremely heteroplasmic mitogenome of the holoparasitic plant *Rhopalocnemis phalloides*. *Current Biology* **32**, 470-479.e475 (2022).
19. Wu Z, Waneka G, Broz AK, King CR, Sloan DB. MSH1 is required for maintenance of the low mutation rates in plant mitochondrial and plastid genomes. *Proceedings of the National Academy of Sciences* **117**, 16448-16455 (2020).
