# Supplementary material for: Origin of minicircular mitochondrial genomes in red algae
Source: Nat Commun. 2023 Jun 8;14:3363. doi: 10.1038/s41467-023-39084-2 (PMC10250338; doi:10.1038/s41467-023-39084-2)
Supplement: Supplementary file 2 — Description of Additional Supplementary Files [file 41467_2023_39084_MOESM2_ESM.pdf]

Description of Additional Supplementary Files

Supplementary Data 1. Species information. Data generated in this study are shown in bold.

Supplementary Data 2. Primers used in this study.

Supplementary Data 3. qPCR information. Relativeness is a proportion of average copy number of mitogenomes to that of nuclear genome. The sdhB, and actin genes were used to estimate the copy number of nuclear genes.

Supplementary Data 4. EGT-derived mitochondrial genes.

Supplementary Data 5. Ribosomal gene clusters in 30 red algal mitochondria

Supplementary Data 6. targetP result about EGT-derived genes.

Supplementary Data 7. Genomes used in NUMT analysis. Data generated in this study are shown in bold. Values refer to information from NCBI genome database.

Supplementary Data 8. DNA-RRR genes that control organelle genome stability.
