# Supplementary material for: Origin of minicircular mitochondrial genomes in red algae
Source: Nat Commun. 2023 Jun 8;14:3363. doi: 10.1038/s41467-023-39084-2 (PMC10250338; doi:10.1038/s41467-023-39084-2)
Supplement: Supplementary file 4 — Reporting Summary [file 41467_2023_39084_MOESM4_ESM.pdf]

nature portfolio

Corresponding author(s): Kyubong Jo, Hwan Su Yoon

Last updated by author(s): May 10, 2023

Reporting Summary

Nature Portfolio wishes to improve the reproducibility of the work that we publish. This form provides structure for consistency and transparency in reporting. For further information on Nature Portfolio policies, see our Editorial Policies and the Editorial Policy Checklist.

Statistics

For all statistical analyses, confirm that the following items are present in the figure legend, table legend, main text, or Methods section.

n/a	Confirmed	
☐	☒	The exact sample size (*n*) for each experimental group/condition, given as a discrete number and unit of measurement
☐	☒	A statement on whether measurements were taken from distinct samples or whether the same sample was measured repeatedly
☐	☒	The statistical test(s) used AND whether they are one- or two-sided<br>*Only common tests should be described solely by name; describe more complex techniques in the Methods section.*
☒	☐	A description of all covariates tested
☐	☒	A description of any assumptions or corrections, such as tests of normality and adjustment for multiple comparisons
☐	☒	A full description of the statistical parameters including central tendency (e.g. means) or other basic estimates (e.g. regression coefficient) AND variation (e.g. standard deviation) or associated estimates of uncertainty (e.g. confidence intervals)
☒	☐	For null hypothesis testing, the test statistic (e.g. *F*, *t*, *r*) with confidence intervals, effect sizes, degrees of freedom and *P* value noted<br>*Give P values as exact values whenever suitable.*
☒	☐	For Bayesian analysis, information on the choice of priors and Markov chain Monte Carlo settings
☒	☐	For hierarchical and complex designs, identification of the appropriate level for tests and full reporting of outcomes
☒	☐	Estimates of effect sizes (e.g. Cohen's *d*, Pearson's *r*), indicating how they were calculated

Our web collection on statistics for biologists contains articles on many of the points above.

Software and code

Policy information about availability of computer code

Data collection	Whole genome sequencing and genome assembly<br>Library preparation and whole genome sequencing for both short-read and long-read sequencing were carried out by DNA Link Inc. (Seoul, Korea). For short-read sequencing, libraries were prepared using the Truseq Nano DNA Prep Kit (550 bp Protocol) and sequencing was done with the Illumina HiSeq2500 platform according to the protocol using 100 bp paired-end reagents. Long-read sequencing was carried out with Oxford Nanopore platform (ONT GridION) for R. marinus and the Pacific Biosciences (PacBio) High-Fidelity (HiFi) sequencing platform for C. ornatum. RNA-seq for R. marinus was done with the Illumina NovaSeq600 platform. The raw data from short-read sequencing were assembled using SPAdes 3.14.1 with '—careful' pipeline option and those from long-read sequencing were assembled using NextDenovo 2.5.0 (https://github.com/Nextomics/NextDenovo) for nuclear genome of R. marinus. Assembled NextDenovo contigs were polished 3 times with Pilon 1.22 using short-read mapping data generated by bowtie2 2.3.5.1. For mitogenome assemblies using long-read data, reads that have BLAST hits to mitochondrial CDS were used. The program miniasm 0.3 (r179) was used to identify the R. marinus mitogenome and IPA 1.3.1 was used for C. ornatum. In addition, reads that had BLAST hits to the NCR were used to search for "empty" minicircle reads that do not contain a CDS, however, no contigs were assembled, meaning the collected reads are just fragments of CDS-containing reads. Because minicircles share long conserved region that short-reads cannot discriminate, we used long-read data and NextPolish 1.4.0 to polish the miniasm-derived contigs. We did not perform polishing on IPA contigs, because HiFi sequencing generates extremely accurate reads. Remaining SNPs and ambiguities were manually corrected using mapping data of long-reads containing CDS. For C. ornatum, each sequence from step 10 (10-assemble/p_ctg.fasta) was considered as a minicircle sequence, because the following step of the IPA assembler (polish and purge dups) did not function correctly.<br>For the short-read data, sorted and verified mitochondrial genes (see below) were used as seeds for NOVOplasty 4.2. Using Geneious (Biomatters, Auckland, New Zealand), generated NOVOplasty contigs were then de novo assembled. Assembled contig that codes any of mitochondrial genes was considered as part of mitochondrial genome. Those contigs were polished (-SNP & Indel) with Pilon 1.22, using short-read mapping data generated by bowtie2 2.3.5.1. Trinity 2.11.0 was used to assemble RNA sequencing data.

Microchannel and Positive surface preparation
Polydimethylsiloxane (PDMS) devices and positively charged surfaces were prepared as previously described. In particular, microchannel template was utilized to create two layers on a silicon wafer through repeated photolithography procedures, following the protocol specified in the Kayaku Advanced Materials SU-8 2000 datasheet. First, a silicon wafer was spin-coated with a 20 µm layer of SU-8 2015 photoresist (Kayaku Advanced Materials, Massachusetts, USA) using a spin coater (Midas System SPIN-1200D, Daejeon, Korea). Subsequently, the spin-coated wafer was exposed to 350 nm radiation with an aligner (Midas System MDA-400LJ, Daejeon, Korea) through a mask and developed using an SU-8 developer (Kayaku Advanced Materials, Massachusetts, USA). Next, SU-8 TF 6002 was spin-coated as a second layer on top of the first layer. Since SmartPrint (SmartForce Technologies, La Tronche, France) is compatible with g-line photoresists, SU-8 TF 6002 was used. Following fabrication of the template wafer, the outlet port was created by attaching a tube to the peak area of the triangular section. At last, microchannel template was placed onto a silicon wafer. Then, a mixture (10:1 wt ratio) of PDMS pre-polymer and curing agent (K1 solution, Gwangmyeong, Korea) was poured onto it and incubated at 65 °C for 12 hours. The resulting PDMS layer was peeled from the wafer, and a chamber was created by physically punching a channel into it. The PDMS microchannel underwent oxidation for 30 seconds at 100 W in an air plasma generator (Femto Science Cute Basic, Korea). Finally, the PDMS device was washed and stored in deionized water.
Silicon wafers (Wafer market, Yongin, Korea) were purchased with a 30 nm SiO2 layer on top. To eliminate the polymer coating, oxidized silicon wafers and glass coverslips were arranged in a Teflon rack and soaked in piranha etching solution (30:70 v/v H2O2/H2SO4) for 3 hours. The wafers and coverslips were washed thoroughly with deionized water. Neutral pH of 7 was achieved and confirmed by pH paper. Subsequently, the wafers and coverslips were sonicated in deionized water for 30 minutes, followed by another round of rinsing with deionized water, to uncover the piranha surface. In the end, solutions with a concentration of 1.1 mM were prepared by adding 150 µl of Q-siloxane in 50% methanol to 250 mL of deionized water. Wafers and coverslips were incubated at 65 °C and 100 rpm for 16 h. Finally, they were rinsed three times with 99.9% ethanol and stored in 99.9% ethanol.

DNA molecule visualization under FM and SEM
DNA molecules pre-mixed with FP-DBP were stained with 5 % polyvinylpyrrolidone (PVP, molecular weight (MW): 40 000) solution. Stained DNA molecules were elongated and immobilized on a positively charged surface using a PDMS microfluidic device. DNA molecules were imaged under a FM. The microscopy system consisted of an inverted microscope (Olympus IX70, Japan) equipped with 100× Olympus UPlanSApo oil immersion objectives and an illuminated LED light source (SOLA SM 2 light engine, Lumencor, OR). Fluorescence images were captured using a scientific complementary metal-oxide semiconductor (sCMOS) camera (PRIME; Photometrics, AZ) and stored in a 16-bit TIFF format generated by Micro-manager software. In addition, DNA molecules were imaged using field emission SEM (FE-SEM; JSM-7100F, JEOL, Japan). Circular and supercoiled DNA molecules that appear as dots under the FM were confirmed under the SEM.
The length of circular and supercoiled DNA molecules was manually measured using imageJ. Length of 1 bp is commonly known to be 0.34 nm, however observed length depends on stretching of DNA molecules and may need case-specific conversion factor. For example, Kosar, et al. (2021) used conversion factor of 0.36 nm/bp which was calculated from internal standard. For DNA molecules less than 10 kb, fractional extensions are less than 80%. Therefore, we used plasmid with known length (5.2 kb) for correct measurement. Average length of the plasmid was 1233.3 ± 150.6 nm (n=37), which in turn tells that 1 bp is ~ 0.24 nm for ~ 5 kb DNA molecules (70.6% fractional extension).

Data analysis

Sorting and verifying mitochondrial contigs
BLAST 2.2.31+ was used to search for mitochondrial genes. Because mitochondrial gene sequences of the Stylonematophyceae were absent in the National Center for Biotechnology Information (NCBI) database, mitochondrial protein sequences from several red algae species were searched against assembled SPAdes contigs with e-value 1e-05 using tBLASTn. All the matched sequences were translated (Genetic code 4) and aligned against NCBI protein database (nr). Sequences that have eukaryotic taxa in top 100 matches were considered as candidate genes. Those that only had prokaryotic taxa in top 100 matches with significantly low identity or query coverage were also selected as possible candidates.
To exclude bacterial contigs from possible mitochondrial contigs, genomic features such as GC content, read coverage and tBLASTn result (top match and identity) of the contig were used as criteria for selection. CDSs of each contig were compared against NCBI protein database (nr) using default parameters. These candidate contigs were verified manually using phylogenetic analysis. Using translated CDS in candidate contigs as queries, protein sequences from nr database were searched by MMSeqs2 (Version: 330ea3684fd3f985d0127ffe8ca5b3f13053c619) with maximum sensitivity and e-value 1e-05.

Nuclear gene prediction
RNA-seq reads were mapped against the assembled nuclear genome of R. marinus using hisat2 (2.2.1) and STAR 2.7.7a (--outFilterScoreMinOverLread 0.45 --outFilterMatchNminOverLread 0.45). Mapping information was used as training set of ab initio gene models, performed using BRAKER 2.1.5. Completeness was measured using BUSCO 3.0.2 with the eukaryote_odb9 database. RAD52 was not found in the C. crispus proteome and contaminant assemblies were found in the transcriptome assembly of C. ornatum. Therefore, we chose to generate a transcriptome assembly and perform gene modeling using the available RNA-seq data. We used Trinity 2.11.0 to obtain the transcriptome assembly. cd-hit 4.8.1 was used to cluster sequences with similarity over 95% and predicted proteins were generated using Transdecoder 5.5.0. BUSCO values were: C. crispus, C:97.4% [S:27.4%, D:70.0%], F:2.6%, M:-0.0%, n:303; and C. ornatum, C:96.7% [S:20.5%, D:76.2%], F:1.3%, M:2.0%, n:303. Consequently, we predicted several novel genes that are not present in existing red algal data.

Comparative analysis of CDSs
The mitochondrial genomes of 23 red algae representing Cyanidiophyceae, Compsopogonophyceae, Porphyridiophyceae, Rhodellophyceae, Bangiophyceae and Florideophyceae were downloaded from NCBI nucleotide database (nt) and used for the comparison. Translated sequences of 11 CDSs (atp6, atp9, cob, cox1, cox2, cox3, nad1, nad2, nad4, nad5-f and nad5-s) were aligned by MAFFT 7.310 and concatenated. From the concatenated alignment, amino acid similarity were calculated by Geneious 10.2.3 using blosum62 matrix with threshold 1 as well as nucleotide identity. Maximum-likelihood phylogenetic tree was built using IQ-TREE 1.6.8. Optimal evolutionary models were automatically chosen after model selection. Pairwise dN/dS calculation was performed by ParaAT 2.0 and KaKs Calculator 2.0.
For species identification of seven Stylonematophyceae, we downloaded rbcL sequences of 16 Stylonematophyceae and one Compsopogonophyceae from NCBI and aligned with those of our samples using MAFFT 7.310. IQ-TREE 1.6.8 was used to construct a maximum likelihood phylogenetic tree. Optimal evolutionary models were automatically chosen after model selection.

Detecting mitochondrial tRNA, rRNA, and other sequences in the nuclear genome
We searched for tRNA genes using ARAGORN 1.2.38. These sequences were initially searched in long-read assemblies of R. marinus and C. ornatum using barrnap 0.9 and RNAmmer 1.2 but no matches were found. We then looked for raw reads reporting BLASTn hits on collected red algal rRNA sequences under diverse e-values, up to 100 but the results were not useful. Next, we looked for RNA transcripts of R. marinus.

Long-reads were mapped on each of assembled transcripts, collected, and assembled using minimap2 (2.17-r941) and miniasm 0.3 (r179). Transcripts whose assembled contig is predicted to have circular topology were sorted and alignments were manually inspected. LSU rRNA of C. ornatum was detected using BLASTn and LSU rRNA of R. marinus as a query but not in the case of SSU rRNA. SSU rRNA minicircle has a constant region that is shared among most of other minicircle in R. marinus. Thus, we collect reads that have BLASTn match against constant regions and filtered out reads that have BLASTn match against coding sequences. After the assembly (described above), only one contig that has a circular topology left, a SSU rRNA minicircle of C. ornatum. Polishing was performed using the procedure described above.
For coding genes, we downloaded gene sets of six red algae and the transcriptome assembly of two red algae (Supplementary Table S1). As described above, we predicted gene sets for C. crispus, Bangiopsis sp. CCMP1999, and C. ornatum. We aligned all the mitochondrial genes from 30 species against gene sets of each red alga using DIAMOND 0.9.36.137 to find EGT-derived genes. Then we aligned all the gene sets against the NCBI protein database (nr). Hit queries and subjects were aligned using MAFFT 7.310 and a phylogenetic tree was constructed using IQ-TREE 1.6.8.
Genes that control genome stability were selected based on existing data. Proteins were downloaded from NCBI and aligned against the red algal gene set using BLASTp (e-value 1e-03). Hit identity, alignment length, query length, and subject taxon from BLASTp results against NCBI database, as well as alignment and phylogenetic tree were taken into account to determine the presence of a gene. Specifically, for RAD52, RAD52 and its paralog/homolog protein RAD59, RTI1 and RAD22, as well as some other related proteins MGM101 and RDM1 were collected from the NCBI database for query and an e-value with a maximum of 10 was used. Seven additional red genomes were used for identification of RAD52, MSH1 and NTG1. Interproscan 5.52-86.0 was used for domain prediction.
Transit peptides were identified using target2.0 (-org pl). Statistical tests were performed using the Wilcoxon rank sum test in R 4.0.3.
Complete mitogenome sequences were used as BLASTn queries against nuclear genome to find NUMTs. Hits with e-value under 10-4 were considered as NUMTs.

For manuscripts utilizing custom algorithms or software that are central to the research but not yet described in published literature, software must be made available to editors and reviewers. We strongly encourage code deposition in a community repository (e.g. GitHub). See the Nature Portfolio guidelines for submitting code & software for further information.

Data

Policy information about availability of data

All manuscripts must include a data availability statement. This statement should provide the following information, where applicable:

- Accession codes, unique identifiers, or web links for publicly available datasets
- A description of any restrictions on data availability
- For clinical datasets or third party data, please ensure that the statement adheres to our policy

Data Availability
Short-read data generated in this study have been deposited in the NCBI Sequence Read Archive under BioProject PRJNA778797. Mitogenomes for two species and CDSs for other five species generated in this study have been deposited in the NCBI nucleotide database under accession numbers OK64388-OK643971, ON716284, ON716285, OP177696, and OP146131. Whole Genome Shotgun project of R. marinus generated in this study has been deposited at GenBank under the accession number JAMWBK000000000.1 and National Marine Genome Information Center of Korea (http://www.magic.re.kr) under the accession number MA00405. Source data are provided as a Source Data file. The other data generated in this study are deposited in DRYAD database (https://doi.org/10.5061/dryad.tqjq2bw0w). Accession numbers for published genetic data used in the study are provided in the Supplementary Data file.

Human research participants

Policy information about studies involving human research participants and Sex and Gender in Research.

Reporting on sex and gender	[N/A]
Population characteristics	[N/A]
Recruitment	[N/A]
Ethics oversight	[N/A]

Note that full information on the approval of the study protocol must also be provided in the manuscript.

Field-specific reporting

Please select the one below that is the best fit for your research. If you are not sure, read the appropriate sections before making your selection.

☐ Life sciences ☐ Behavioural & social sciences ☒ Ecological, evolutionary & environmental sciences

For a reference copy of the document with all sections, see nature.com/documents/nr-reporting-summary-flat.pdf

Ecological, evolutionary & environmental sciences study design

All studies must disclose on these points even when the disclosure is negative.

Study description

We confirmed that mitogenomes of Stylonematophyceae are circularly fragmented (i.e., minicircles), which are reported only in few lineages such as lice (mitochondria), diplonemida (mitochondria) and dinoflagellate (plastid). Minicircles consist of "cassette" that harbors CDS and gene-specific non-coding region (NCR) and "constant" region that harbors large, species-specific, and tandem repeat containing NCR. Sequences were highly diverged and many genes were lost compare to the other red algae, consistent with co-location for redox regulation of gene expressio (CoRR) hypothesis. Some of lost genes were transferred into nuclear genome. Presence of chimeric reads resulted from merger of two minicircles, not from sequencing error, provide evidence of recombination. Together with unique gene inventory that controls mitogenome stability, recombination might drove the transition from typical mitogenomes into minicircles.

Research sample

Culture strains of Tsunamia transpacifica JAW4874, Rufusia pilicola O7031, Stylonema alsidii JAW4424, Chroodactylon ornatum JAW4256, Chroothece mobilis SAG104.79, Rhodosorus marinus CCMP1338 and Bangiopsis subsimplex UTEX LB2854 were obtained from J.A. West (School of Biosciences 2, University of Melbourne, Parkville, Victoria 3010, Australia), F.D. Ott (905 NE Hilltop Drive, Topeka, Kansas 66617, USA), The Culture Collection of Algae at Goettingen University, Germany (SAG), The National Center for Marine Algae and Microbiota (NCMA), and the Culture Collection of Algae at The University of Texas at Austin, USA (UTEX), respectively.

Sampling strategy

[N/A] In this study, there was no experiment to determine sample-size.

Data collection

Whole genome sequencing and genome assembly
Library preparation and whole genome sequencing for both short-read and long-read sequencing were carried out by DNA Link Inc. (Seoul, Korea). For short-read sequencing, libraries were prepared using the Truseq Nano DNA Prep Kit (550 bp Protocol) and sequencing was done with the Illumina HiSeq2500 platform according to the protocol using 100 bp paired-end reagents. Long-read sequencing was carried out with Oxford Nanopore platform (ONT GridION) for R. marinus and the Pacific Biosciences (PacBio) High-Fidelity (HiFi) sequencing platform for C. ornatum. RNA-seq for R. marinus was done with the Illumina NovaSeq600 platform. The raw data from short-read sequencing were assembled using SPAdes 3.14.1 with '—careful' pipeline option and those from long-read sequencing were assembled using NextDenovo 2.5.0 for nuclear genome of R. marinus. Assembled NextDenovo contigs were polished 3 times with Pilon 1.22 using short-read mapping data generated by bowtie2 2.3.5.1. For mitogenome assemblies using long-read data, reads that have BLAST hits to mitochondrial CDS were used. The program miniasm 0.3 (r179) was used to identify the R. marinus mitogenome and IPA 1.3.1 was used for C. ornatum. In addition, reads that had BLAST hits to the NCR were used to search for "empty" minicircle reads that do not contain a CDS, however, no contigs were assembled, meaning the collected reads are just fragments of CDS-containing reads. Because minicircles share long conserved region that short-reads cannot discriminate, we used long-read data and NextPolish 1.4.0 to polish the miniasm-derived contigs. We did not perform polishing on IPA contigs, because HiFi sequencing generates extremely accurate reads. Remaining SNPs and ambiguities were manually corrected using mapping data of long-reads containing CDS. For C. ornatum, each sequence from step 10 (10-assemble/p_ctg.fasta) was considered as a minicircle sequence, because the following step of the IPA assembler (polish and purge dups) did not function correctly.
For the short-read data, sorted and verified mitochondrial genes (see below) were used as seeds for NOVOplasty 4.2. Using Geneious (Biomatters, Auckland, New Zealand), generated NOVOplasty contigs were then de novo assembled. Assembled contig that codes any of mitochondrial genes was considered as part of mitochondrial genome. Those contigs were polished (-SNP & Indel) with Pilon 1.22, using short-read mapping data generated by bowtie2 2.3.5.1. Trinity 2.11.0 was used to assemble RNA sequencing data.

Polymerase chain reaction (PCR) and quantitative PCR (qPCR)
R. marinus total DNA was used to confirm hetero-concatemers and primers were designed to target ends of CDSs and toward the NCR, so that NCRs were amplified. R. marinus cDNA was used to confirm trans-spliced nad5 transcript and primers were designed to target 3' end of nad5-f and 5' end of nad5-s. R. marinus cDNA synthesis was performed using First Strand cDNA Synthesis kit (random hexamer primer; Thermo Scientific, Massachusetts, USA). PCR was performed using AccuPower® PCR PreMix kit (BIONEER, Daejeon, Korea). All primer designs were done using a modified version of Primer3 (2.3.7) built in Geneious 10.2.3. PCR conditions consisted of initial denaturation at 95°C for 3 min, followed by 35 cycles of denaturation at 95°C for 30 sec, annealing at 55°C for 30 sec, extension at 72°C, and a final 7 min extension step at 72°C. Extension steps take 4.5 min for the former and 1 min for the latter. PCR products were purified with LaboPassTM PCR kit (Cosmo Genetech, Seoul, Korea). Purified PCR products were sequenced using Sanger method by Macrogen Inc. (Seoul, Korea).
The SsoFastTM EvaGreen® Supermix (Bio-Rad, California, USA) was used for the qPCR assays (two replicates) that were run on a CFX96TM system (Bio-Rad, California, USA). Primers were designed to amplify gene-specific 150 bp fragments and were tested in advance to check for primer-dimer formation in no-template control (NTC). Each tube contained 5 µl of supermix, 0.2 µl of forward and reverse primers, 3.6 µl of nuclease free deionized water, and 1 µl of template DNA (final volume 10 µl). Probes for Southern hybridization were used as template DNAs of standard (see Supplementary Table S2 for more information). Starting from concentration of 1ng/µl, seven 10-fold serial dilution series were prepared. Starting concentration of standard sample of sdhB was 0.01 ng/µl because sdhB is EGT-derived gene. For target samples, 0.06 ng of genomic (g)DNA extracted using the CTAB method were shaken. Concentration of gDNA was measured using Qubit® 2.0 Fluorometer and QubitTM dsDNA BR Assay Kit (Invitrogen, Massachusetts, USA) and all samples underwent the same treatment. Quantitation cycle (Cq) values were calculated in Bio-Rad CFX Manager 2.1 (Cq determination mode=Single Threshold). Copy number and PCR efficiency were calculated using equations from 134 and 135, respectively. qPCR conditions consisted of initial denaturation at 95°C for 3 min, followed by 50 cycles of denaturation at 95°C for 5 sec, annealing and extension at 60°C for 15 sec.

Probe synthesis
All fragments for the minicircle DNA genes (atp6, atp9, cob, cox1, cox2, cox3, nad1, nad5-s, and nad5-f), LSU rDNA gene, and the sdhB nuclear gene sequences were prepared from gDNA with specific primers by using PCR and were purified using the LaboPassTM PCR kit (Cosmo Genetech, Seoul, Korea) prior to labeling. The digoxigenin (DIG)-labeled probes for Southern blot were synthesized using the DIG-High Prime DNA Labeling and Detection Starter Kit I (Roche Diagnostics, Mannheim, Germany), according to the

manufacturer’s instructions.

Southern blot analysis
For the Southern blot analysis, 1 µg of total DNA from of R. marinus was either undigested or digested with each minicircle-suitable restriction enzyme (Supplementary Fig. 2b). The digestion products were separated using 1% agarose gel electrophoresis in TAE buffer and transferred overnight to a positively charged nylon membrane (Cat. No. 11209299001, Sigma-Aldrich, St. Louis, MO) through capillary blotting with 10X SSC. After transfer, the membrane was auto-crosslinked using the Stratagene UV-Stratalinker. The crosslinked membrane was prehybridized, hybridized with the DIG-labeled probes, and then washed. Finally, the hybridized DNA probes were immunodetected with anti-digoxigenin-AP (Fab fragments) and visualized with the colorimetric substrates NBT/BCIP using the DIG-High Prime DNA Labeling and Detection Starter Kit I (Roche Diagnostics, Mannheim, Germany) according to the manufacturer's instructions. Blot images were stored by photocopying the wet filters.

Microchannel and Positive surface preparation
Polydimethylsiloxane (PDMS) devices and positively charged surfaces were prepared as previously described. In particular, microchannel template was utilized to create two layers on a silicon wafer through repeated photolithography procedures, following the protocol specified in the Kayaku Advanced Materials SU-8 2000 datasheet. First, a silicon wafer was spin-coated with a 20 µm layer of SU-8 2015 photoresist (Kayaku Advanced Materials, Massachusetts, USA) using a spin coater (Midas System SPIN-1200D, Daejeon, Korea). Subsequently, the spin-coated wafer was exposed to 350 nm radiation with an aligner (Midas System MDA-400LJ, Daejeon, Korea) through a mask and developed using an SU-8 developer (Kayaku Advanced Materials, Massachusetts, USA). Next, SU-8 TF 6002 was spin-coated as a second layer on top of the first layer. Since SmartPrint (SmartForce Technologies, La Tronche, France) is compatible with g-line photoresists, SU-8 TF 6002 was used. Following fabrication of the template wafer, the outlet port was created by attaching a tube to the peak area of the triangular section. At last, microchannel template was placed onto a silicon wafer. Then, a mixture (10:1 wt ratio) of PDMS pre-polymer and curing agent (K1 solution, Gwangmyeong, Korea) was poured onto it and incubated at 65 °C for 12 hours. The resulting PDMS layer was peeled from the wafer, and a chamber was created by physically punching a channel into it. The PDMS microchannel underwent oxidation for 30 seconds at 100 W in an air plasma generator (Femto Science Cute Basic, Korea). Finally, the PDMS device was washed and stored in deionized water.
Silicon wafers (Wafer market, Yongin, Korea) were purchased with a 30 nm SiO2 layer on top. To eliminate the polymer coating, oxidized silicon wafers and glass coverslips were arranged in a Teflon rack and soaked in piranha etching solution (30:70 v/v H2O2/H2SO4) for 3 hours. The wafers and coverslips were washed thoroughly with deionized water. Neutral pH of 7 was achieved and confirmed by pH paper. Subsequently, the wafers and coverslips were sonicated in deionized water for 30 minutes, followed by another round of rinsing with deionized water, to uncover the piranha surface. In the end, solutions with a concentration of 1.1 mM were prepared by adding 150 µl of Q-siloxane in 50% methanol to 250 mL of deionized water. Wafers and coverslips were incubated at 65 °C and 100 rpm for 16 h. Finally, they were rinsed three times with 99.9% ethanol and stored in 99.9% ethanol.

DNA molecule visualization under FM and SEM
DNA molecules pre-mixed with FP-DBP were stained with 5 % polyvinylpyrrolidone (PVP, molecular weight (MW): 40 000) solution. Stained DNA molecules were elongated and immobilized on a positively charged surface using a PDMS microfluidic device. DNA molecules were imaged under a FM. The microscopy system consisted of an inverted microscope (Olympus IX70, Japan) equipped with 100× Olympus UPlanSApo oil immersion objectives and an illuminated LED light source (SOLA SM 2 light engine, Lumencor, OR). Fluorescence images were captured using a scientific complementary metal-oxide semiconductor (sCMOS) camera (PRIME; Photometrics, AZ) and stored in a 16-bit TIFF format generated by Micro-manager software. In addition, DNA molecules were imaged using field emission SEM (FE-SEM; JSM-7100F, JEOL, Japan). Circular and supercoiled DNA molecules that appear as dots under the FM were confirmed under the SEM.
The length of circular and supercoiled DNA molecules was manually measured using imageJ. Length of 1 bp is commonly known to be 0.34 nm, however observed length depends on stretching of DNA molecules and may need case-specific conversion factor. For example, Kosar, et al. (2021) used conversion factor of 0.36 nm/bp which was calculated from internal standard. For DNA molecules less than 10 kb, fractional extensions are less than 80%. Therefore, we used plasmid with known length (5.2 kb) for correct measurement. Average length of the plasmid was 1233.3 ± 150.6 nm (n=37), which in turn tells that 1 bp is ~ 0.24 nm for ~ 5 kb DNA molecules (70.6% fractional extension).

Timing and spatial scale: [N/A] In this study, there was no experiment to require timing and spatial scale.

Data exclusions: [N/A] No data were excluded from the analyses.

Reproducibility: Microscope images of cultures were taken multiple times (n = 10). Southern blot was performed only once. FM images were taken only once. SEM images of digested and undigested DNA are taken 12 and 15 times, respectively.

Randomization: [N/A] In this study, there was no experiment consider to randomization.

Blinding: [N/A] There was no blinding information in this study.

Did the study involve field work? ☐ Yes ☒ No

Reporting for specific materials, systems and methods

We require information from authors about some types of materials, experimental systems and methods used in many studies. Here, indicate whether each material, system or method listed is relevant to your study. If you are not sure if a list item applies to your research, read the appropriate section before selecting a response.

Materials & experimental systems

n/a	Involved in the study
☒	☐ Antibodies
☐	☒ Eukaryotic cell lines
☒	☐ Palaeontology and archaeology
☒	☐ Animals and other organisms
☒	☐ Clinical data
☒	☐ Dual use research of concern

Methods

n/a	Involved in the study
☒	☐ ChIP-seq
☒	☐ Flow cytometry
☒	☐ MRI-based neuroimaging

Eukaryotic cell lines

Policy information about cell lines and Sex and Gender in Research

Cell line source(s)	Culture strains of Tsunamia transpacifica JAW4874, Rufusia pilicola O7031, Stylonema alsidii JAW4424, Chroodactylon ornatum JAW4256, Chroothece mobilis SAG104.79, Rhodosorus marinus CCMP1338, and Bangiopsis subsimplex UTEX LB2854 were obtained from J.A. West (School of Biosciences 2, University of Melbourne, Parkville, Victoria 3010, Australia), F.D. Ott (905 NE Hilltop Drive, Topeka, Kansas 66617, USA), The Culture Collection of Algae at Göttingen University, Germany (SAG), The National Center for Marine Algae and Microbiota (NCMA), and the Culture Collection of Algae at The University of Texas at Austin, USA (UTEX), respectively.
Authentication	For species identification of seven Stylonematophyceae, we downloaded rbcL sequences of 16 Stylonematophyceae and one Compsopogonophyceae from NCBI and aligned with those of our PCR-sequenced samples using MAFFT 7.310. IQ-TREE 1.6.8 was used to construct a maximum likelihood phylogenetic tree. Optimal evolutionary models were automatically chosen after model selection.
Mycoplasma contamination	[N/A] The cell lines were not tested for mycoplasma contamination
Commonly misidentified lines (See ICLAC register)	[N/A]
