# Supplementary figures and images for: Origin of minicircular mitochondrial genomes in red algae
Source: Nat Commun. 2023 Jun 8;14:3363. doi: 10.1038/s41467-023-39084-2 (PMC10250338; doi:10.1038/s41467-023-39084-2)

48.5 kb  
15 kb  
10 kb  
8 kb  
6 kb  
5 kb  
4 kb  
3 kb

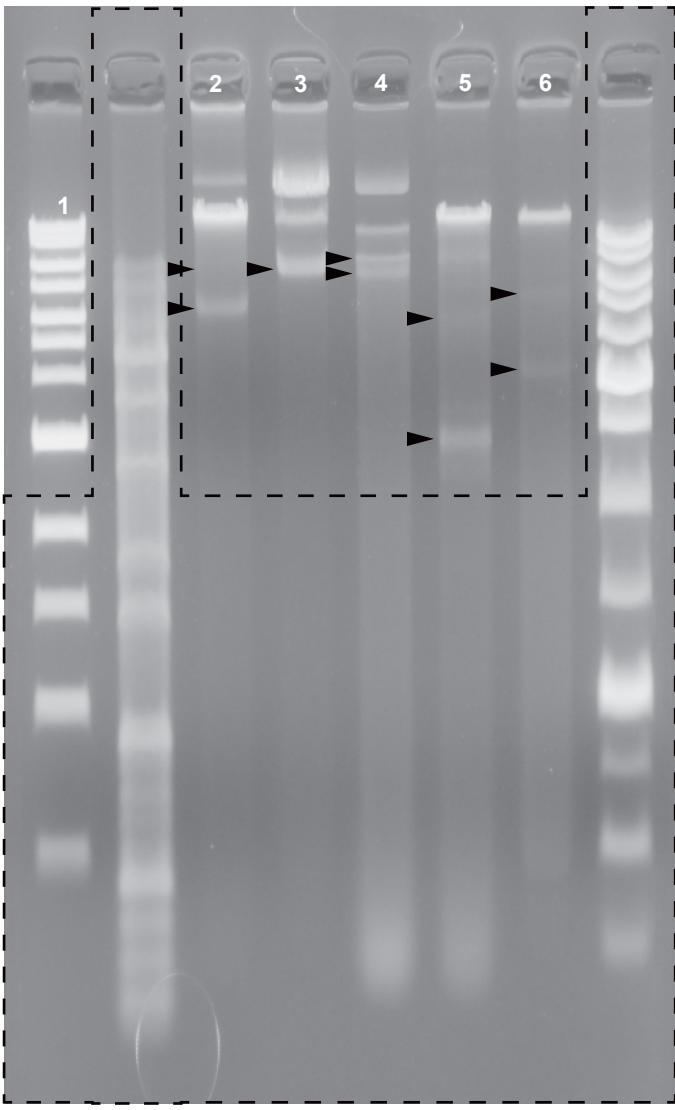

Supplement: Supplementary file 5 — Source Data [file 41467_2023_39084_MOESM5_ESM.zip › Uncropped_image/Fig1h.pdf]

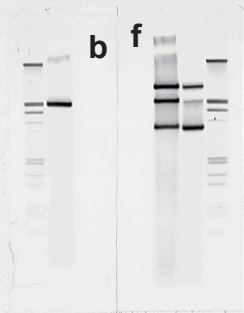

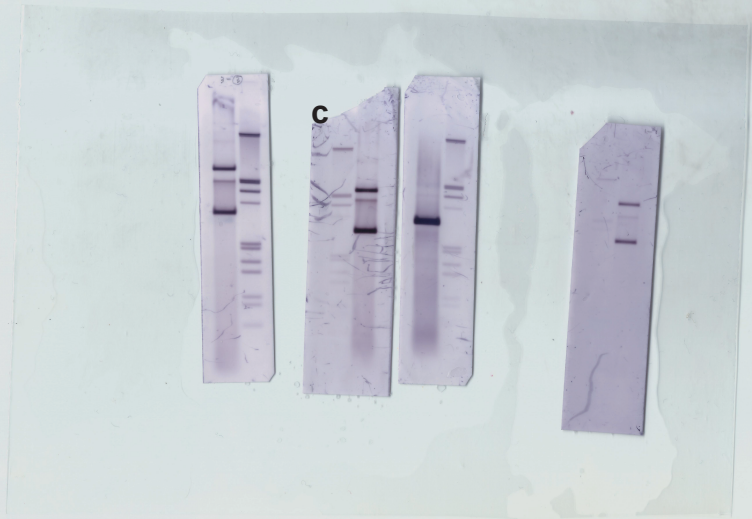

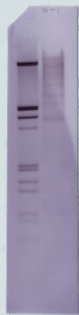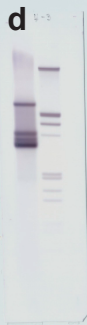

h h e

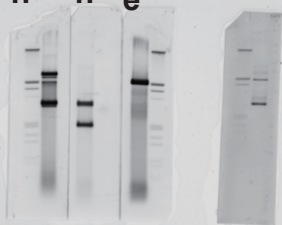

Supplement: Supplementary file 5 — Source Data [file 41467_2023_39084_MOESM5_ESM.zip › Uncropped_image/Fig3_Supplementary_Fig6.pdf]
